# Supplementary material for: Accurate quantification of copy-number aberrations and whole-genome duplications in multi-sample tumor sequencing data
Source: Nat Commun. 2020 Sep 2;11:4301. doi: 10.1038/s41467-020-17967-y (PMC7468132; doi:10.1038/s41467-020-17967-y)
Supplement: Supplementary file 1 — Supplementary Information [file 41467_2020_17967_MOESM1_ESM.pdf]

Supplementary Information

Accurate quantification of copy-number aberrations and whole-genome duplications in multi-sample tumor sequencing data

Zaccaria & Raphael

Contents

Supplementary Figures 4

1 Interpretation of many clusters as subclonal CNAs vs. WGD 4

2 Tumor clones with different CNAs, tumor ploidy, and clone proportions in mixed samples result in the same values of RDR and BAF 5

3 Read counts are unaffected by either the presence or absence of a WGD 6

4 The proportions of DNA sequencing reads and clone proportions are generally different 7

5 Read counts simulated according to either clone proportions or genome-length corrected proportions are different 8

6 Simulating bulk tumor sequencing data with MASCoTE 9

7 Tumor ploidy of all simulated bulk tumor samples 10

8 HATCHet outperforms existing methods in the inference of total copy numbers, their proportions, and WGDs . . 11

9 Precision and recall of total copy numbers with fixed parameters 12

10 Precision and recall of total copy numbers per segment of at least 50kb size with fixed parameters 13

11 Precision and recall of copy-number states with fixed parameters 14

12 Average amplification-deletion error per genome position with fixed parameters 15

13 Relative error of tumor purity 16

14 Relative error of tumor ploidy 17

15 Precision and recall of total copy numbers with free parameters 18

16 Precision and recall of total copy numbers per segment of at least 50kb size with free parameters 19

17 Precision and recall of copy-number states with free parameters 20

18 Average amplification-deletion error per genome position with free parameters 21

19 Consistency in WGD predictions on simulated data among different methods 22

20 HATCHet outperforms FALCON in deriving fractional copy numbers 23

21 HATCHet's copy numbers are consistent with published single-cell copy-number profiles of 4 breast cancer patients 24

22 HATCHet's copy numbers are consistent with published single-cell copy-number profiles of 4 breast cancer patients 25

23 Total copy-number profiles of tumor clones in prostate cancer patients 26
24 Total copy-number profiles of tumor clones in pancreas cancer patients 27
25 Classification of sample- or tumor-specific clonal and subclonal clusters 28
26 Battenberg identifies subclonal genomic regions which are not supported by measured data in sample A10-A . . . 29
27 Battenberg overestimates the presence of subclonal CNAs in the sample A10-C of prostate cancer patient A10 . . 30
28 Segment-specific and clone-specific models of CNAs 31
29 HATCHet uses six times less parameters than Battenberg on average across all solutions of the prostate cancer dataset 32
30 Fraction of the genome with CNAs and subclonal CNAs inferred by HATCHet and Battenberg for all samples of the prostate cancer dataset 33
31 HATCHet achieves an error between the observed and estimated RDRs similar or lower than Battenberg 34
32 ReMixT and Battenberg infer extensive subclonal CNAs and shared subclonal CNAs in all samples of prostate cancer patients 35
33 Published copy numbers derived from Control-FREEC for pancreas cancer patients Pam01 and Pam02 are inconsistent across samples and miss subclonal CNAs and WGDs 36
34 Fraction of the genome with CNAs and subclonal CNAs inferred by HATCHet and Control-FREEC for all samples of the pancreas cancer dataset 37
35 HATCHet has a lower error between the observed and estimated RDRs than Control-FREEC 38
36 HATCHet uses three times less parameters than Control-FREEC for the results on the pancreas cancer dataset . . 39
37 Battenberg infers shared subclonal CNAs in every sample of the prostate dataset and suggests polyclonal migrations in 9/10 patients 40
38 HATCHet infers tumor clones consistent with previous reports of polyclonal origin of metastasis in 3 prostate cancer patients 41
39 HATCHet infers tumor clones consistent with previous reports of limited heterogeneity and with unreported presence of polyclonal migrations in the pancreas cancer patients. 42
40 HATCHet identifies sample-specific states in a minority of tumor samples 43
41 HATCHet identifies only few samples with sample-specific copy-number states in contrast to previous analyses . . 44
42 Battenberg and Control-FREEC infer many and large sample-specific CNAs in all chromosomes of every cancer patient 45
43 HATCHet identifies multiple tumor subclones shared across samples from the same patient, suggesting polyclonal origin of metastasis in some prostate and pancreas cancer patients 46
44 The clonality distance supports HATCHet's inference of subclonal CNAs in the prostate cancer dataset 47
45 The clonality distance supports HATCHet's inference of subclonal CNAs in the pancreas cancer dataset 48
46 HATCHet predicts WGDs consistently across all samples from the same prostate cancer patient 49
47 HATCHet infers simpler solutions and predicts a WGD consistently across all samples of prostate cancer patient A29 50
48 The clonality distance supports HATCHet's predictions of WGDs in the prostate and pancreas cancer datasets . . 51
49 HATCHet identifies WGDs in three of four pancreas cancer patients 52
50 Number of somatic SNVs and small indels inferred from the samples of the prostate and pancreas cancer datasets . 53
51 HATCHet consistently estimates predicted VAFs with lower errors than Battenberg on all prostate cancer patients 54

52 HATCHet consistently estimates predicted VAFs with lower errors than Control-FREEC on all pancreas cancer patients . . . 55
53 Observed VAF of somatic mutations for all samples of the prostate cancer patients . . . 56
54 Somatic mutations non-explained by HATCHet have low VAF in the samples of the prostate cancer patients . . . 57
55 Observed VAF of somatic mutations for all samples of the pancreas cancer patients . . . 58
56 HATCHet explains most of the somatic mutations in the samples of 3/4 pancreas cancer patients . . . 59
57 Local clustering and global clustering of genomic regions . . . 60

Supplementary Tables **61**

1 Comparison of HATCHet and existing methods for copy-number deconvolution . . . 61
2 Parameters inferred by HATCHet . . . 62
3 Model selection parameters and their default values . . . 63

Supplementary Methods **64**

1 On the model of fractional copy numbers, tumor ploidy, and tumor purity . . . 64
2 HATCHet: global clustering along the genome and across samples . . . 67
3 HATCHet: estimation of fractional copy numbers . . . 70
4 HATCHet: constrained and distance-based allele-specific copy-number factorization . . . 73
5 HATCHet: joint selection of number of clones and WGD . . . 79
6 MASCoTE: simulating sequencing reads from multiple mixed samples . . . 81

Supplementary Notes **84**

1 Experimental setup for simulated data . . . 84
2 Metrics for evaluating performance on simulated data . . . 87
3 Additional results on simulated data with fixed parameters . . . 92
4 Additional results on simulated data with free parameters . . . 93
5 Comparing HATCHet's results with single-cell copy-number profiles . . . 94
6 Experimental setup for cancer data . . . 95
7 Comparing fractions of the genome with CNAs and subclonal CNAs . . . 96
8 Measuring data fitting and number of parameters . . . 97
9 Comparing HATCHet with Battenberg and ReMixT on prostate cancer patients . . . 98
10 HATCHet enables the identification of tumor clones shared across samples . . . 99
11 Analysis of heterogeneity across samples through sample-specific copy-number states . . . 99
12 Assessing the presence of subclonal CNAs through clonality distance . . . 101
13 Analysis of divergent predictions of WGD across multiple samples . . . 103
14 Analysis of somatic mutations . . . 104

Supplementary References **107**

Supplementary Figures

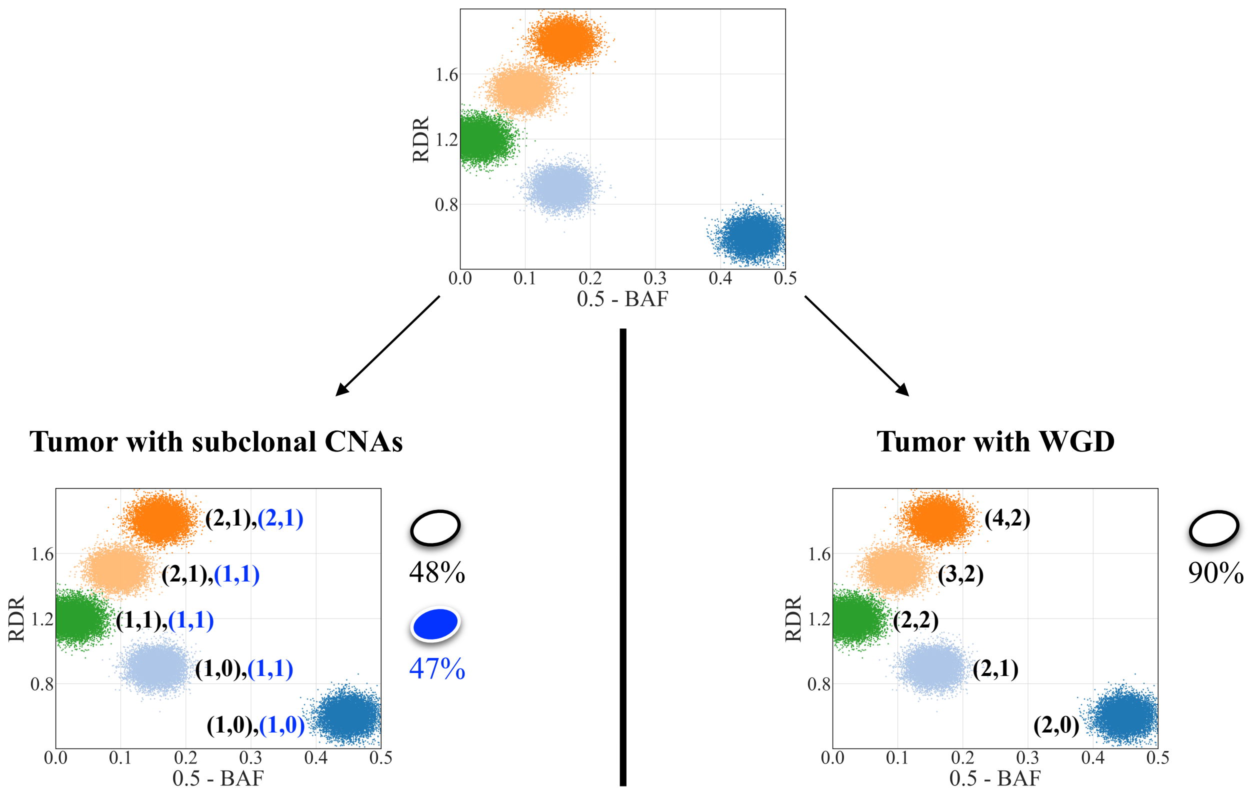

Supplementary Fig. 1: Interpretation of many clusters as subclonal CNAs vs. WGD. RDRs and BAFs for five clusters of genomic bins (colors) have two alternate explanations. (Bottom left) The first explanation has two distinct tumor clones (white and blue) with nearly the same clone proportions (48% and 47%) and the normal clone in low proportion (5%). In this explanation, three clusters (green, dark orange, and dark blue) correspond to clonal CNAs with the same indicated copy-number state in both clones and the two remaining clusters (light orange and light blue) correspond to subclonal CNAs with different copy-number states as indicated in each clone. (Bottom right) The second explanation has a single tumor clone (white) containing a WGD with the tumor clone in high proportion (90%) and the normal clone in low proportion (10%). In this explanation, all five clusters correspond to clonal CNAs.

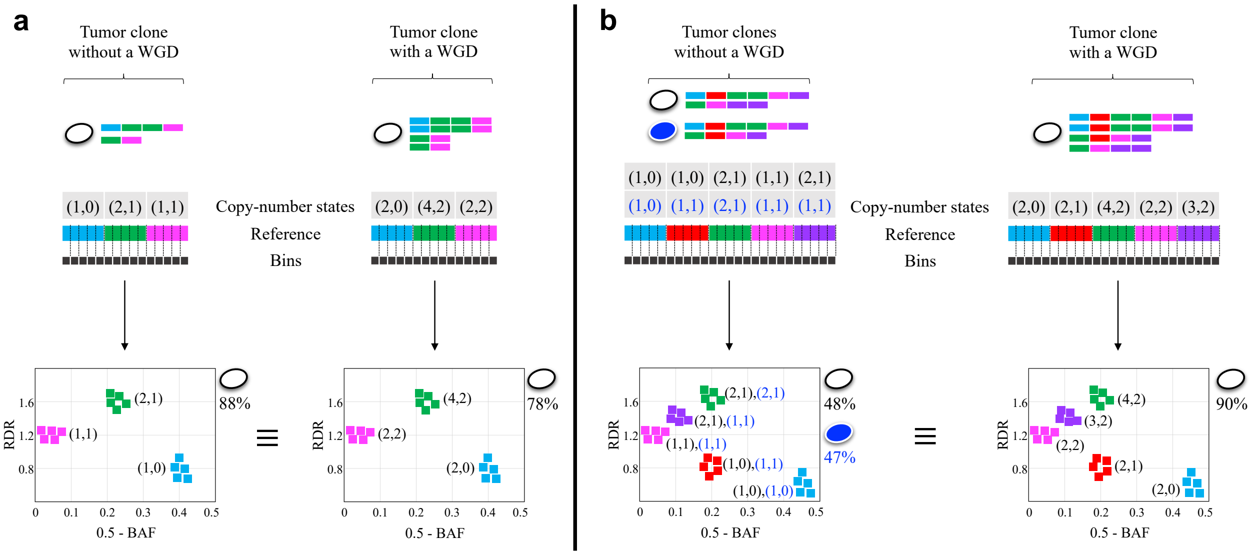

Supplementary Fig. 2: Tumor clones with different CNAs, tumor ploidy, and clone proportions in mixed samples result in the same values of RDR and BAF. a, (Left) a tumor clone has a genome composed of 3 segments (colors) whose copy-number states are correspondingly reported below. The expected RDR and BAF for each bin of the reference genome (squares colored as corresponding clusters) are computed assuming that the clone is the only tumor clone present in a sample with tumor purity equal to 88% and each cluster is labeled by the corresponding copy-number states. (Right) a tumor clone has the same genome as the tumor clone in (Left) but additionally affected by a WGD. The expected RDR and BAF for each bin of the reference genome are computed assuming that the clone is the only tumor clone present in a sample with tumor purity equal to 78% and we label each cluster with the corresponding copy-number states. The RDR and BAF of the clusters are approximately the same in the two cases. **b**, (Left) two tumor clones have genomes composed of 5 clusters (colors) whose copy-number states are correspondingly reported below. The expected RDR and BAF for each bin of the reference genome (squares colored as corresponding clusters) are computed assuming that the two tumor clones are present in a sample with clone proportions equal to 48% and 47%, respectively. Each cluster is labeled by the corresponding copy-number states. (Right) a tumor clone has a genome affected by a WGD and composed of 5 clusters (colors) whose copy-number states are correspondingly reported below. The expected RDR and BAF for each bin of the reference genome are computed assuming that the clone is the only tumor clone present in a sample with purity of 90% and each cluster is labeled by the corresponding copy-number state. The RDR and BAF of the clusters are approximately the same in the two cases.

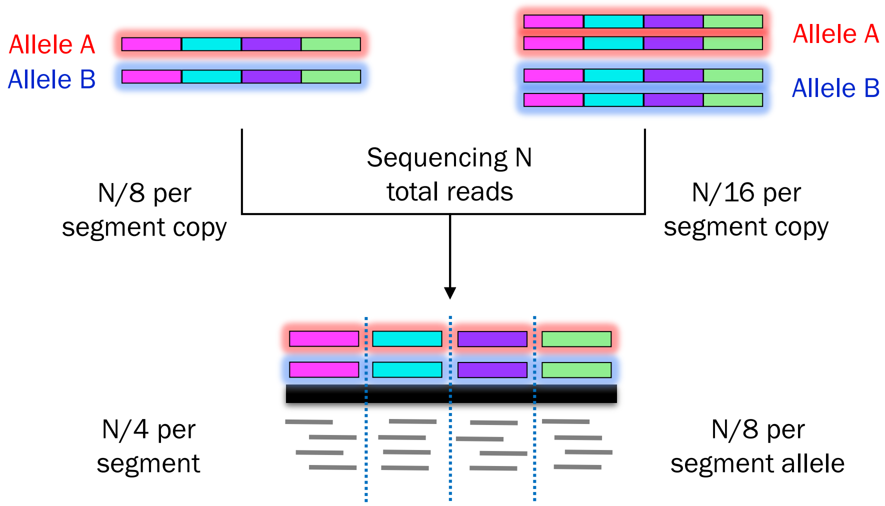

Supplementary Fig. 3: Read counts are unaffected by either the presence or absence of a WGD. The genome of a clone with two alleles (red and blue shadows) of 4 segments is represented in the left part of the figure. The same genome additionally affected by a WGD is represented in the right part of the figure; in this case the WGD doubles every copy of the segments. We assume to uniformly sequence N total reads from each of the two genomes. For simplicity, we also assume that every segment contains the same number of genomic positions, has the same length ℓ, and we ignore the read length. We obtain $\frac{N}{8}$ total reads from each copy of the segments of the genome without a WGD because the length of each segment's copy is ℓ and the genome length is equal to 8ℓ. Also, we obtain $\frac{N}{16}$ total reads from each copy of the segments of the genome with a WGD because the length of each segment's copy is ℓ and the genome length is equal to 16ℓ. While a WGD doubles the genome length yielding a read count per copy reduced by half, the number of copies of each segment is doubled and therefore the read counts obtained per each segment (and per each allele of the segment) is the same in the two cases.

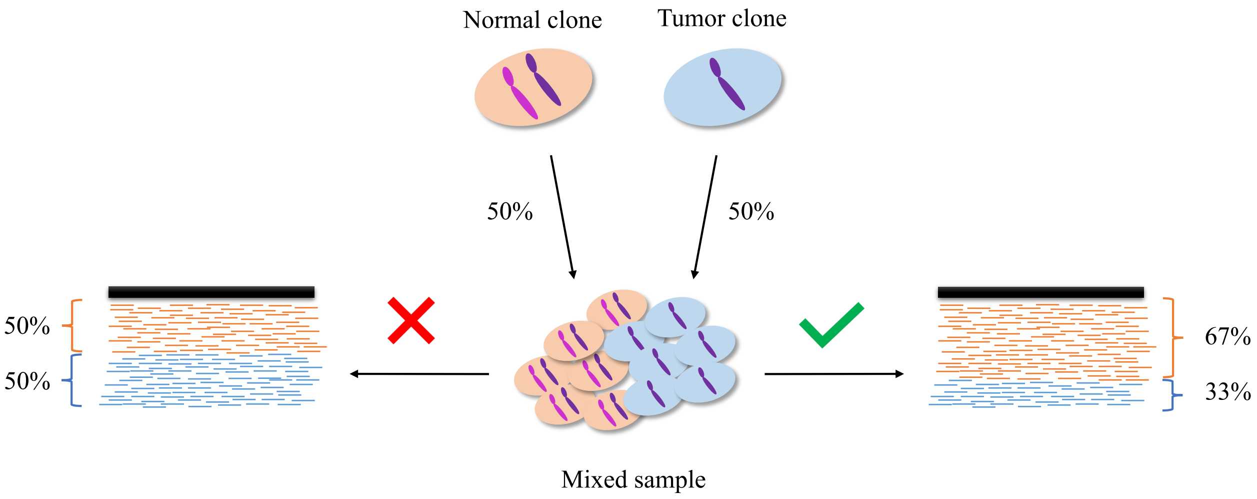

Supplementary Fig. 4: The proportions of DNA sequencing reads and clone proportions are generally different. A normal clone has two chromosomes (purple and magenta), and a tumor clone loses one of these and remains with only one chromosome (purple). A mixed sample comprises half of the cells from the normal clone and half of the cell from the tumor clone; every clone proportion is hence equal to 50%. Assuming to sequence a certain number of reads from the DNA of the cells in the mixed sample, one can think that half of the reads belongs to the normal clone and half of the reads from the tumor clone. However, the normal cells have a genome that is twice longer than the one of tumor cells and we thus expect that more reads come from the normal clone in DNA sequencing. In fact, the proportion of the reads belonging to a clone in DNA sequencing corresponds to the proportion of its DNA content which is related to the genome length of the clone; in particular, the proportion of reads simultaneously depends on the corresponding clone proportion and genome length. The correct proportion of reads belonging from the normal and tumor clone is therefore equal to 67% and 33%, respectively.

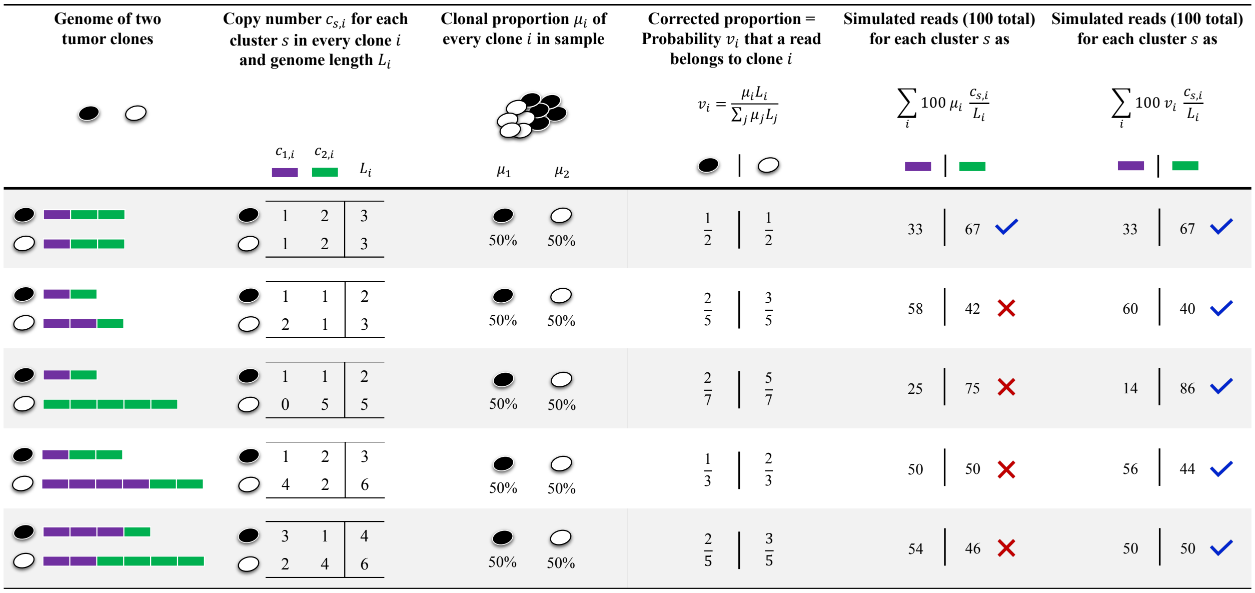

Supplementary Fig. 5: Read counts simulated according to either clone proportions or genome-length corrected proportions are different. Five different genomes for two clones are composed of different number of copies for two segments (purple and green) such that $c_{s,i}$ is the total copy number of segment s in clone i. In each case the genome length L_i of a clone i is computed as the sum of all copies for the corresponding segments which are assumed to have a length equal to 1, for simplicity. We aim to simulate the read counts obtained from a sample corresponding to a mixture of the two clones in equal proportions such that the clone proportion u_i of each clone is equal to 0.5. When simulating according to clone proportions, the proportion of reads belonging to clone i is u_i. However, the corrected proportion v_i of reads from clone i corresponds to the probability that a read belongs to i and is computed as the ratio between the genome length L_i weighted by the corresponding clone proportion u_i and the weighted sum of the genome lengths of all clones, i.e. $\frac{u_i L_i}{\sum_j u_j L_j}$. As such, assuming a total of 100 reads are simulated uniformly along the genome, the total number of reads simulated for each segment s correspond to $\sum_i 100 u_i \frac{c_{s,i}}{L_i}$ and $\sum_i 100 v_i \frac{c_{s,i}}{L_i}$ when simulating according to the clone proportions or corrected proportions, respectively. The simulated read counts in the two cases are equal only when the genome lengths of the two clones are the same (first row), while they are substantially different in all the others when clones have both little (second row) or large (third row) differences in the genome lengths. Notably, the read counts in the last two combinations of genomes are equal when considering the clone proportions u_i but they are different when considering the corrected proportions v_i in one case (fourth row), and vice-versa in the other case (fifth row).

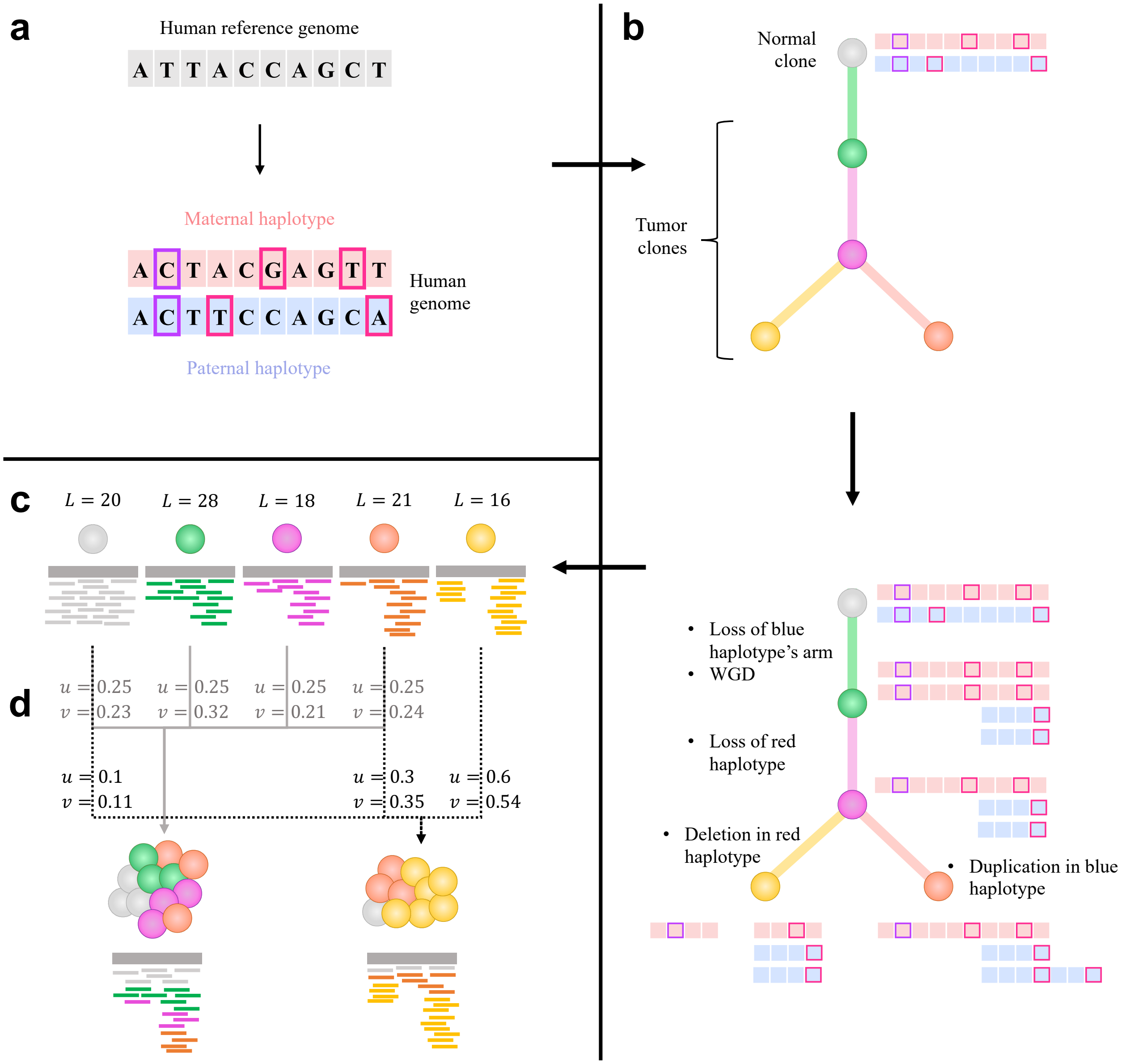

Supplementary Fig. 6: Simulating bulk tumor sequencing data with MASCoTE MASCoTE (Multiple Allele-specific Simulation of Copy-number Tumor Evolution) simulates sequencing reads of multiple mixed samples according to the genome lengths and proportions of all clones. The simulation framework of MASCoTE is composed of four steps. **a**, MASCoTE simulates a diploid human genome by inserting homozygous (purple square) and heterozygous (magenta squares) germline SNPs in the two haplotypes (red and blue haplotypes). **b**, MASCoTE simulates the genomes of multiple tumor clones that include CNAs and WGDs. First, MASCoTE generates a random phylogenetic tree which describes the tumor evolution; the phylogenetic tree is rooted in the normal diploid clone (gray node) and every other node corresponds to a tumor clone (green, fuchsia, orange, and gold nodes). Next, MASCoTE simulates different kinds of CNAs for every branch, including aberrations of whole chromosomes or chromosomal's arms, and focal duplications and deletions. MASCoTE also simulates a WGD in the trunk of the phylogeny. As such, the CNAs and WGDs in each branch are applied in arbitrary order to the genome of the parent clone to obtain the genome of the child clone such that the diploid human genome generated in (**a**) is assigned to the normal diploid clone. **c**, MASCoTE simulates sequencing reads from the genome of every clone and computes the corresponding genome lengths (L). **d**, MASCoTE simulates multiple mixed samples according to given clone proportions (u) by computing the corrected proportions of sequencing reads belonging to each clone (v) and by mixing the reads accordingly.

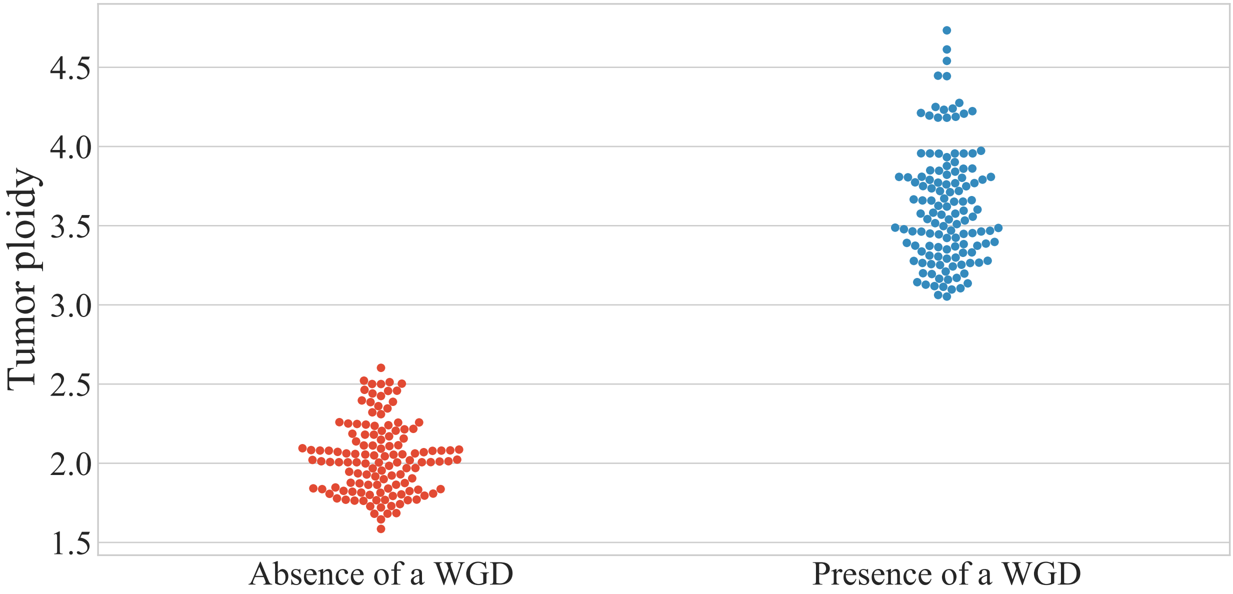

Supplementary Fig. 7: Tumor ploidy of all simulated bulk tumor samples. The entire collection of all simulated bulk tumor samples comprises 128 samples without a WGD and 128 samples with a WGD. The tumor ploidy of each sample is computed as the average copy number for all tumor clones present in the sample and weighted by the proportion of the corresponding tumor clone relative to the tumor purity of the sample.

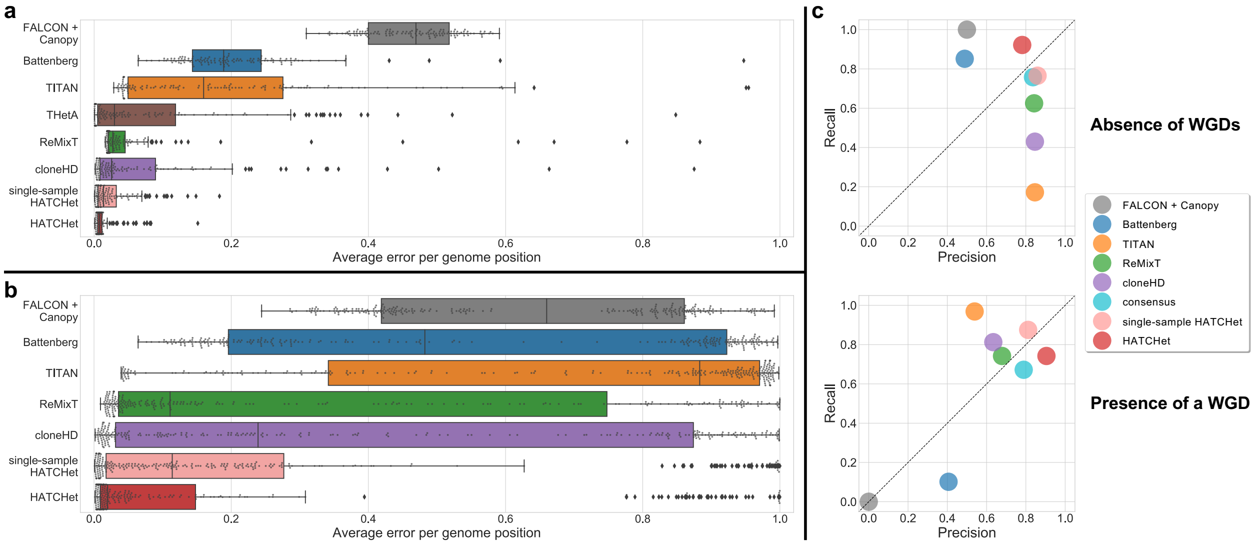

Supplementary Fig. 8: HATCHet outperforms existing methods in the inference of total copy numbers, their proportions, and WGDs. **a**, Average total copy-number error per genome position for the total copy numbers and their proportions inferred by each method (here excluding THetA which does not infer allele-specific copy numbers) on 128 simulated tumor samples from 32 patients without a WGD, and where each method was provided with the true values of the main parameters (e.g. tumor ploidy, number of clones, and maximum copy number). **b**, Average total copy-number error per genome position on 256 simulated samples from 64 patients, half with a WGD, and where each method infers all relevant parameters including tumor ploidy, number of clones, etc. Box plots show the median and the interquartile range (IQR), and the whiskers denote the lowest and highest values within 1.5 times the IQR from the first and third quartiles, respectively. **c**, Average precision and recall in the prediction of the absence of a WGD and the presence of a WGD in a sample. HATCHet is the only method with high precision and recall (>75%) in both the cases, even compared to a consensus of the other methods based on a prediction for majority. While Battenberg and Canopy underestimate the presence of WGDs (<20% and 0% recall), TITAN, ReMixT, and cloneHD overestimates the absence of WGDs (<20%, <62%, and <50% recall, respectively).

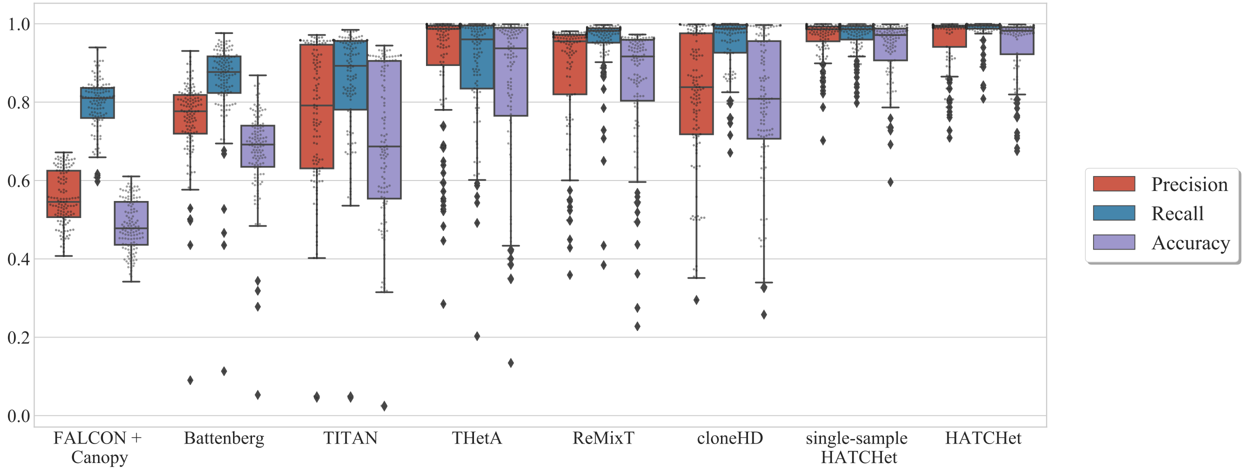

Supplementary Fig. 9: Precision and recall of total copy numbers with fixed parameters. The precision, recall, and accuracy of total copy numbers are computed when running eight methods on the 128 simulated samples without a WGD by providing the true value of the main parameters, e.g. tumor ploidy, number of clones, and maximum copy number. The considered methods are six current state-of-the-art methods (Battenberg, TITAN, THetA, cloneHD, Canopy with FALCON, and ReMixT) and HATCHet, which has been applied both separately on single samples (single-sample HATCHet) and jointly on multiple samples from the same patient (HATCHet). The precision and recall of total copy numbers are computed on each sample as the average per genome position and accuracy combines these two measures. Box plots show the median and the interquartile range (IQR), and the whiskers denote the lowest and highest values within 1.5 times the IQR from the first and third quartiles, respectively.

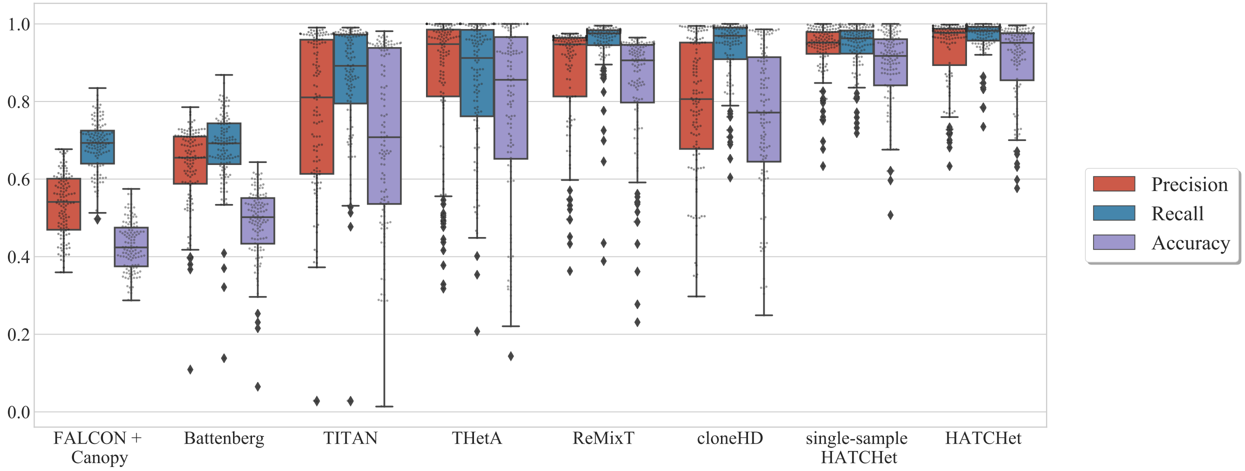

Supplementary Fig. 10: Precision and recall of total copy numbers per segment of at least 50kb size with fixed parameters. The precision, recall, and accuracy of total copy numbers per segment of at least 50kb size are computed when running eight methods on the 128 simulated samples without a WGD by providing the true value of the main parameters, e.g. tumor ploidy, number of clones, and maximum copy number. The considered methods are six current state-of-the-art methods (Battenberg, TITAN, THetA, cloneHD, Canopy with FALCON, and ReMixT) and HATCHet, which has been applied both separately on single samples (single-sample HATCHet) and jointly on multiple samples from the same patient (HATCHet). The precision and recall of total copy numbers are computed on each sample as the average per segment containing at least 50k genomic positions and accuracy combines these two measures. Box plots show the median and the interquartile range (IQR), and the whiskers denote the lowest and highest values within 1.5 times the IQR from the first and third quartiles, respectively.

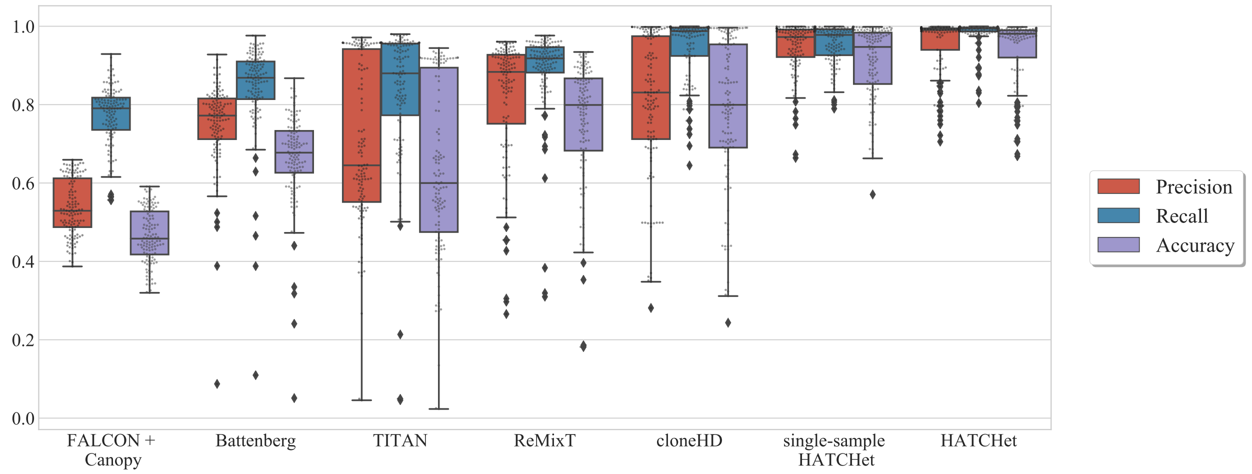

Supplementary Fig. 11: Precision and recall of copy-number states with fixed parameters. The precision, recall, and accuracy of copy-number states are computed when running eight methods on the 128 simulated samples without a WGD by providing the true value of the main parameters, e.g. tumor ploidy, number of clones, and maximum copy number. The considered methods are six current state-of-the-art methods (Battenberg, TITAN, THetA, cloneHD, Canopy with FALCON, and ReMixT) and HATCHet, which has been applied both separately on single samples (single-sample HATCHet) and jointly on multiple samples from the same patient (HATCHet). The precision and recall of copy-number states are computed on each sample as the average per genome position and accuracy combines these two measures. Box plots show the median and the interquartile range (IQR), and the whiskers denote the lowest and highest values within 1.5 times the IQR from the first and third quartiles, respectively.

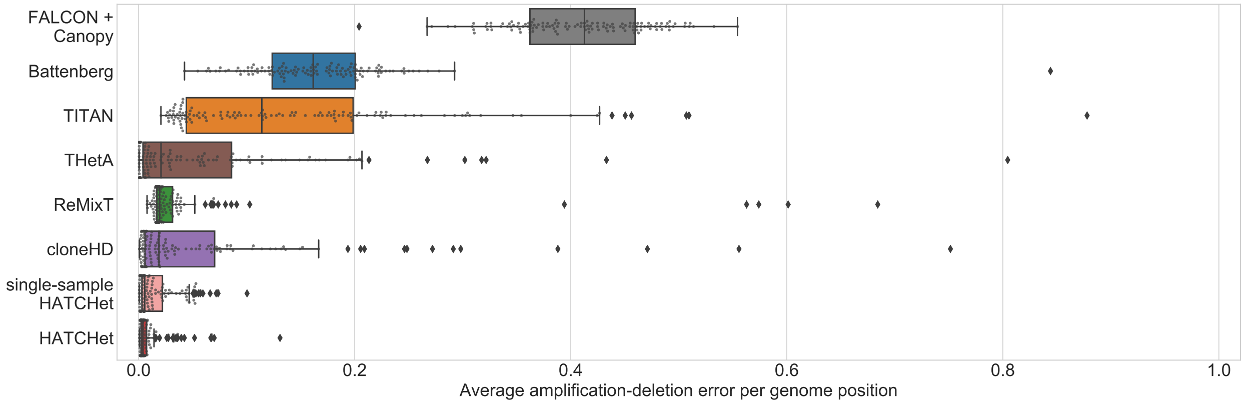

Supplementary Fig. 12: Average amplification-deletion error per genome position with fixed parameters. The average amplification-deletion error per genome position is computed when running eight methods on the 128 simulated samples without a WGD by providing the true value of the main parameters, e.g. tumor ploidy, number of clones, and maximum copy number. The considered methods are six current state-of-the-art methods (Battenberg, TITAN, THetA, cloneHD, Canopy with FALCON, and ReMixT) and HATCHet, which has been applied both separately on single samples (single-sample HATCHet) and jointly on multiple samples from the same patient (HATCHet). Box plots show the median and the interquartile range (IQR), and the whiskers denote the lowest and highest values within 1.5 times the IQR from the first and third quartiles, respectively. The average amplification-deletion error per genome position is computed for each sample and assesses whether the total copy-number of a genome position has been correctly identified as either amplified or deleted or unaltered relatively to allele-specific copy number θ of the base state that is $\theta = 1$ without a WGD and $\theta = 2$ with a WGD. The error is hence independent from the correct prediction of a WGD.

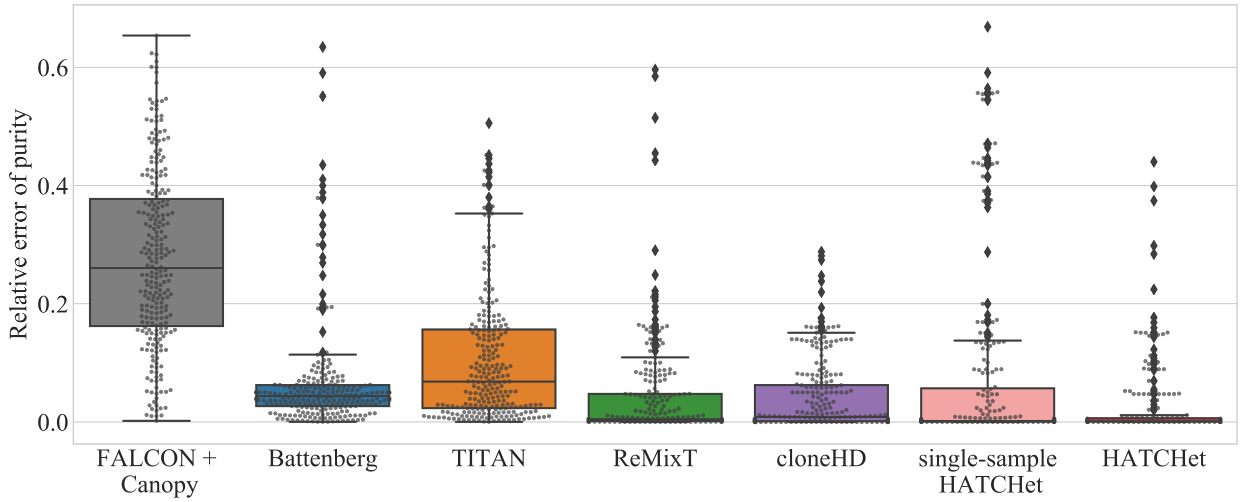

Supplementary Fig. 13: Relative error of tumor purity. The relative error of tumor purity is computed when running seven methods on all the 256 simulated samples, 128 without a WGD and 128 with a WGD, by considering free values of all parameters. The considered methods are five current state-of-the-art methods (Battenberg, TITAN, cloneHD, Canopy with FALCON, and ReMixT) and HATCHet, which has been applied both separately on single samples (single-sample HATCHet) and jointly on multiple samples from the same patient (HATCHet). Box plots show the median and the interquartile range (IQR), and the whiskers denote the lowest and highest values within 1.5 times the IQR from the first and third quartiles, respectively.

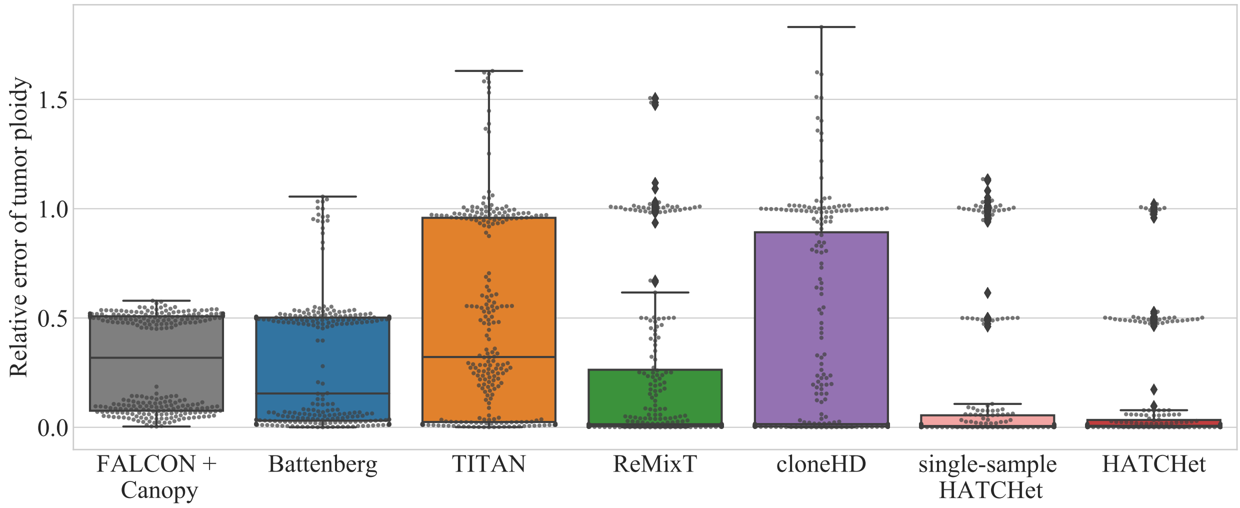

Supplementary Fig. 14: Relative error of tumor ploidy. The relative error of tumor ploidy is computed when running seven methods on all the 256 simulated samples, 128 without a WGD and 128 with a WGD, by considering free values of all parameters. The considered methods are five current state-of-the-art methods (Battenberg, TITAN, cloneHD, Canopy with FALCON, and ReMixT) and HATCHet, which has been applied both separately on single samples (single-sample HATCHet) and jointly on multiple samples from the same patient (HATCHet). Box plots show the median and the interquartile range (IQR), and the whiskers denote the lowest and highest values within 1.5 times the IQR from the first and third quartiles, respectively.

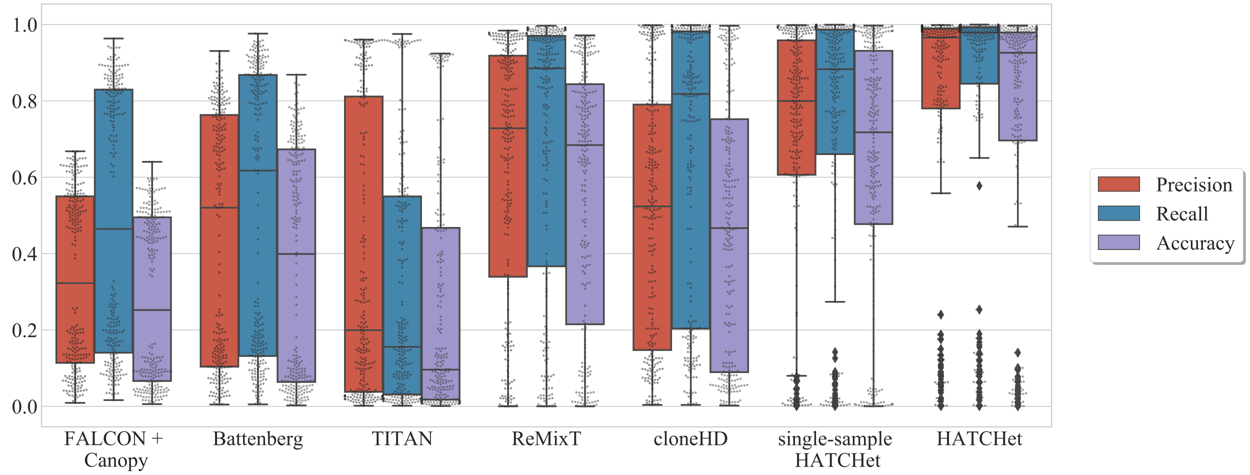

Supplementary Fig. 15: Precision and recall of total copy numbers with free parameters. The precision, recall, and accuracy of total copy numbers are computed when running seven methods on all the 256 simulated samples, 128 without a WGD and 128 with a WGD, by considering free values of all parameters. The considered methods are five current state-of-the-art methods (Battenberg, TITAN, cloneHD, Canopy with FALCON, and ReMixT) and HATCHet, which has been applied both separately on single samples (single-sample HATCHet) and jointly on multiple samples from the same patient (HATCHet). The precision and recall of total copy numbers are computed on each sample as the average per genome position and accuracy combines these two measures. Box plots show the median and the interquartile range (IQR), and the whiskers denote the lowest and highest values within 1.5 times the IQR from the first and third quartiles, respectively.

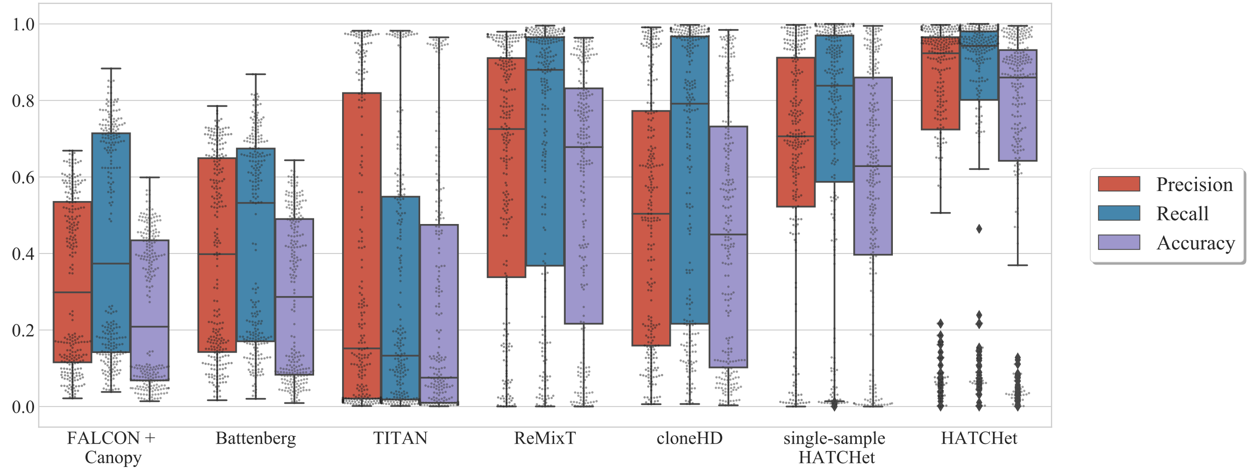

Supplementary Fig. 16: Precision and recall of total copy numbers per segment of at least 50kb size with free parameters. The precision, recall, and accuracy of total copy numbers per segment of at least 50kb size are computed when running seven methods on all the 256 simulated samples, 128 without a WGD and 128 with a WGD, by considering free values of all parameters. The considered methods are five current state-of-the-art methods (Battenberg, TITAN, cloneHD, Canopy with FALCON, and ReMixT) and HATCHet, which has been applied both separately on single samples (single-sample HATCHet) and jointly on multiple samples from the same patient (HATCHet). The precision and recall of total copy numbers are computed on each sample as the average per segment containing at least 50k genomic positions and accuracy combines these two measures. Box plots show the median and the interquartile range (IQR), and the whiskers denote the lowest and highest values within 1.5 times the IQR from the first and third quartiles, respectively.

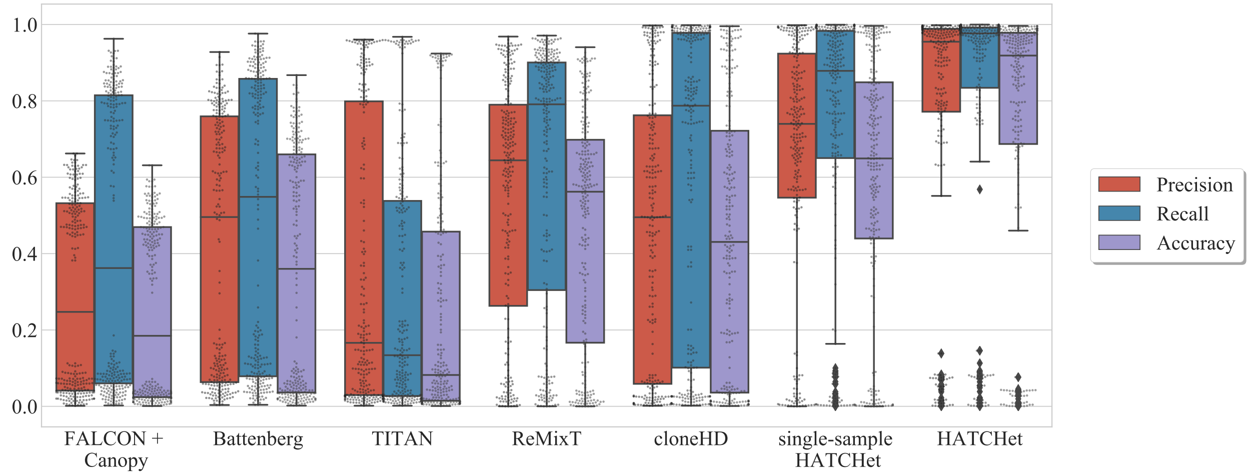

Supplementary Fig. 17: Precision and recall of copy-number states with free parameters. The precision, recall, and accuracy of copy-number states are computed when running seven methods on all the 256 simulated samples, 128 without a WGD and 128 with a WGD, by considering free values of all parameters. The considered methods are five current state-of-the-art methods (Battenberg, TITAN, cloneHD, Canopy with FALCON, and ReMixT) and HATCHet, which has been applied both separately on single samples (single-sample HATCHet) and jointly on multiple samples from the same patient (HATCHet). The precision and recall of copy-number states are computed on each sample as the average per genome position and accuracy combines these two measures. Box plots show the median and the interquartile range (IQR), and the whiskers denote the lowest and highest values within 1.5 times the IQR from the first and third quartiles, respectively.

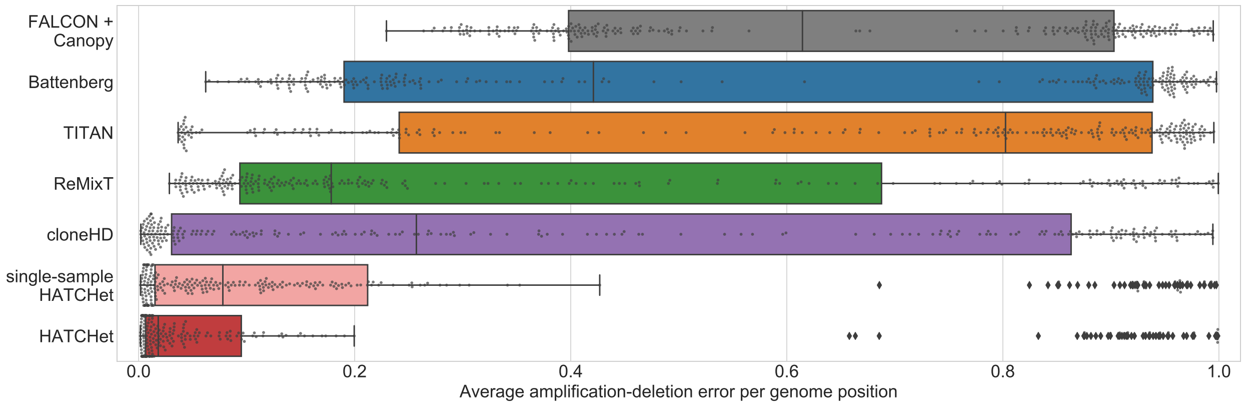

Supplementary Fig. 18: Average amplification-deletion error per genome position with free parameters. The average amplification-deletion error per genome position is computed when running seven methods on all the 256 simulated samples, 128 without a WGD and 128 with a WGD, by considering free values of all parameters. The considered methods are five current state-of-the-art methods (Battenberg, TITAN, cloneHD, Canopy with FALCON, and ReMixT) and HATCHet, which has been applied both separately on single samples (single-sample HATCHet) and jointly on multiple samples from the same patient (HATCHet). Box plots show the median and the interquartile range (IQR), and the whiskers denote the lowest and highest values within 1.5 times the IQR from the first and third quartiles, respectively. The average amplification-deletion error per genome position is computed for each sample and assesses whether the total copy-number of a genome position has been correctly identified as either amplified or deleted or unaffected, with the unaffected status which is defined as a total copy number equal to either 2 or 4 without or with a WGD, respectively. The error is hence independent from the correct prediction of a WGD; a method that does not correctly identify the exact copy numbers because it wrongly predicts the presence/absence of a WGD can still obtain a low amplification-deletion error by correctly inferring the amplified-deleted segments. The high amplification-deletion error obtained by the current methods for nearly all the samples indicates poor predictions of amplified-deleted segments.

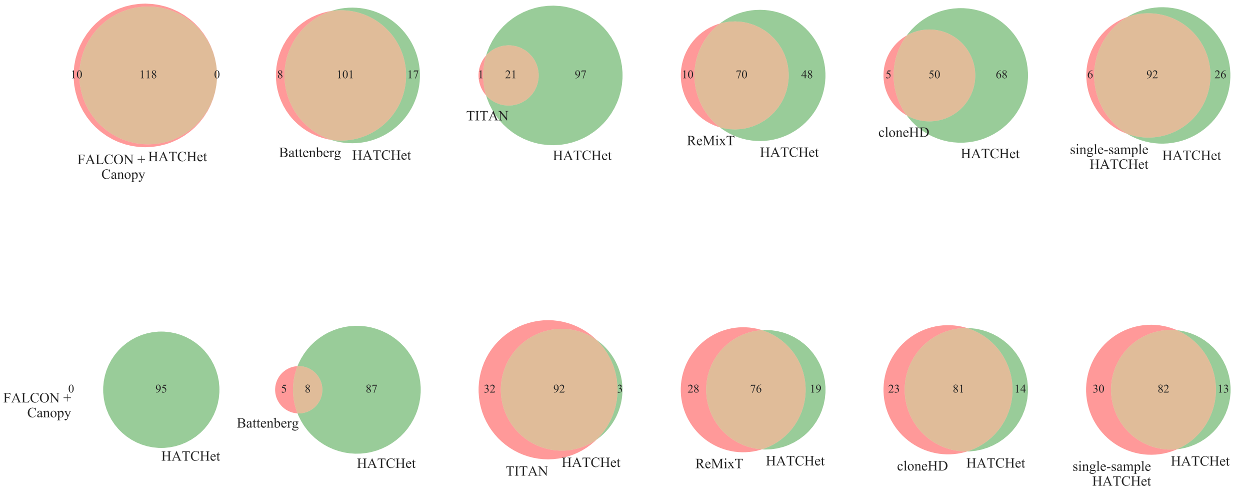

Supplementary Fig. 19: Consistency in WGD predictions on simulated data among different methods. (Top) A Venn diagram represents the number of samples simulated without a WGD that have been correctly predicted without a WGD by both HATCHet and six other methods: five current state-of-the-art methods (Battenberg, TITAN, cloneHD, Canopy with FALCON, and ReMixT) and HATCHet applied on single samples (single-sample HATCHet). (Bottom) A Venn diagram represents the number of samples simulated with a WGD that have been correctly predicted with a WGD by both HATCHet and six other methods: five current state-of-the-art methods (Battenberg, TITAN, cloneHD, Canopy with FALCON, and ReMixT) and HATCHet applied on single samples (single-sample HATCHet).

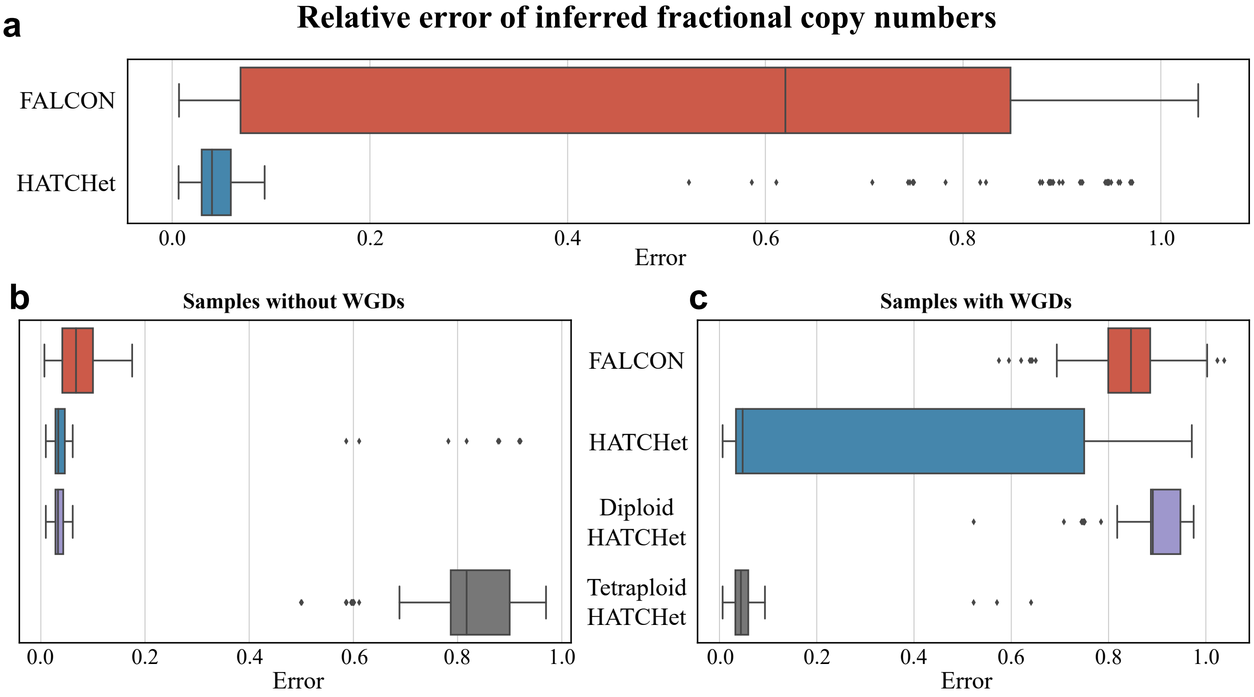

Supplementary Fig. 20: HATCHet outperforms FALCON in deriving fractional copy numbers. **a**, Relative error between the true and estimated fractional copy numbers on 256 simulated samples from 64 patients, 32 without a WGD and 32 with a WGD. HATCHet operates by evaluating solutions without a WGD (Diploid HATCHet) and solutions with a WGD (Tetraploid HATCHet), and selecting among solutions using a novel model-selection criterion. The results labeled HATCHet are the output following this model selection. **b**, Restricting to the 128 samples from the 32 patients without a WGD, one sees that the Diploid-HATCHet solutions always have lower error. HATCHet correctly chooses the diploid solution in nearly all cases, and also outperforms FALCON. **c**, Restricting to the 128 samples of the 32 patients with a WGD, one sees that Tetraploid-HATCHet solutions always have lowest error. HATCHet correctly chooses the solution with a WGD in most of the samples. In contrast, FALCON computes fractional copy numbers with a very high error for nearly all samples. Box plots show the median and the interquartile range (IQR), and the whiskers denote the lowest and highest values within 1.5 times the IQR from the first and third quartiles, respectively.

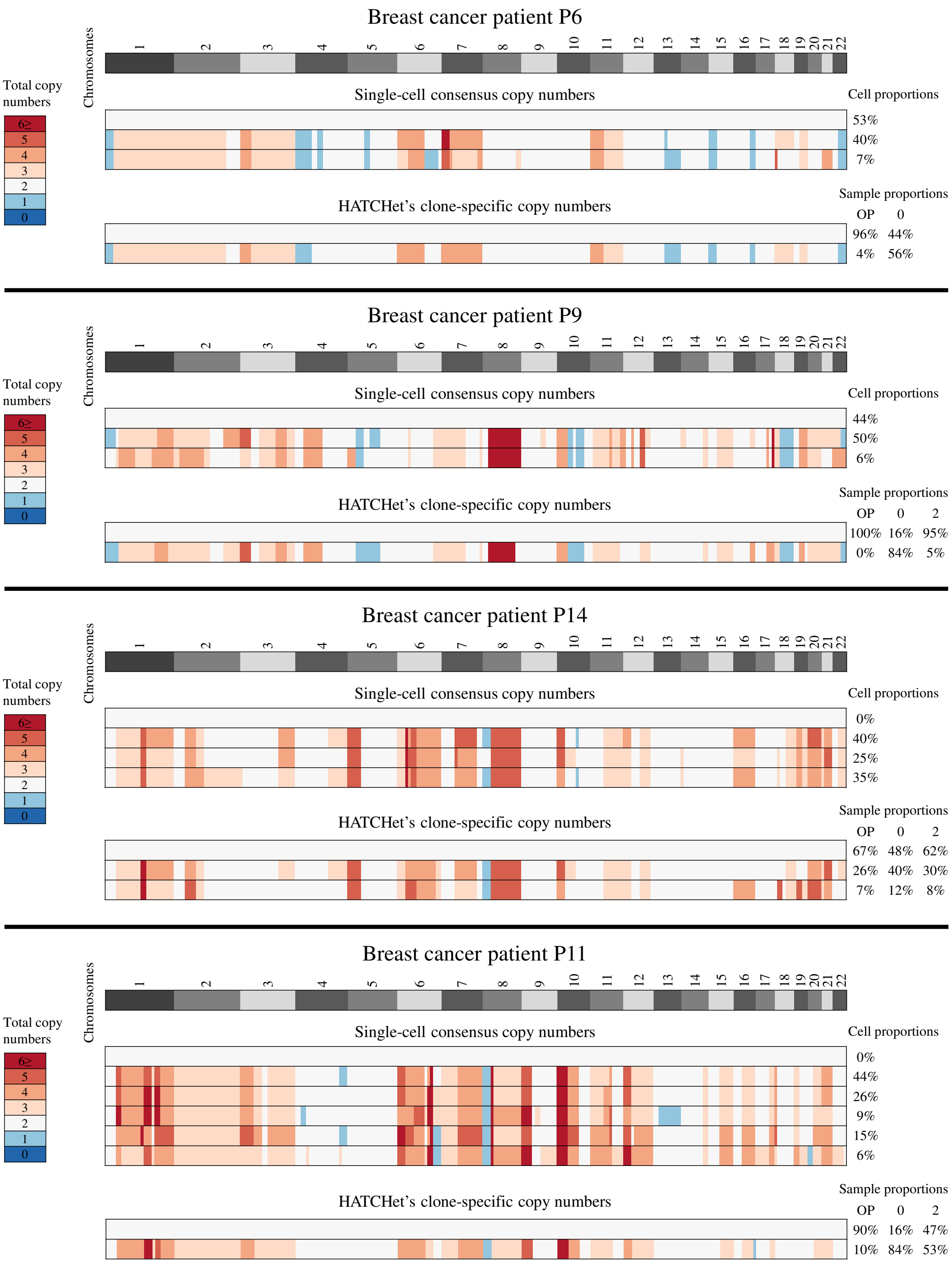

Supplementary Fig. 21: HATCHet's copy numbers are consistent with published single-cell copy-number profiles of 4 breast cancer patients. (Top of each panel) Single-cell copy-number profiles for each clone identified in DOP-PCR single-cell sequencing data from 4 breast cancer patients in Kim et al.[1]. On the right are the proportion of cells assigned to each clone. (Bottom of each panel) Total copy numbers inferred by HATCHet using whole-exome sequencing data from 2-3 bulk tumor samples (OP, 0, and 2) from each patient. On the right are the clone proportions inferred by HATCHet.

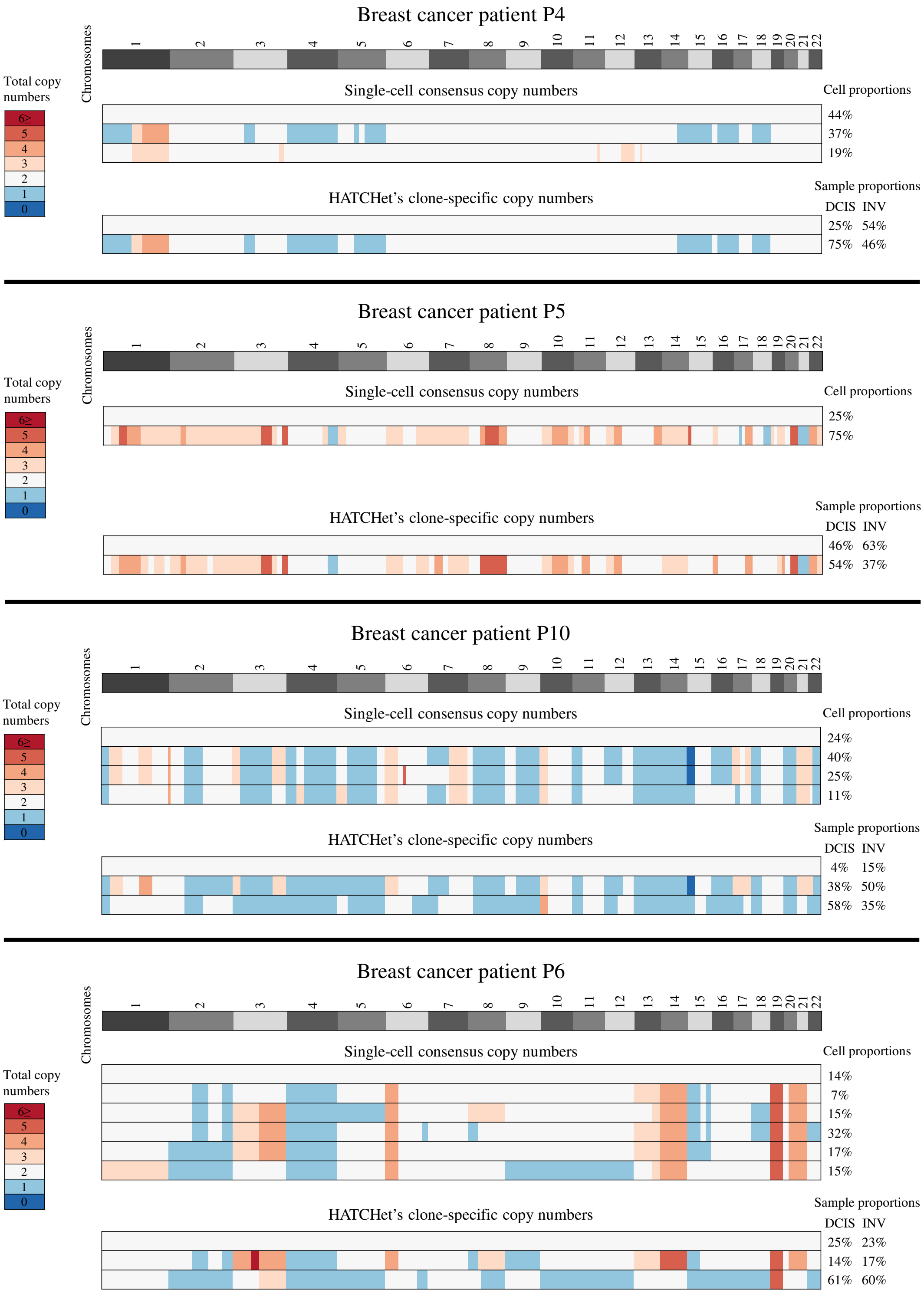

Supplementary Fig. 22: HATCHet's copy numbers are consistent with published single-cell copy-number profiles of 4 breast cancer patients. (Top of each panel) Single-cell copy-number profiles for each clone identified in DOP-PCR single-cell sequencing data from 4 breast cancer patients in Casasent et al.[2]. On the right are the proportion of cells assigned to each clone. (Bottom of each panel) Total copy numbers inferred by HATCHet using whole-exome sequencing data from 2 bulk tumor samples (DCIS and INV) from each patient. On the right are the clone proportions inferred by HATCHet.

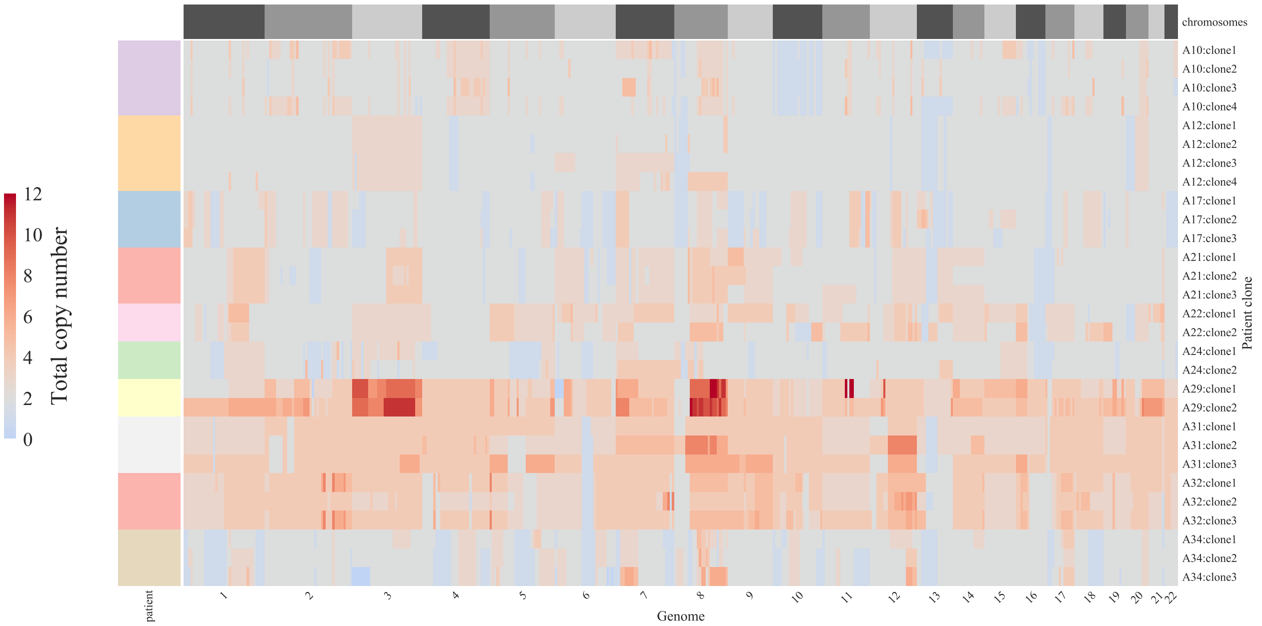

Supplementary Fig. 23: Total copy-number profiles of tumor clones in prostate cancer patients. Columns correspond to 5Mb genomic regions, which are sorted according to their position in the genome and partitioned into the corresponding chromosomes (alternating gray-scale boxes in the first row with names reported at the bottom). Each row corresponds to a tumor clone inferred from the samples of a prostate cancer patient, represented in the first column with different colors. Each cell of the grid is colored according to the most common total copy number inferred by HATCHet in the corresponding genomic region for the corresponding clone. A grid cell colored in gray represents a total copy number equal to 2. While most of the patients have the majority of the grid cells colored in gray as they are not affected by CNAs and are diploid, patients A29, A31, and A32 have most of the cells in light red as they are affected by WGD and have a total copy number equal to 4.

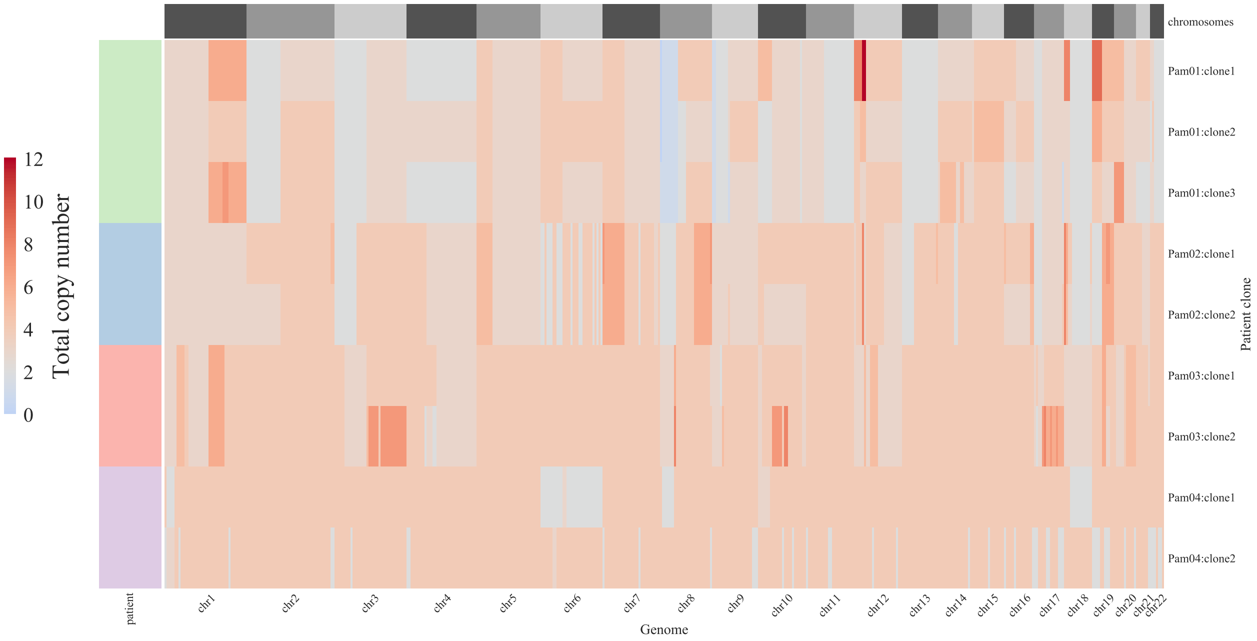

Supplementary Fig. 24: Total copy-number profiles of tumor clones in pancreas cancer patients. Columns correspond to 5Mb genomic regions, which are sorted according their position in the genome and partitioned into the corresponding chromosomes (alternating gray-scale boxes in the first row with names reported at the bottom). Each row corresponds to a tumor clone inferred from the samples of a pancreas cancer patient patient, represented in the first column with different colors. Each cell of the grid is colored according to the most common total copy number inferred by HATCHet in the corresponding genomic region for the corresponding clone. Most of the grid cells are colored in light red – indicating a total copy number higher or equal to 4 – as all patients are affected by either WGD (Pam02, Pam03, and Pam04) or massive rearrangements (Pam01).

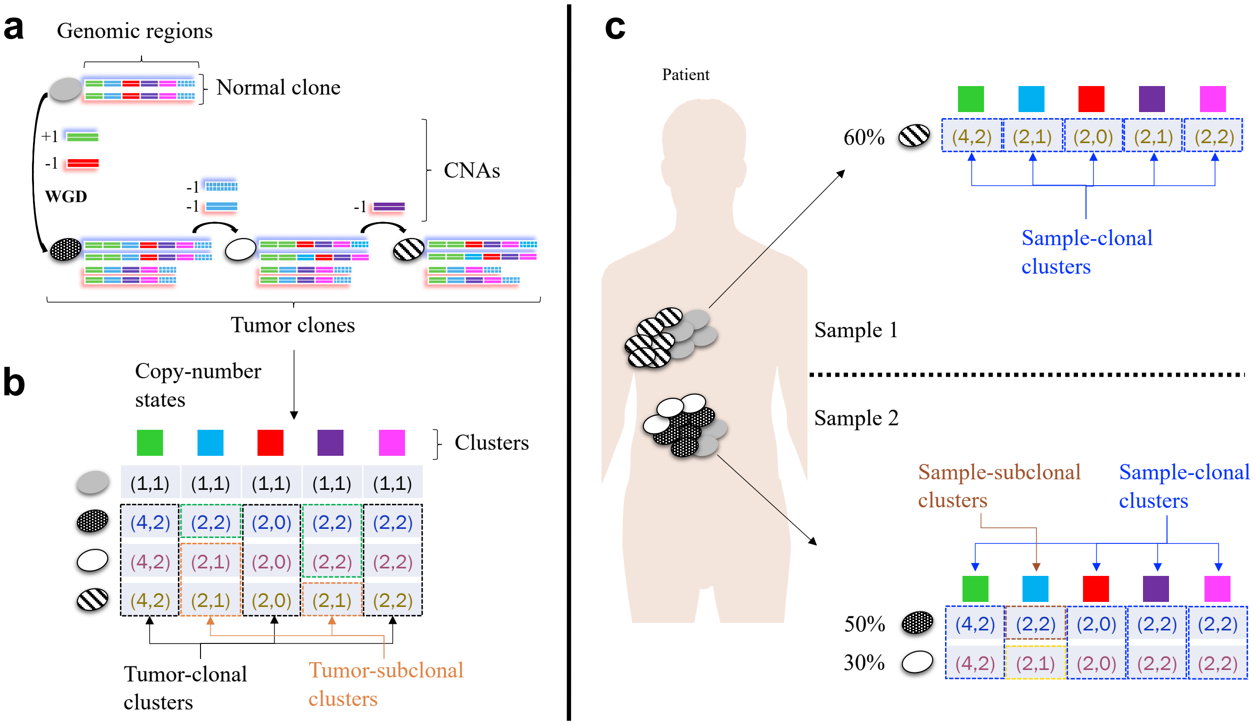

Supplementary Fig. 25: Classification of sample- or tumor-specific clonal and subclonal clusters. **a**, The genome of a normal clone (gray ellipse) is described by 6 segments (rectangles of different colors and textures), each having 2 alleles. In an evolutionary process, 3 tumor clones (ellipses of different textures) accumulate a WGD and different CNAs, 1 amplification and 4 deletions in total. **b**, For each segment s, the copy-number state $(a_{s,i}, b_{s,i})$ models the allele-specific copy numbers $a_{s,i}$ and $b_{s,i}$ of each clone i. A cluster is classified as tumor-clonal when all tumor clones have the same copy-number state in that cluster (black boxes), otherwise the cluster is tumor-subclonal (green and orange boxes). **c**, Two samples correspond to different mixtures of the normal and tumor clones. As such, each sample contains only a subset of all clones in the tumor. A cluster is hence classified specifically to each sample p such that a cluster is sample-clonal in p if all the tumor clones in p have the same copy-number state in that cluster (blue boxes), otherwise is sample-subclonal in p.

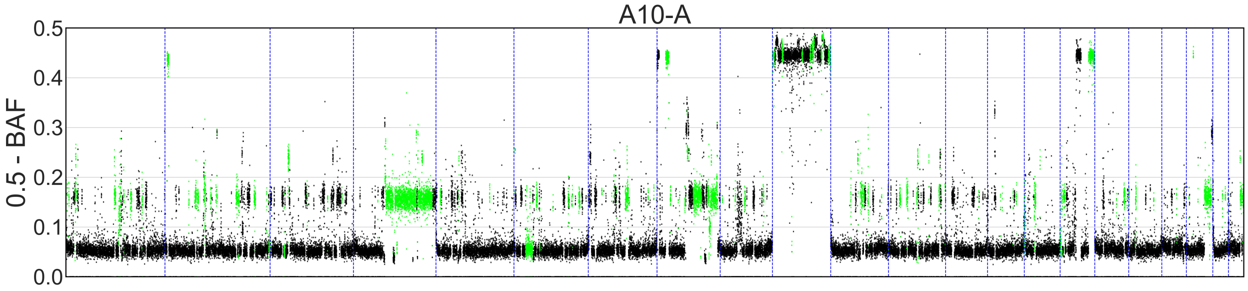

Supplementary Fig. 26: Battenberg identifies subclonal genomic regions which are not supported by measured data in sample A10-A. In sample A10-A of patient A10, Battenberg identifies large genomic regions from sample-subclonal clusters (green) with values of BAF (for 50kb genomic bins) approximately equal to the BAF values of nearby regions from sample-clonal clusters (black).

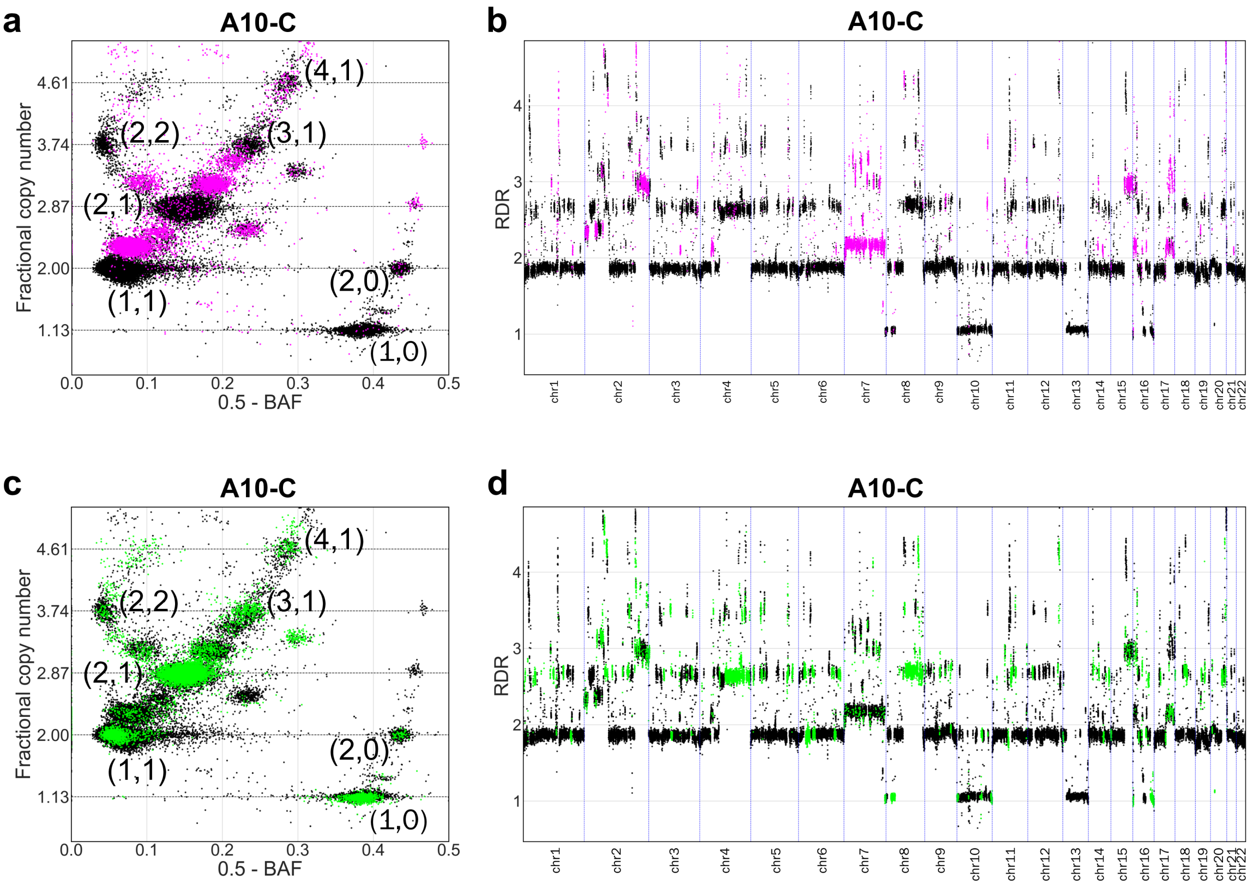

Supplementary Fig. 27: Battenberg overestimates the presence of subclonal CNAs in the sample A10-C of prostate cancer patient A10. **a**, In sample A10-C of patient A10, both HATCHet and Battenberg identify reliable subclonal CNAs that correspond to sample-subclonal clusters (magenta) with positions in the scaled BAF-RDR plot (each point corresponds to 50kb genomic bin) that are clearly in between the positions of sample-clonal clusters (black clusters with corresponding copy-number states). **b**, The sample-subclonal clusters in (**a**) correspond to large genomic regions (magenta) with values of RDR (for 50kb genomic bins) that are clearly distinct from the RDR values of regions from sample-clonal clusters (black). **c**, In the same sample A10-C, Battenberg identifies extensive clusters of genomic bins with subclonal CNAs (green). However, the clusters corresponding to these subclonal CNAs are not clearly distinguished in the scaled BAF-RDR plot (each point corresponds to 50kb genomic bins) from sample-clonal clusters (black). Thus, HATCHet only identifies clonal CNAs in this sample. **d**, The sample-subclonal clusters in (**c**) correspond to large genomic regions (green) with values of RDR (for 50kb genomic bins) approximately equal to the RDR values of nearby regions from sample-clonal clusters (black).

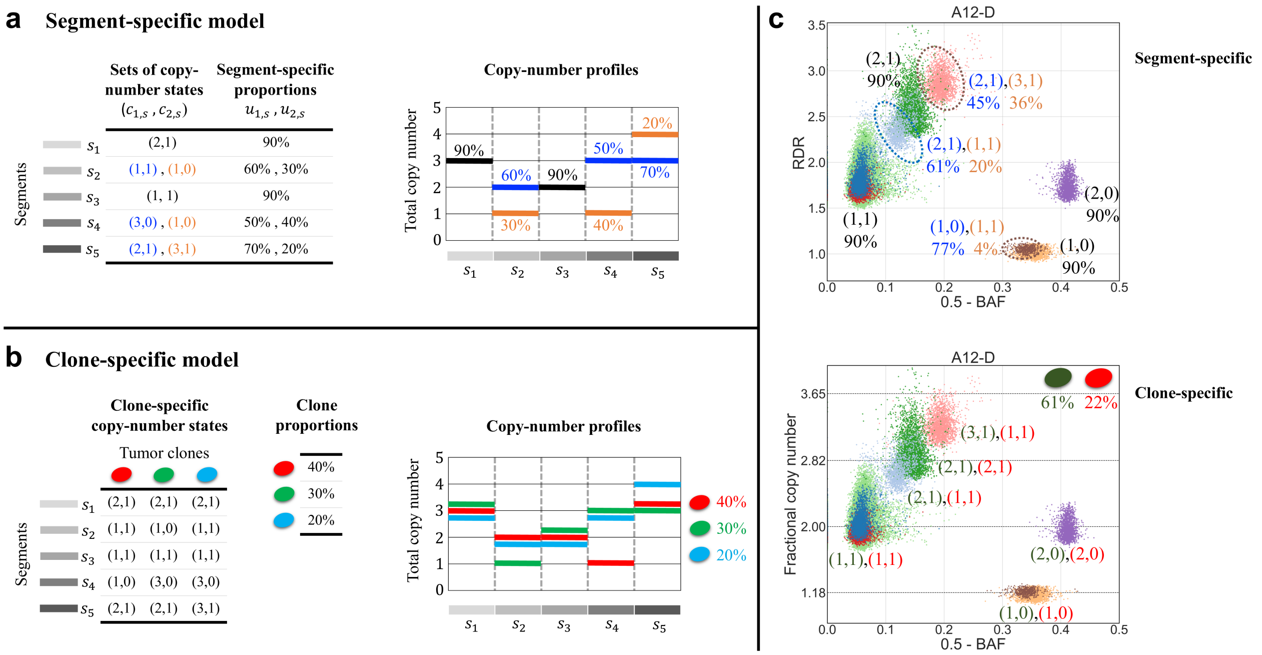

Supplementary Fig. 28: Segment-specific and clone-specific models of CNAs. **a**, Segment-specific models do not group CNAs into clones and consider each segment independently. Each segment (gray-scale rectangles) is thus modeled by a set of distinct copy-number states that the corresponding segment has in the tumor cells (black when there is a unique state, blue and orange when there are two different states). The proportion of tumor cells associated to each copy-number state (in the same order as the corresponding states) is defined for each segment independently. **b**, Clone-specific models group CNAs into clones and consider the proportion of each clone. Each clone is modeled by a clone proportion and a vector of copy-number states for each segment (each color represents a different clone). There is a global dependency among all segments since clone proportions are globally shared by all segments. **c**, In sample A12-D of prostate-cancer patient A12, the clusters of 50kb genomic bins with different copy-number states are inferred by either (Top) Battenberg, which is based on a segment-specific model (segment-specific copy-number states and proportions are correspondingly reported), or (Bottom) by HATCHet, which is based on a segment-specific model (clone-specific copy-number states and proportions are colored based on different clones).

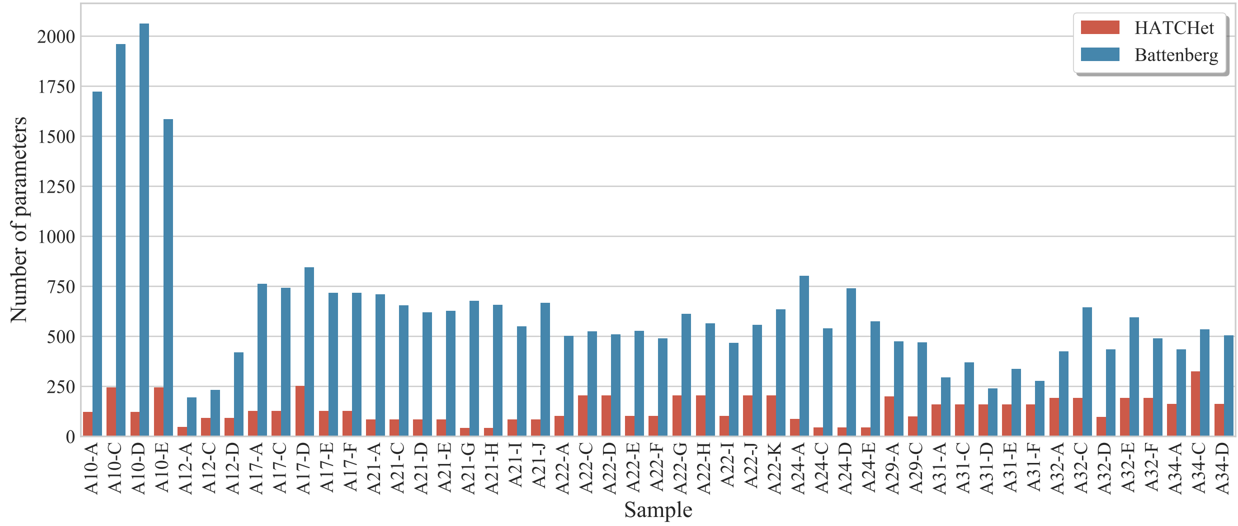

a

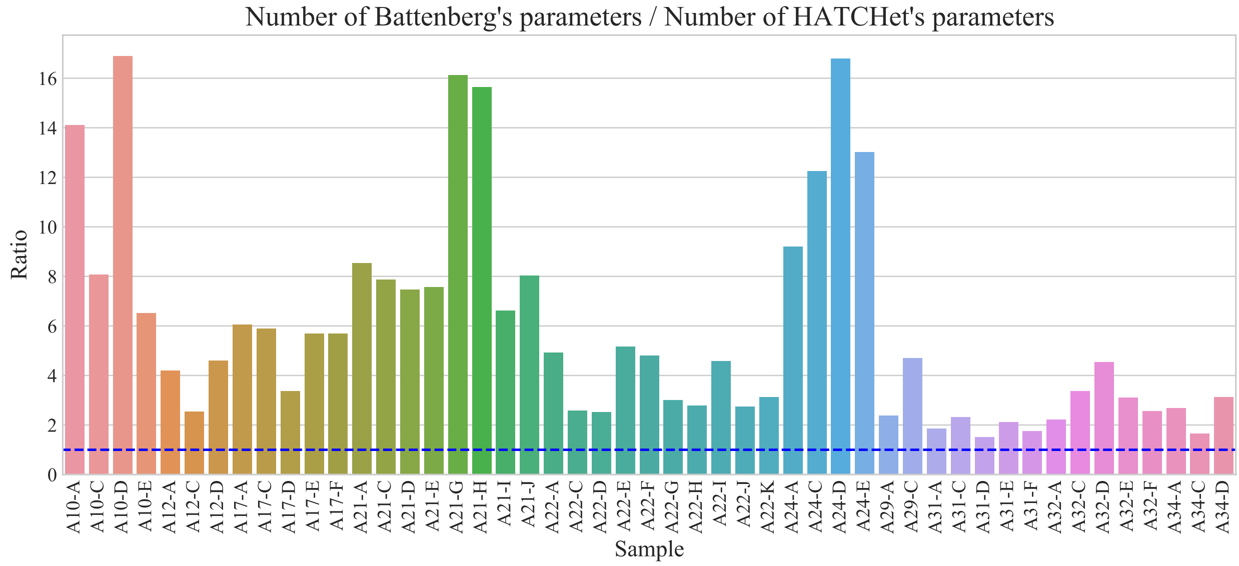

b

Supplementary Fig. 29: HATCHet uses six times less parameters than Battenberg on average across all solutions of the prostate cancer dataset. **a**, The number of parameters used by HATCHet and Battenberg is computed for the solution provided for every sample of the prostate cancer dataset. The number of parameters used by HATCHet for every sample corresponds to the number of allele-specific copy numbers for all clusters and the number of clone proportions. The number of parameters used by Battenberg for every sample corresponds to the number of allele-specific copy numbers for every segment and the number of corresponding proportions that are specific for each segment. **b**, The ratio between the number of parameters used by Battenberg and the number of parameter used by HATCHet is computed for all samples. Battenberg constantly uses more parameters than HATCHet across all samples (blue dashed line indicates where the ratio is equal to 1). More specifically, Battenberg uses on average at least 6 times more parameters than HATCHet.

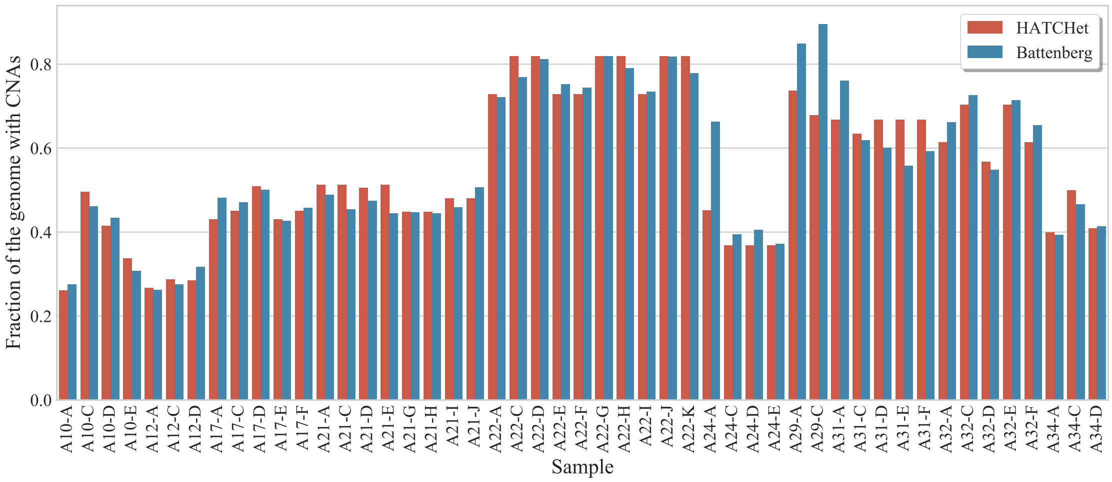

a

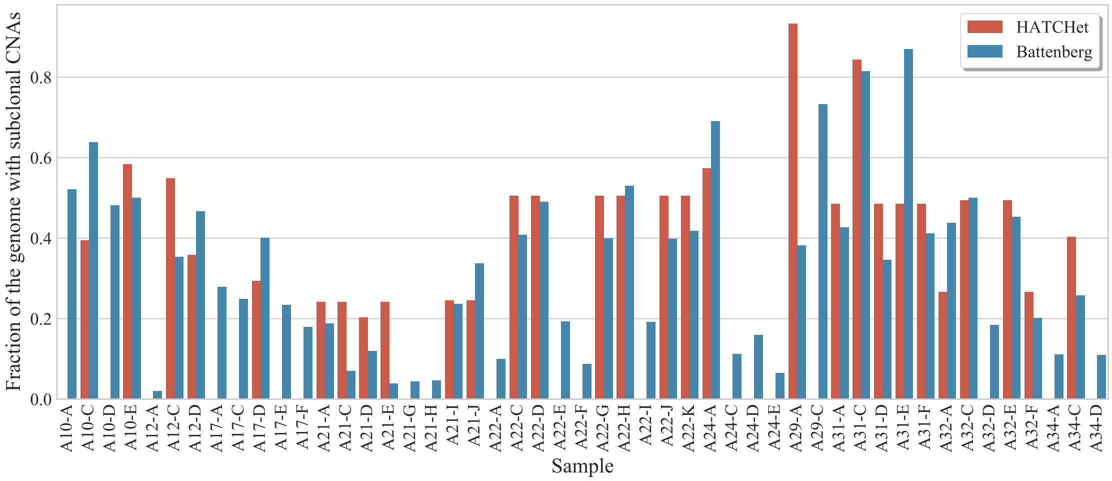

b

Supplementary Fig. 30: Fraction of the genome with CNAs and subclonal CNAs inferred by HATCHet and Battenberg for all samples of the prostate cancer dataset. **a**, The fraction of the genome with CNAs corresponds to the fraction of genomic positions with copy-number state different from the diploid state $(1, 1)$ when there is no WGD and $(2, 2)$ when there is a WGD. HATCHet and Battenberg infer a similar fraction of the genome with CNAs in most of the samples. **b**, The fraction of the genome with subclonal CNAs corresponds to the fraction of genomic positions that are included in sample-subclonal clusters relative to the fraction of the genome with CNAs. HATCHet and Battenberg infer a similar fraction of the genome with subclonal CNAs only in the 20 samples where HATCHet identifies multiple tumor clones. In the remaining samples, HATCHet explains as clonal CNAs the additional subclonal CNAs inferred by Battenberg.

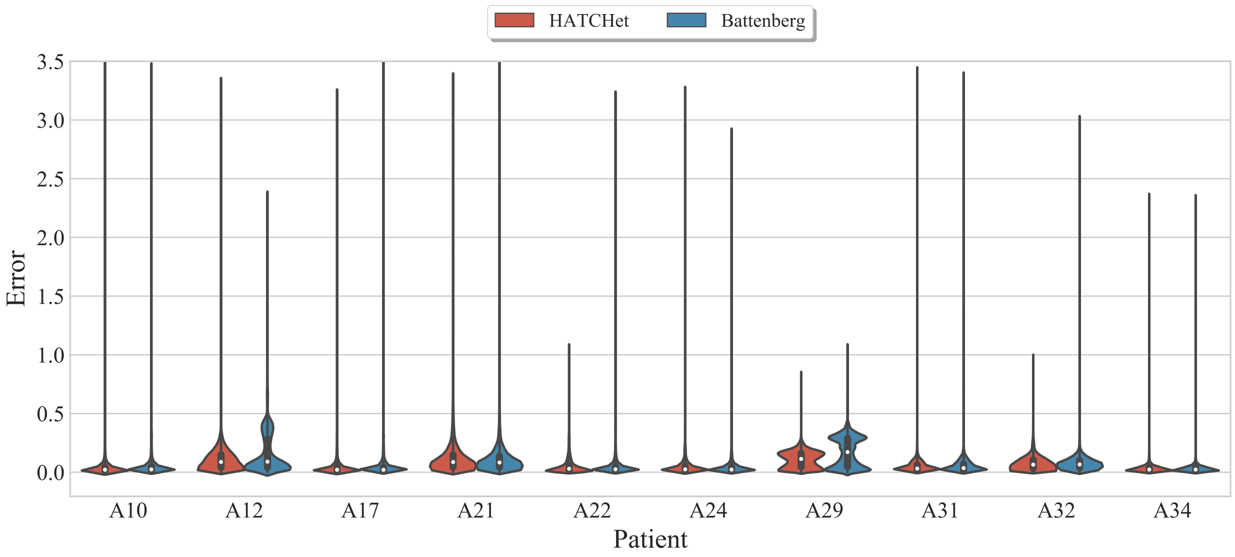

Supplementary Fig. 31: HATCHet achieves an error between the observed and estimated RDRs similar or lower than Battenberg. The error between the observed and estimated RDRs is defined as the absolute difference between these two values relative to the observed RDR. The error is computed for every bin of the reference genome in all samples of every patient of the prostate cancer dataset and the corresponding estimated RDRs are obtained when considering both the copy numbers and proportions inferred by HATCHet and Battenberg. Violin plots show a kernel density estimate of the error distributions with inner box plots showing the median (white dot) and the interquartile range (IQR), and the whiskers denote the lowest and highest values within 1.5 times the IQR from the first and third quartiles, respectively. HATCHet achieves the same average error of Battenberg for the samples of 8 patients (A10, A17, A21, A22, A24, A31, A32, and A32) and an average error lower than Battenberg for the samples of the remaining 2 patients (A12 and A29). Also, HATCHet decreases the highest error in the samples of 5 patients. Battenberg decreases the maximum errors only in two patients where the improvement is however limited (A24) or is achieved at the cost of a worse average error (A12).

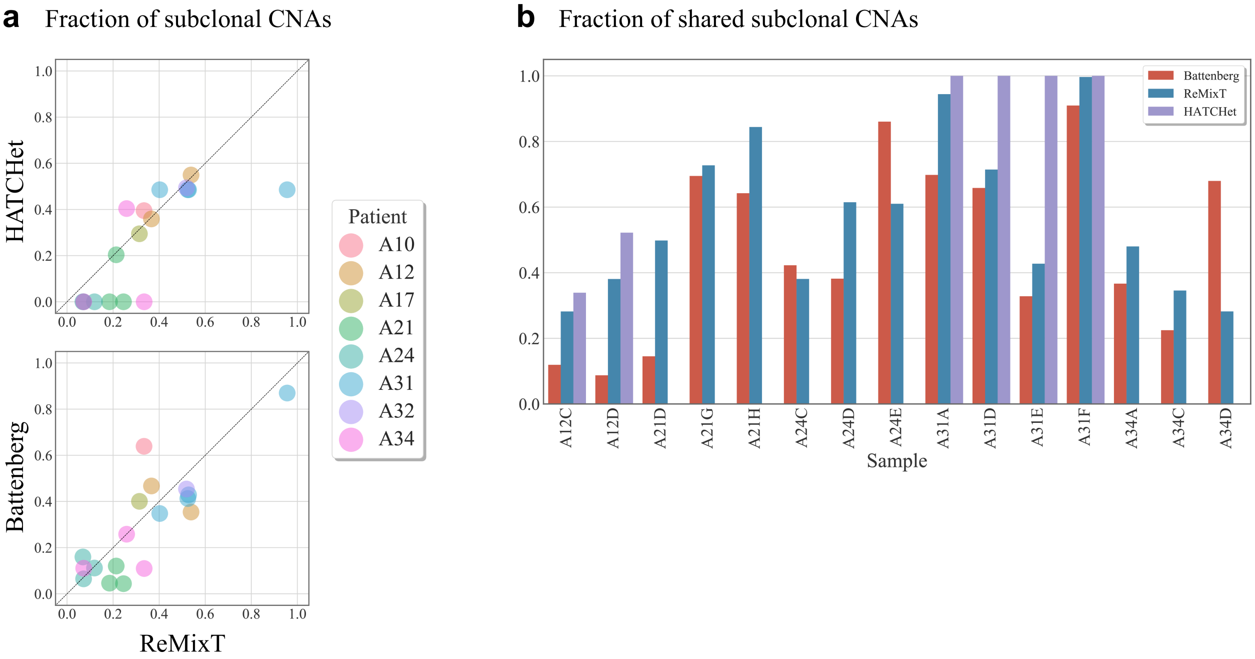

Supplementary Fig. 32: ReMixT and Battenberg infer extensive subclonal CNAs and shared subclonal CNAs in all samples of prostate cancer patients. **a**, The fraction of the genome with subclonal CNAs inferred by HATCHet, Battenberg, and ReMixT in 17 samples from 5 prostate cancer patients (A10, A12, A17, A31, and A32). HATCHet infers subclonal CNAs only in 10 samples, while ReMixT and Battenberg infer subclonal CNAs in all samples in high fractions of the genome. **b**, ReMixT and Battenberg infer high rates of shared subclonal CNAs in all 17 samples, with ReMixT reporting higher rates of shared subclonal CNAs than Battenberg in all but 3 samples. In contrast, HATCHet identifies shared subclonal CNAs in samples from only 2 patients (A12 and A31). Notably, ReMixT identifies a fraction of the genome with subclonal CNAs closer to HATCHet 's results (average difference of 18.6%) than Battenberg 's results (average difference of 34.3%) in samples where HATCHet identifies subclonal CNAs.

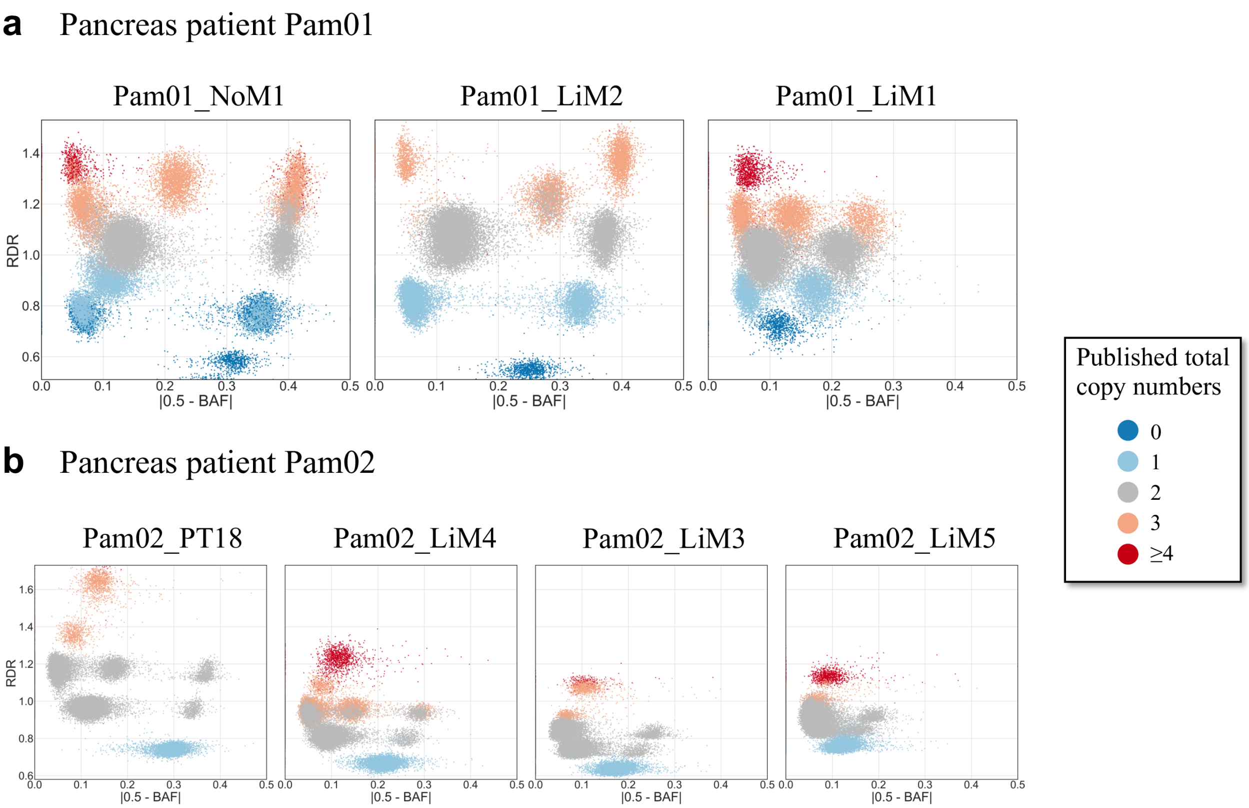

Supplementary Fig. 33: Published copy numbers derived from Control-FREEC for pancreas cancer patients Pam01 and Pam02 are inconsistent across samples and miss subclonal CNAs and WGDs. **a**, RDRs and BAFs of 50kb genomic bins in three samples from the pancreas cancer patient Pam01 are colored according to the published total copy numbers inferred by Control-FREEC. **b**, RDRs and BAFs of 50kb genomic bins in four samples from the pancreas cancer patient Pam02 are colored according to the published total copy numbers inferred by Control-FREEC.

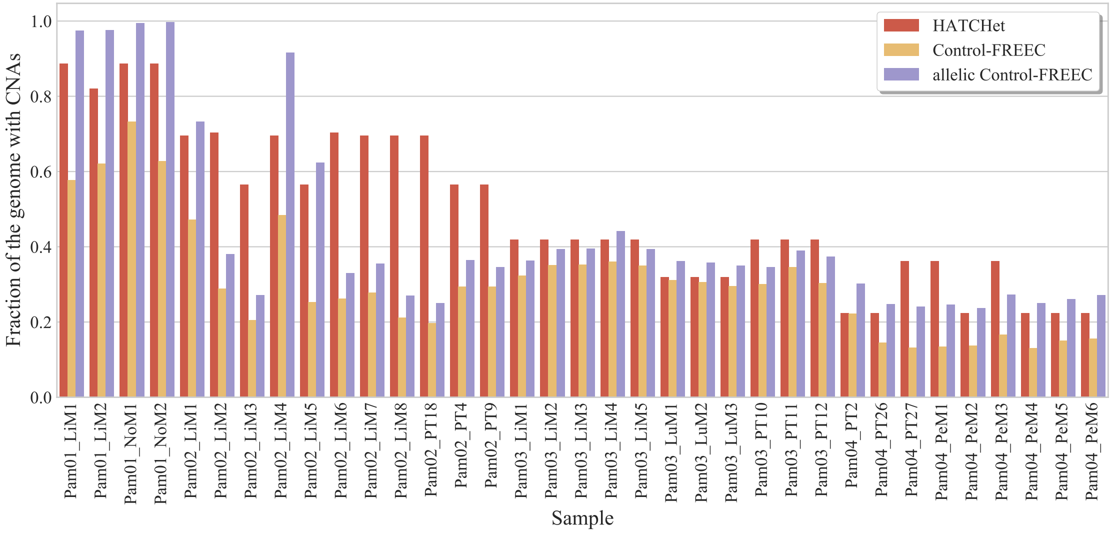

a

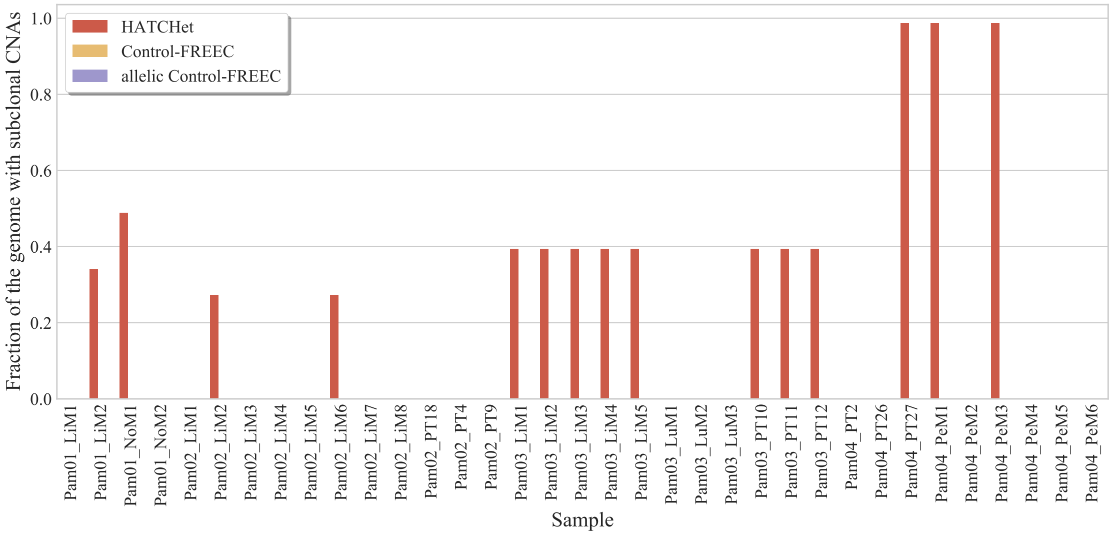

b

Supplementary Fig. 34: Fraction of the genome with CNAs and subclonal CNAs inferred by HATCHet and Control-FREEC for all samples of the pancreas cancer dataset. a, The fraction of the genome with CNAs corresponds to the fraction of genomic positions with copy-number state different from the diploid state $(1, 1)$ when there is no WGD and $(2, 2)$ when there is a WGD. As Control-FREEC only infers total copy numbers, we compute the fraction of the genome with CNAs both by identifying genomic regions with total copy number equal to 2θ (Control-FREEC) and by splitting the total copy numbers into the allele-specific copy numbers that better fit the observed BAFs (allelic Control-FREEC). HATCHet infers a higher fraction of CNAs than Control-FREEC on several samples of 3/4 patients (Pam02, Pam03, and Pam04) when both considering total and allele-specific copy numbers. **b**, The fraction of the genome with subclonal CNAs corresponds to the fraction of genomic positions that are included in sample-subclonal clusters relative to the fraction of the genome with CNAs. The fraction of the genome with subclonal CNAs is always equal to 0 for Control-FREEC as Control-FREEC assumes that every sample contains a single tumor clone. HATCHet identifies subclonal CNAs in most of the samples where Control-FREEC infers a fraction of the genome with CNAs lower than HATCHet, suggesting that Control-FREEC classifies as noisy diploid regions a high fraction of subclonal CNAs.

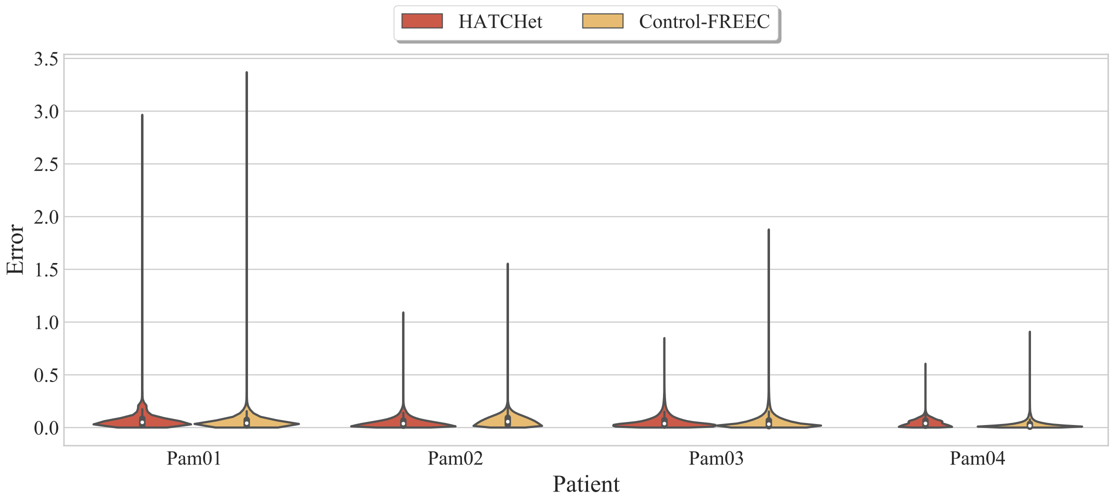

a

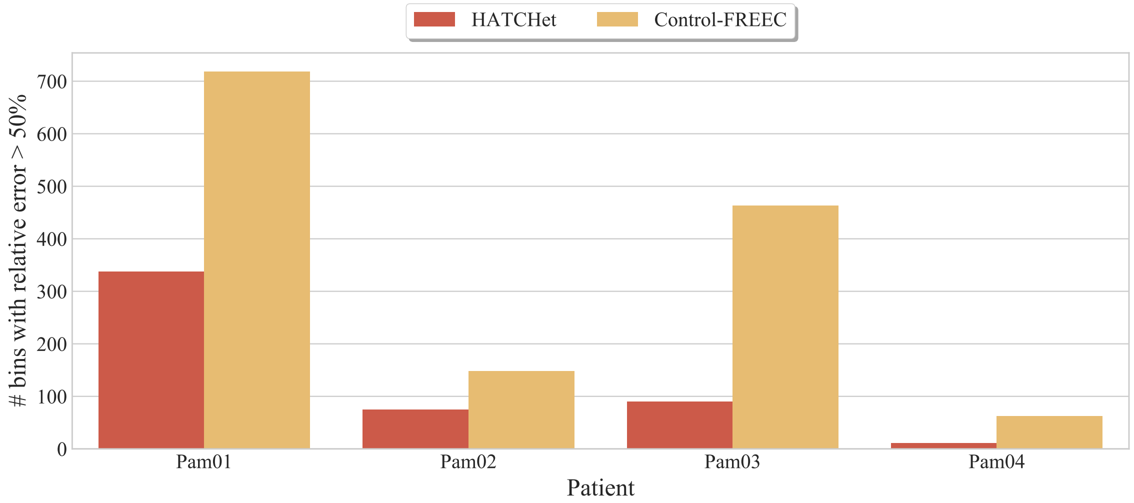

b

Supplementary Fig. 35: HATCHet has a lower error between the observed and estimated RDRs than Control-FREEC. a, The error between the observed and estimated RDRs is defined as the absolute difference between these two values relative to the observed RDR. The error is computed for every bin of the reference genome in all samples of every patient of the pancreas cancer dataset and the corresponding estimated RDRs are obtained when considering both the copy numbers and proportions inferred by HATCHet and Control-FREEC. Violin plots show a kernel density estimate of the error distributions with inner box plots showing the median (white dot) and the interquartile range (IQR), and the whiskers denote the lowest and highest values within 1.5 times the IQR from the first and third quartiles, respectively. HATCHet consistently achieves lower errors than Control-FREEC in the samples of all the patients. **b**, HATCHet consistently has a lower number of bins than Control-FREEC with a relative error higher than 0.5 across a total of 52 000 genomic bins of 50kb on average per patient.

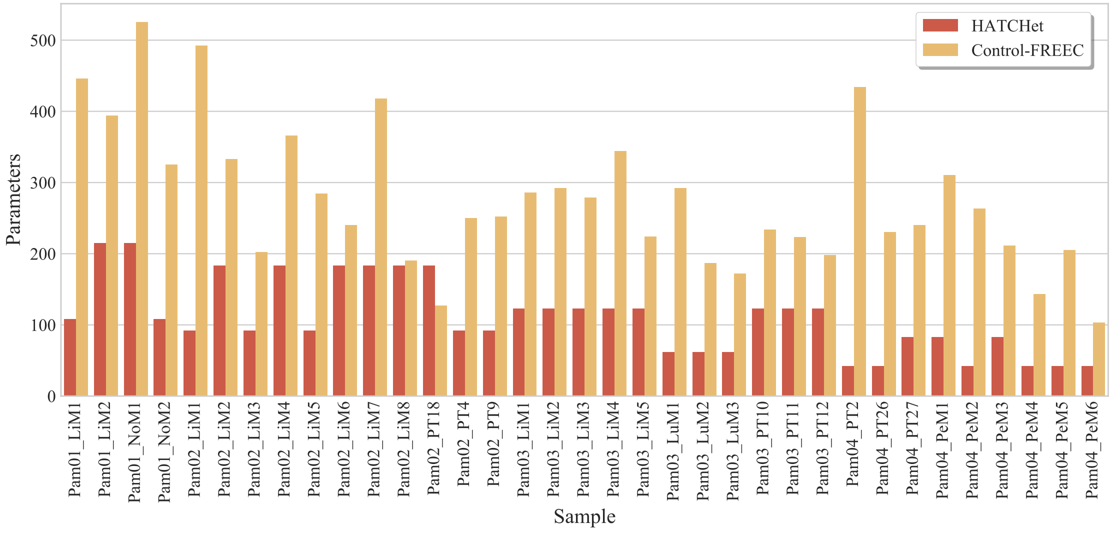

a

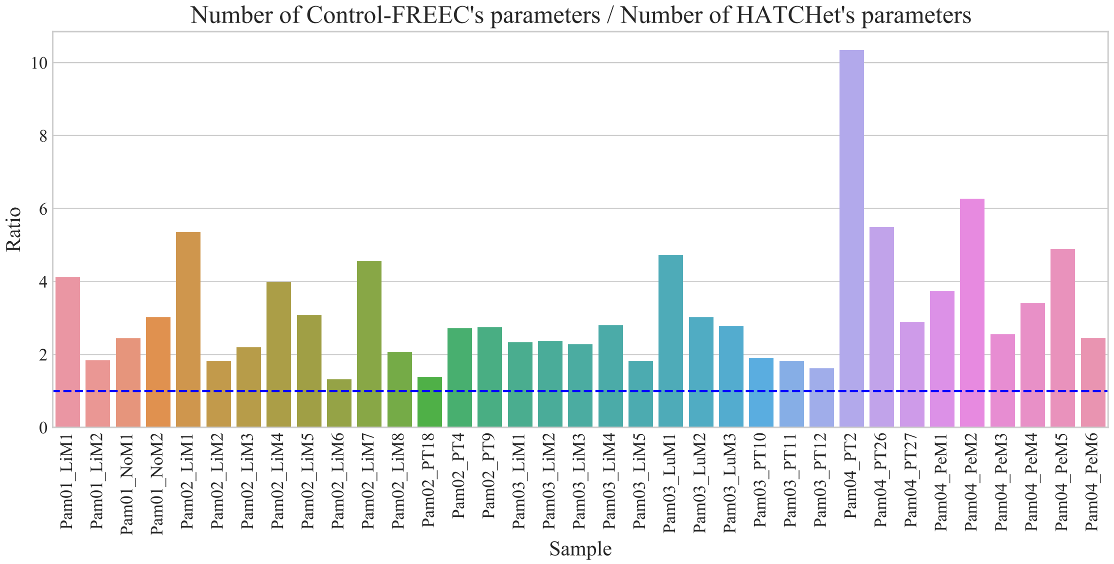

b

Supplementary Fig. 36: HATCHet uses three times less parameters than Control-FREEC for the results on the pancreas cancer dataset. a, The number of parameters used by HATCHet and Control-FREEC is computed for the solution provided for every sample of the pancreas cancer dataset. The number of parameters used by HATCHet for every sample corresponds to the number of allele-specific copy numbers for all clusters and the number of clone proportions. The number of parameters used by Control-FREEC for every sample corresponds to the number of total copy numbers for every segment. **b**, The ratio between the number of parameters used by Control-FREEC and the number of parameter used by HATCHet is computed for all samples. Control-FREEC constantly uses more parameters than HATCHet across all samples (blue dashed line indicates where the ratio is equal to 1) but one. More specifically, Control-FREEC uses on average at least 3 times more parameters than HATCHet.

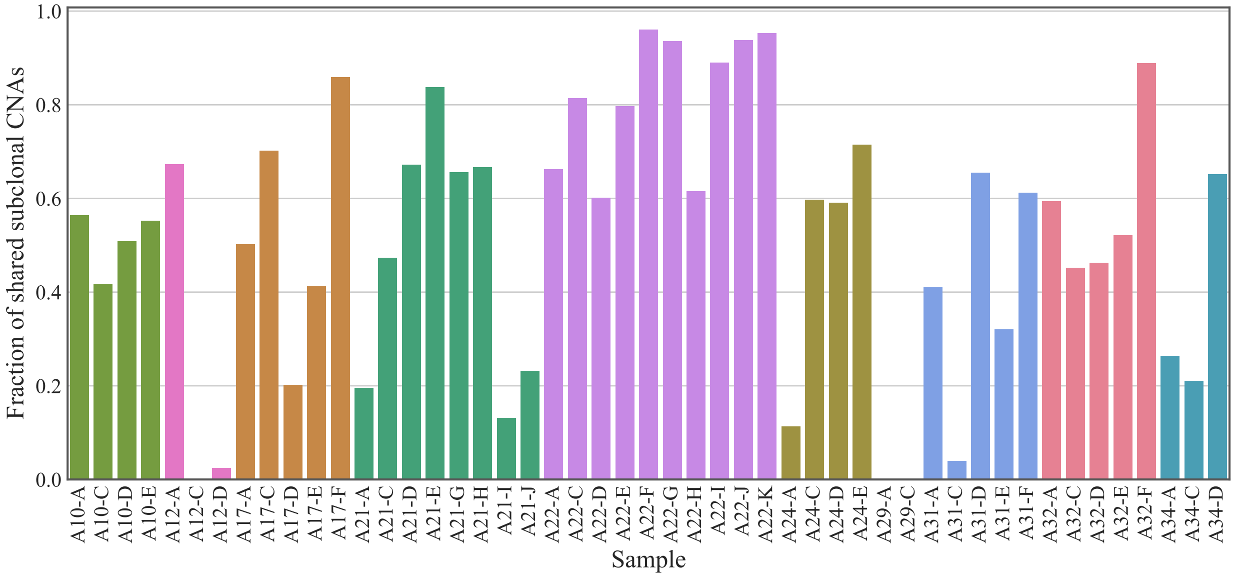

Supplementary Fig. 37: Battenberg infers shared subclonal CNAs in every sample of the prostate dataset and suggests polyclonal migrations in 9/10 patients. The fraction of the genome with shared subclonal CNAs previously inferred[3] by Battenberg is reported for all samples in every prostate cancer patient (indicated with different colors). A shared subclonal CNAs is defined as the same CNA (for the same genomic regions) which is shared by at least two samples from the same patient and it is subclonal in both samples. More specifically, a subclonal CNA in sample p corresponds to a sample-subclonal cluster s of genomic regions which have different copy-number states in distinct tumor clones present in p. We say that p shares the subclonal CNAs of s with another sample q if s is also a sample-subclonal cluster in q and there are at least two distinct copy-number states of s shared between p and q. Battenberg identifies extensive fractions of the genome with shared subclonal CNAs in all samples of all patients but the two samples of patient A29 and sample A12-C of patient A12. The only 3 samples that do not exhibit those shared subclonal CNAs are those in which Battenberg predicts presence or absence of a WGD differently from the other samples from the same patient. Since shared subclonal CNAs provide evidence for polyclonal migrations[3], these results suggest polyclonal migrations between 46/49 samples in 9/10 prostate cancer patients.

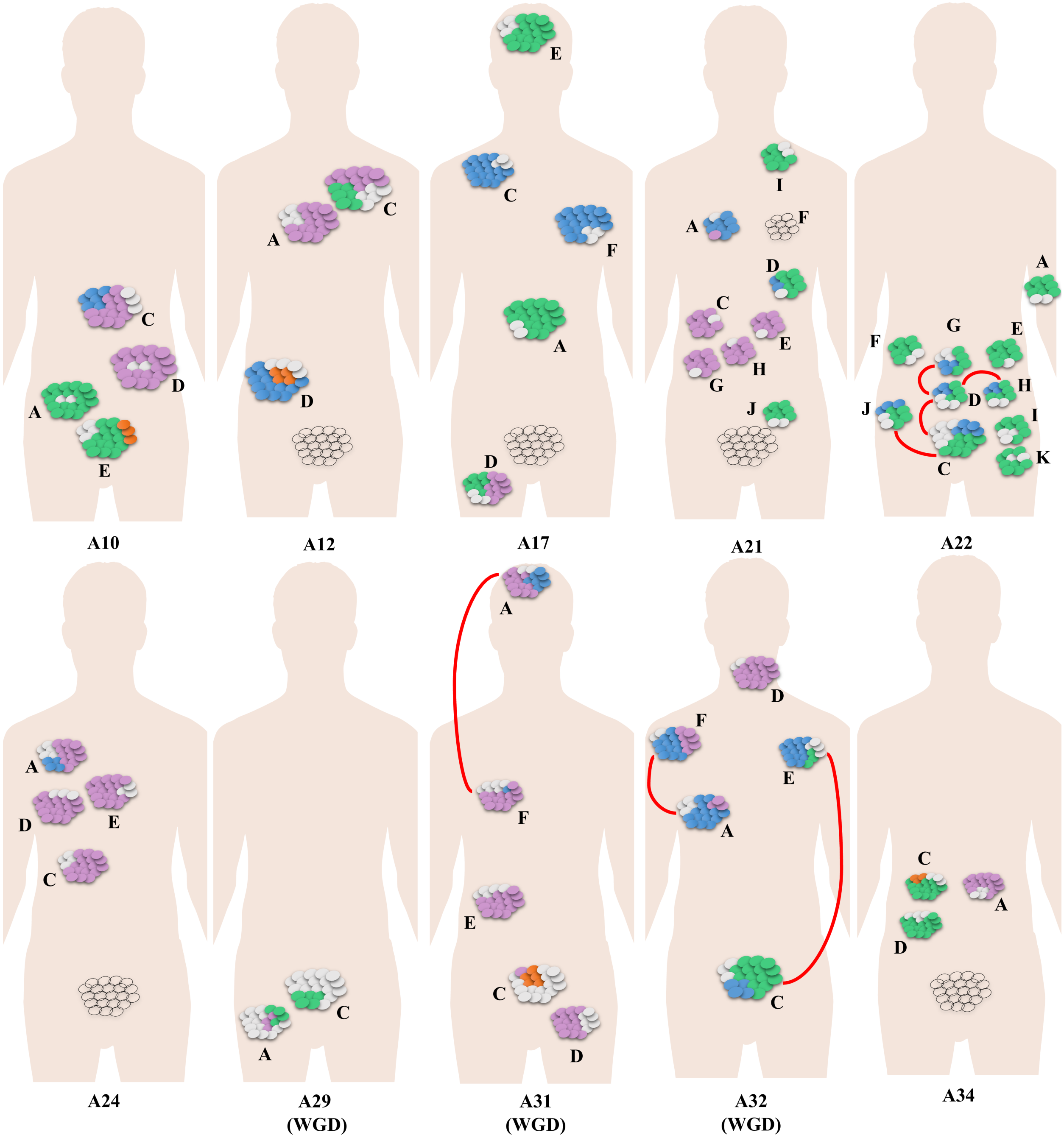

Supplementary Fig. 38: HATCHet infers tumor clones consistent with previous reports of polyclonal origin of metastasis in 3 prostate cancer patients. HATCHet infers a normal clone (gray ellipses) and one or more tumor clones (ellipses with an identifying color for each clone) shared across the samples of every prostate cancer patient (proportions of ellipses approximate the inferred clone proportions). HATCHet predicts a WGD for all tumor clones present in patients that are correspondingly labeled. Polyclonal origin of metastasis is supported in 3 patients (A22, A31, and A32) by the presence of samples that share multiple tumor clones (red edges). These 3 patients are part of the 5 patients previously reported with polyclonal migrations in the prostate publication[3] and the same 3 patients were also the only ones reported with polyclonal origin of metastasis in another analysis[4] of this dataset using the MACHINA algorithm for computing parsimonious migration histories identified.

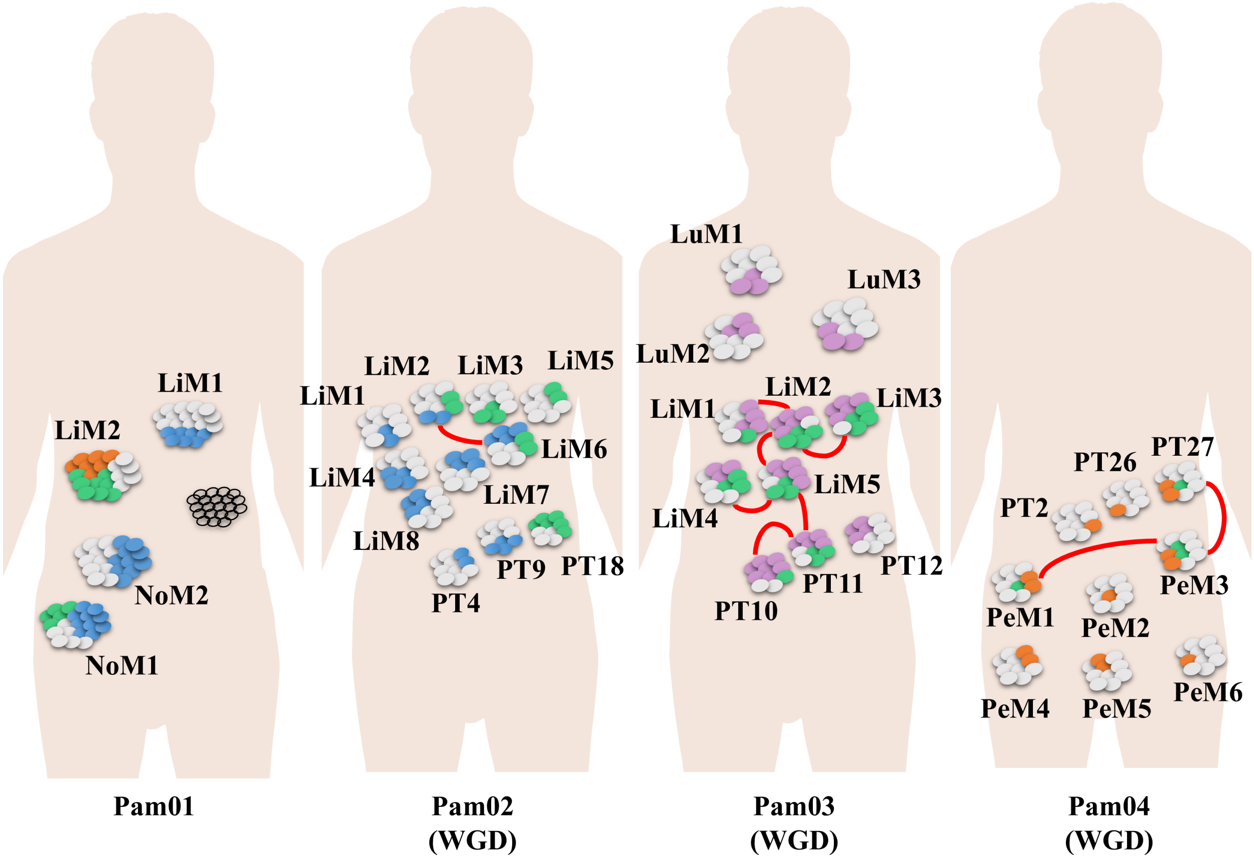

Supplementary Fig. 39: HATCHet infers tumor clones consistent with previous reports of limited heterogeneity and with unreported presence of polyclonal migrations in the pancreas cancer patients. HATCHet infers a normal clone (gray ellipses) and one or more tumor clones (ellipses with an identifying color for each clone) shared across the samples of every pancreas cancer patient (proportions of ellipses approximate the inferred clone proportions). HATCHet predicts a WGD for all tumor clones present in patients that are correspondingly labeled. The limited heterogeneity previously reported in the pancreas cancer study[5] is explained by the fact that most of the samples in all patients share at least one tumor clone. Also, the presence of metastatic samples that share multiple tumor clones (red edges) suggests the polyclonal origin of metastases in 3 patients (Pam02, Pam03, and Pam04), consistent with the reports of polyclonal migrations in mouse models of pancreas tumors[6].

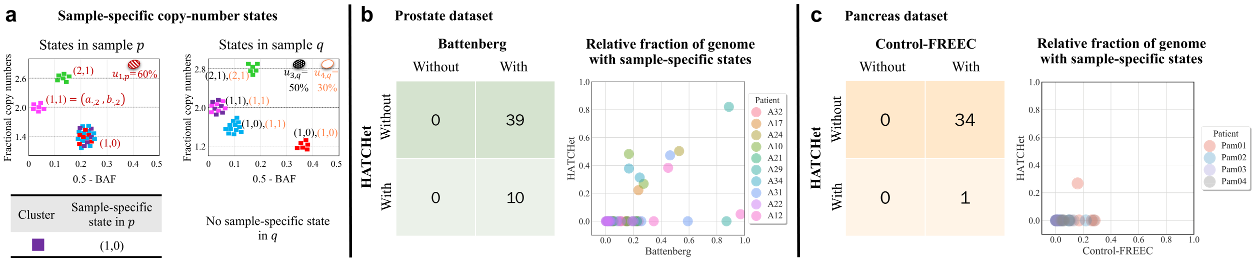

Supplementary Fig. 40: HATCHet identifies sample-specific states in a minority of tumor samples. **a**, Two samples p and q have 5 clusters with the correspondingly inferred copy-number states. While q does not have sample-specific states in any cluster, p has a sample-specific state associated with the purple cluster as this cluster is associated with the non-diploid state $(1, 0)$ only in p. **b**, On the prostate dataset, HATCHet identifies sample-specific states only in 10 samples, while Battenberg retrieves those in all the samples. HATCHet and Battenberg retrieves similar fractions of the genome with sample-specific states in the samples where both methods identify sample-specific copy-number states. Battenberg also retrieves a large fraction of the genome with sample-specific copy-number states in all the other samples. **c**, On the pancreas dataset, HATCHet identifies a single sample with sample-specific states, while Control-FREEC identifies sample-specific states in all samples. Control-FREEC infers large genomic regions with sample-specific copy-number states in nearly all samples.

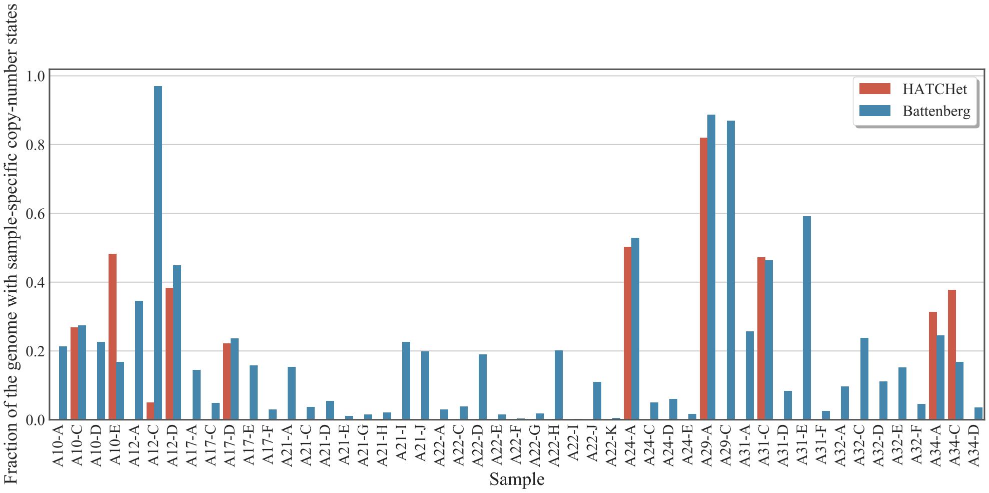

a

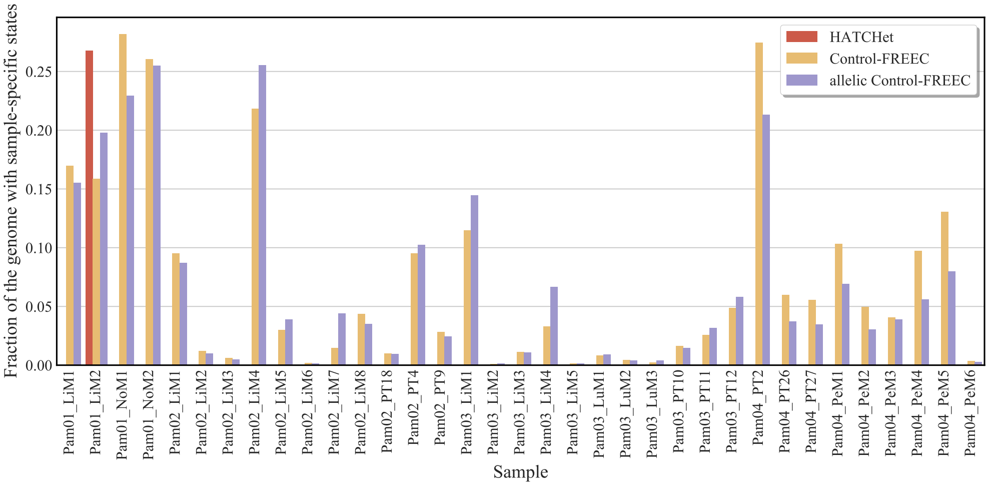

b

Supplementary Fig. 41: HATCHet identifies only few samples with sample-specific copy-number states in contrast to previous analyses. The fraction of the genome with sample-specific copy-number states is defined as the fraction of genomic positions with samples-specific copy-number states relative to the fraction of the genome with CNAs. **a**, While Battenberg identifies samples-specific copy-number states in all samples of the prostate cancer dataset for a substantial fraction of the genome, HATCHet identifies those only in 10 samples with similar fractions to those of Battenberg. **b**, While Control-FREEC identifies samples-specific copy-number states in all samples of the pancreas cancer dataset for a substantial fraction of the genome, HATCHet identifies those only in a single sample of patient Pam01. As Control-FREEC only infers total copy numbers, we computed the fraction of the genome with sample-specific copy-number states both by considering total copy number instead of copy-number states (Control-FREEC) and by splitting the total copy numbers into the allele-specific copy numbers that better fit the observed BAFs (allelic Control-FREEC).

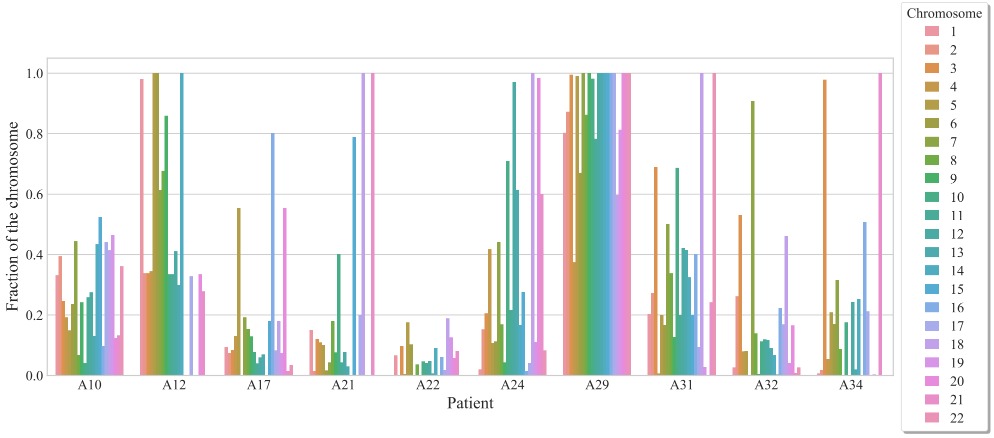

a

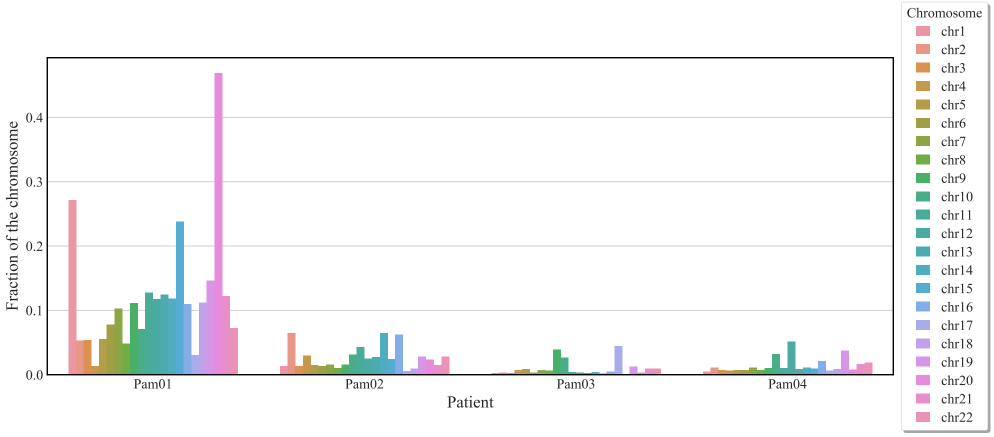

b

Supplementary Fig. 42: Battenberg and Control-FREEC infer many and large sample-specific CNAs in all chromosomes of every cancer patient. For every prostate and pancreas cancer patient, the fraction of genomic positions with sample-specific copy-number states in at least one sample is computed across all the chromosomes considering the copy numbers inferred by Battenberg (**a**) or Control-FREEC (**b**) relatively to the fraction of the genome with CNAs. The reported genomic regions with sample-specific copy-number states correspond to many and large sample-specific CNAs that are distributed in all chromosomes.

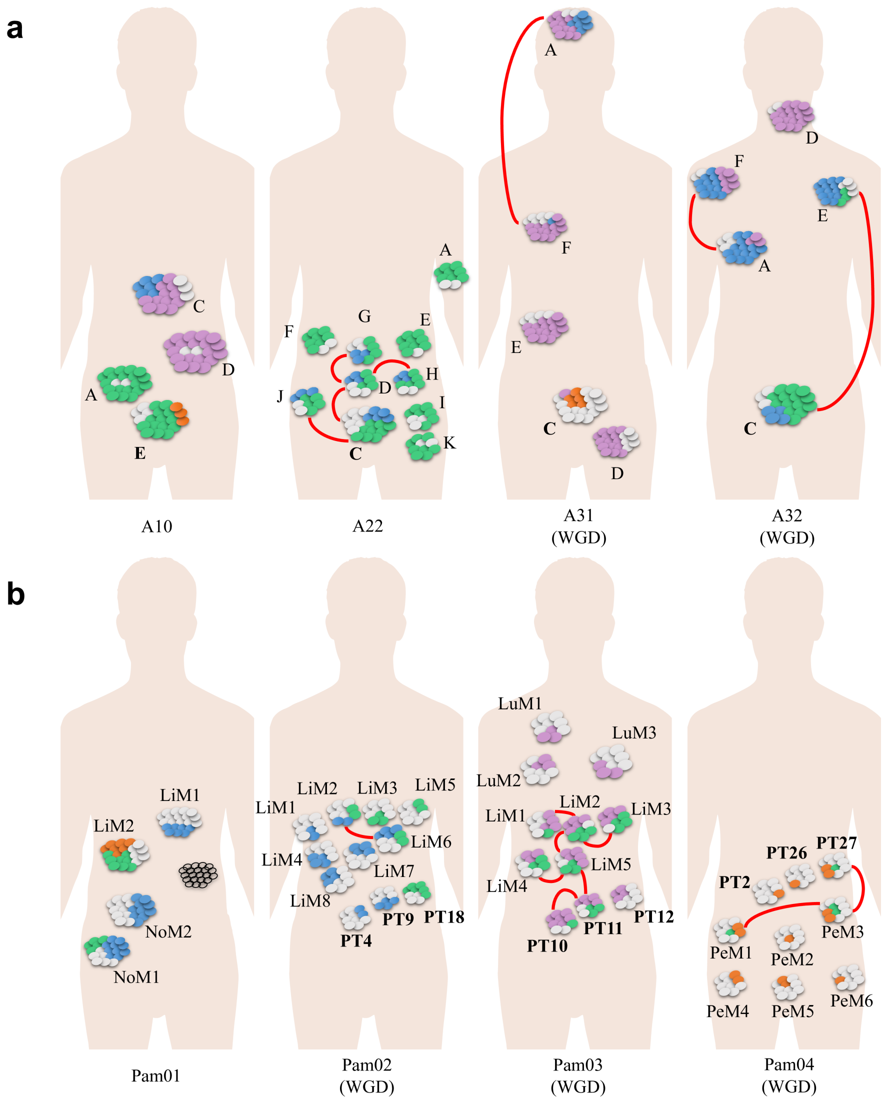

Supplementary Fig. 43: HATCHet identifies multiple tumor subclones shared across samples from the same patient, suggesting polyclonal origin of metastasis in some prostate and pancreas cancer patients. HATCHet infers a normal clone (gray ellipses) and one or more tumor clones (ellipses with an identifying color for each clone) shared across the samples of every patient (proportions of ellipses approximate the inferred clone proportions). Bold sample(s) are from primary tumor; other samples are metastases. Red arcs connect samples with two or more shared tumor clones, evidence of potential polyclonal migrations between anatomical sites. Patients for which HATCHet predicts a WGD are labeled correspondingly. **a**, The 3 prostate cancer patients (A22, A31, and A32) with multiple tumor clones shared between some samples (red arcs) are the same three patients that were inferred to have polyclonal seeding via the MACHINA algorithm[4], and a subset of the 5 patients reported to have polyclonal seeding in the original published analysis[3]. **b**, In pancreas cancer patient Pam01, lymph node metastasis sample NoM1 shares one tumor clone (blue) with a liver metastasis sample LiM1 and a different tumor clone (green) with a distinct liver metastasis sample LiM2, suggesting a role for lymph nodes in metastasis in this patient. The other 3 pancreas cancer patients (Pam02, Pam03, and Pam04) have multiple tumor clones shared between some samples (red arcs), evidence of potential polyclonal migrations between anatomical sites. Sharing of tumor subclones between anatomical sites was not considered in the original published analysis[5].

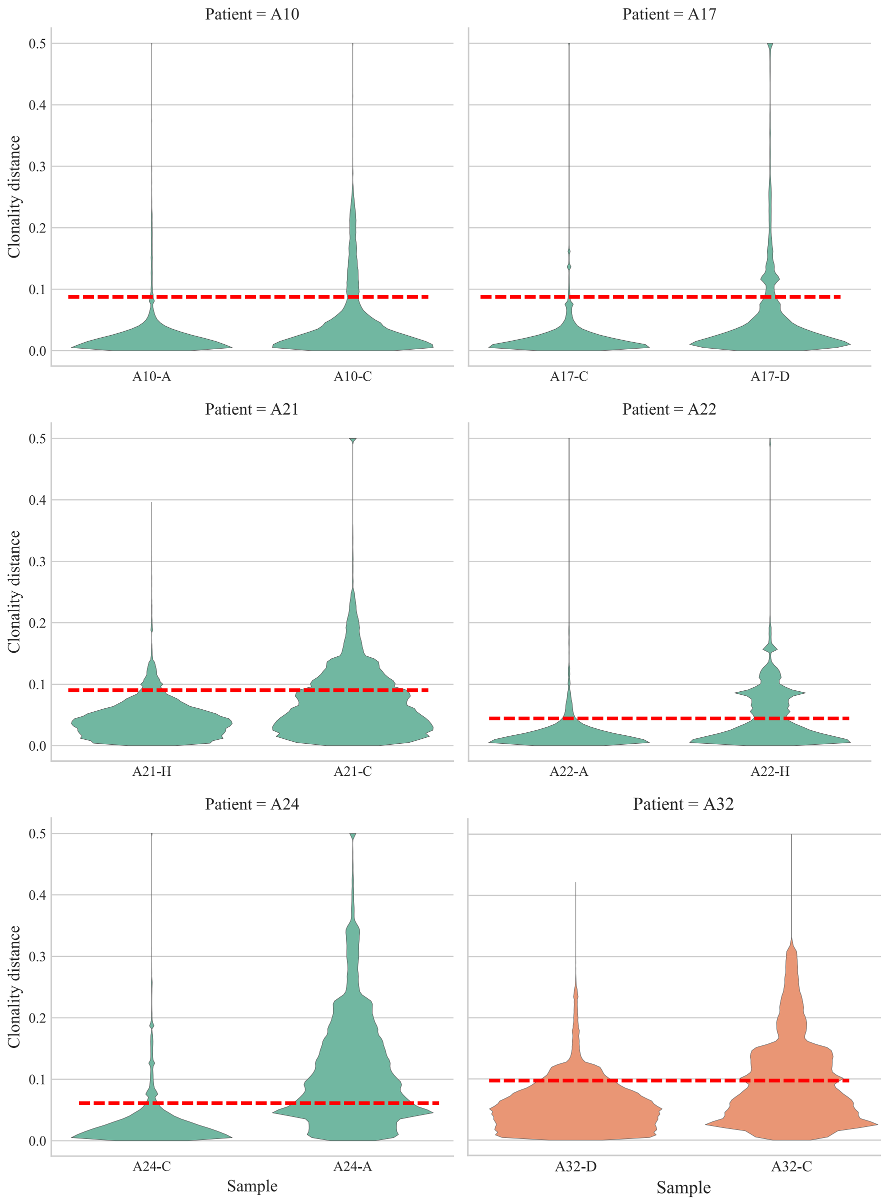

Supplementary Fig. 44: The clonality distance supports HATCHet's inference of subclonal CNAs in the prostate cancer dataset. The clonality distance is computed for every bin in two samples of six prostate cancer patients without (green) or with (orange) a WGD predicted by HATCHet. Violin plots show a kernel density estimate of the distributions of all values of clonality distance. The clonality distance is used as a quantitative measure to analyze the presence of subclonal CNAs directly from the signals of RDR and BAF; in fact, clusters of genomic regions with substantially higher values of the clonality distance indicate genomic regions with subclonal CNAs. For every patient, HATCHet infers the absence of subclonal CNAs for the sample on the left side and the presence of subclonal CNAs for the one on the right side. All the right-side samples inferred with subclonal CNAs correspondingly exhibit a large number of bins with substantially higher values of clonality distance, while all the left-side samples inferred without subclonal CNAs mostly have lower values (dashed red lines highlight thresholds for this difference).

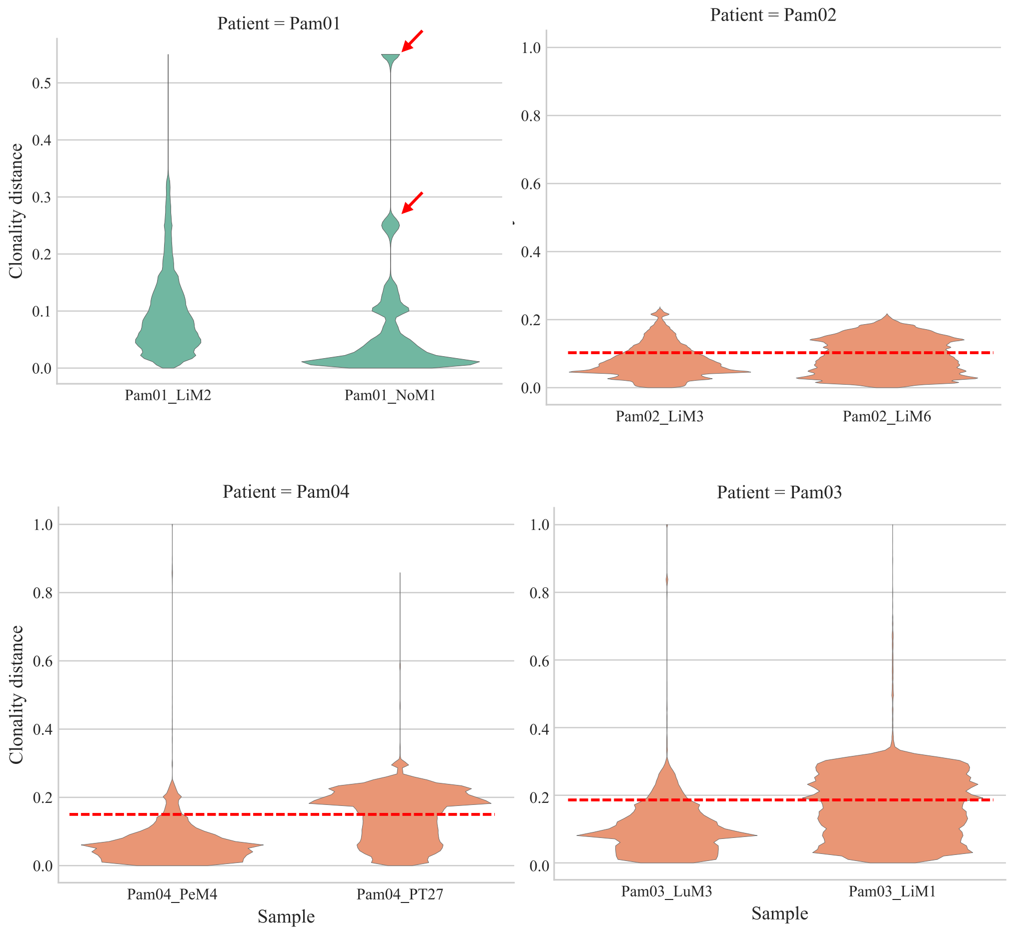

Supplementary Fig. 45: The clonality distance supports HATCHet's inference of subclonal CNAs in the pancreas cancer dataset. The clonality distance is computed for every bin in two samples of all four pancreas cancer patients without (green) or with (orange) a WGD predicted by HATCHet. Violin plots show a kernel density estimate of the distributions of all values of clonality distance. The clonality distance is used as a quantitative measure to analyze the presence of subclonal CNAs directly from the signals of RDR and BAF; in fact, clusters of genomic regions with substantially higher values of the clonality distance indicate genomic regions with subclonal CNAs. For every patient, HATCHet infers the absence of subclonal CNAs for the sample on the left side and the presence of subclonal CNAs for the one on the right side. All the right-side samples inferred with subclonal CNAs correspondingly exhibit a large number of bins with substantially high values of clonality distance, while all the left-side samples inferred without subclonal CNAs mostly have lower values (dashed red lines or arrows highlight thresholds for this difference).

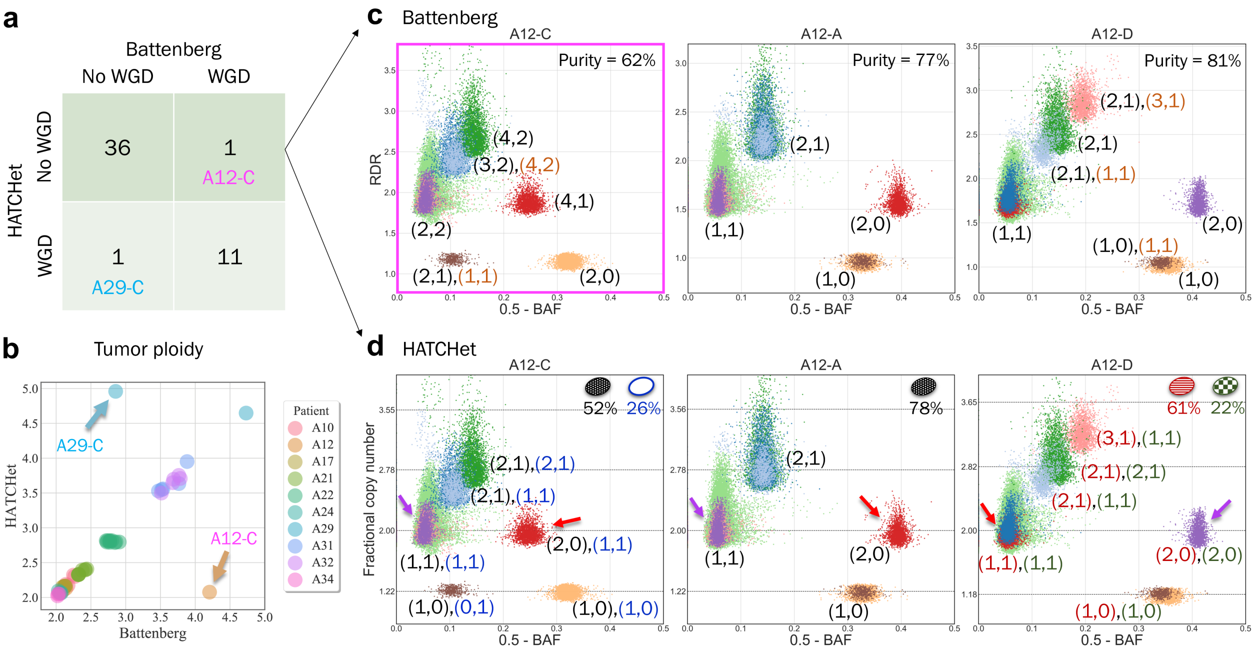

Supplementary Fig. 46: HATCHet predicts WGDs consistently across all samples from the same prostate cancer patient. **a**, HATCHet predicts absence/presence of WGDs in 47 of 49 samples in agreement with the published analyses based on manual review of Battenberg's tumor ploidy. While HATCHet predicts WGD consistently across all samples from the same patient, Battenberg predicts the presence of a WGD only in the sample A12-C of patient A12 and the absence of a WGD only in the sample A29-C of patient A29. **b**, The values of tumor ploidy predicted by HATCHet and Battenberg are nearly identical in all samples except A12-C and A29-C. **c**, The copy-number states inferred by Battenberg for three samples from patient A12, where a WGD was predicted only in sample A12-C. The larger number of clusters in the scaled BAF-RDR plot (each point corresponds to 50kb genomic bin) of A12-C could be explained by either the presence of subclonal CNAs or a WGD; however Battenberg infers both, even though it does not predict a WGD in the other two samples, A12-A and A12-D. The Battenberg's solution is also unlikely because of the copy-number states inferred for the purple cluster. The WGD in A12-C cannot occur after the complete loss of one allele for the purple cluster in sample A12-D, as the lost allele cannot be re-acquired. Moreover, the WGD in A12-C is unlikely to have occurred first, as many of the clusters in A12-D would then have to revert to their pre-WGD state. Thus, the only plausible explanation is that the WGD and transition of the purple cluster from the $(1,1)$ to the $(2,0)$ state occurred on different phylogenetic branches; however, even this explanation is unlikely, as other clusters in A12-D would also have to transition in a coordinated way on these parallel branches. Finally, the red and light-green clusters almost have the same RDR in A12-C but Battenberg infers different total copy numbers for these (4 vs. 5). **d**, HATCHet does not predict a WGD in any sample from patient A12, instead inferring the mixture of two subclones in samples A12-C and A12-D. Importantly, the red cluster is the only cluster in sample A12-C whose clonal/subclonal status differs from the Battenberg solution in (**c**). The position of the red cluster in the scaled BAF-RDR plot in A12-C is clearly intermediate between the positions of this cluster in other two samples (all with similar values of tumor purity), supporting HATCHet's interpretation of the red cluster in sample A12-C as a mixture of the copy-number states $(2,0)$ and $(1,1)$ of the red cluster in samples A12-A and A12-D, respectively.

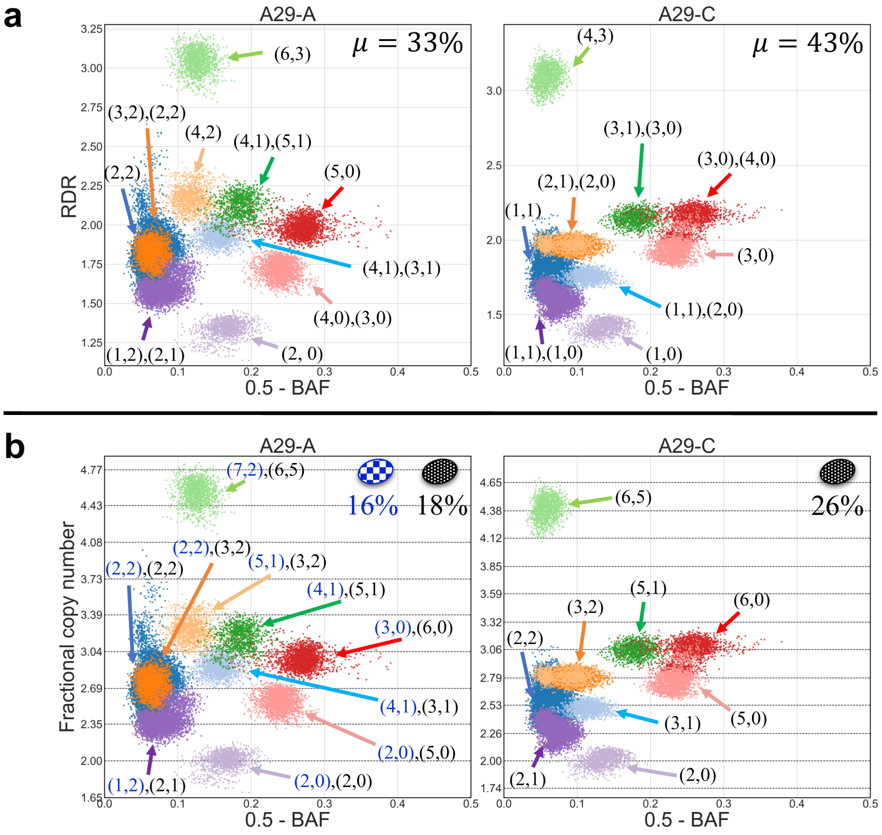

Supplementary Fig. 47: HATCHet infers simpler solutions and predicts a WGD consistently across all samples of prostate cancer patient A29. **a**, The solutions of Battenberg for two samples from patient A29 report the presence of a WGD only in sample A29-A and the absence of a WGD in sample A29-C with tumor purity (μ) equal to 33% and 43%, respectively. However, this difference between the two samples from A29 is unlikely for two main reasons. First, all clusters mostly preserve their relative positions in both plots. We expect this result is happening either when a WGD is present in only one sample and the allele-specific copy numbers of all clusters are doubled in this sample compared to the other or when the presence/absence of a WGD is the same in both samples. While Battenberg infers allele-specific copy number that are clearly doubled only for the diploid/tetraploid cluster (dark blue cluster), all the other clusters have allele-specific copy numbers in sample A29-A that are very similar to their allele-specific copy numbers in A29-C. Second, nearly all the clusters with subclonal CNAs in A29-C are equally associated with subclonal CNAs in A29-A. As such, the inference of a WGD in A29-A does not reflect the trade-off between subclonal CNAs and WGD that we would have expected. **b**, The solutions of HATCHet report the presence of a WGD in both samples from patient A29. HATCHet infers the presence of a single tumor clone (black clone) with tumor purity of 26% in sample A29-C as the fractional copy numbers of all clusters are compatible with sample-clonal clusters (in correspondence of horizontal grid lines). HATCHet infers the presence of the same clone plus an additional tumor clone (blue clone) in A29-A with a tumor purity of 34% (clone proportions are reported for both tumor clones); as such, HATCHet infers subclonal CNAs for all clusters (violet, pink, cyan, red, dark green, yellow, orange, and light green) that do not have a fractional copy number compatible with sample-clonal clusters (in correspondence of horizontal grid lines). This is a simpler solution than the one in (**a**) and is likely for two main reasons. First, all clusters mostly preserve their relative positions in the two plots and, in fact, HATCHet infers similar allele-specific copy numbers for every cluster in the two samples. Second, the clusters that slightly vary their relative positions between the two plots are indeed associated with subclonal CNAs.

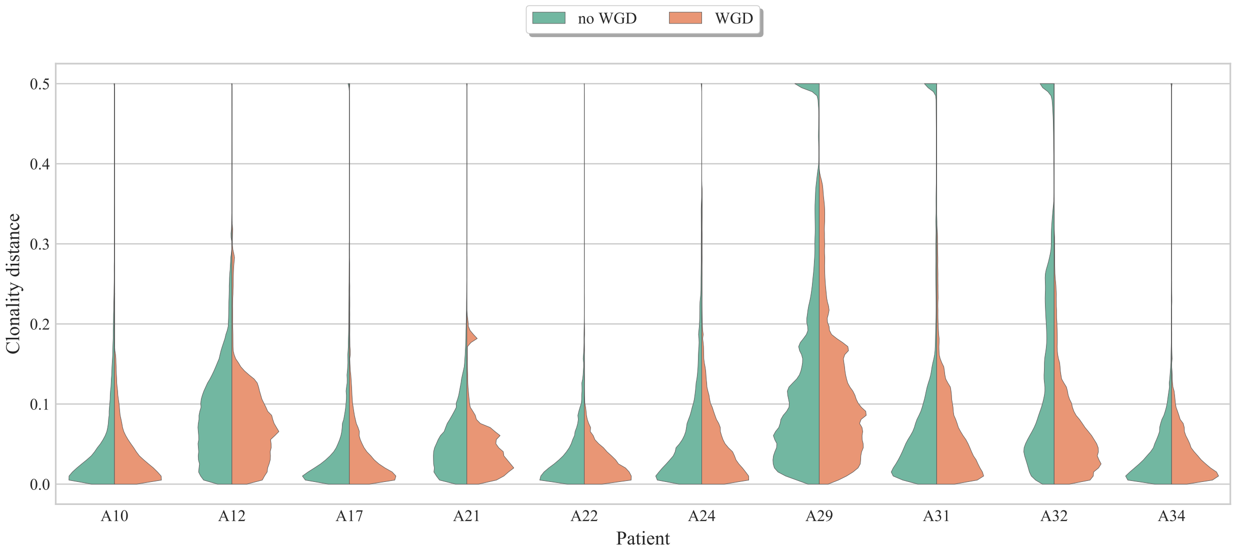

a

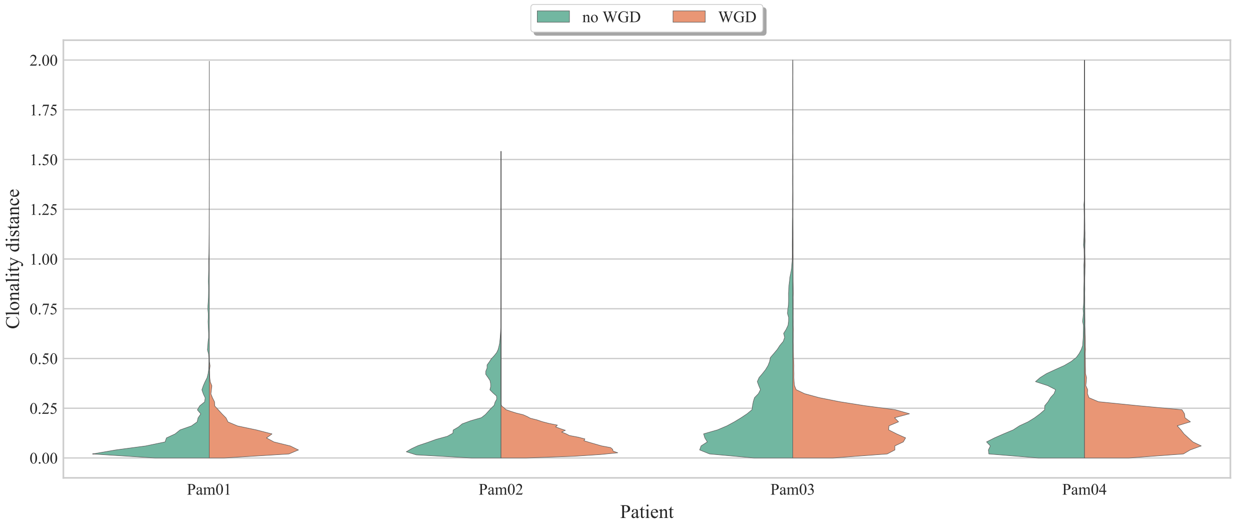

b

Supplementary Fig. 48: The clonality distance supports HATCHet's predictions of WGDs in the prostate and pancreas cancer datasets. The clonality distance is computed for every 50kb genomic bin in all the samples of every prostate and pancreas patient when either assuming the absence (green) or the presence (orange) of a WGD. As high values of the distance indicate genomic regions with subclonal CNAs, the clonality distance is used to investigate the trade-off between the inference of subclonal CNAs or WGDs. The values of the clonality distance are also normalized by the corresponding values of tumor purity to allow the comparison across multiple samples. Each side of the violin plots shows a kernel density estimate of the distributions of all values of clonality distance. **a**, In all prostate cancer patients where HATCHet does not predict a WGD (A10, A12, A17, A21, A22, A24, and A34) there is no clear difference in the values of the clonality distance between the two assumptions. In contrast, in all prostate cancer patients where HATCHet predicts a WGD (A29, A31, and A32) there is a large number of bins with higher values of clonality distance only when assuming the absence of a WGD. **b**, In all pancreas cancer patients where HATCHet does not predict a WGD (Pam01) there is no clear difference in the values of the clonality distance between the two assumptions. In contrast, in all pancreas cancer patients where HATCHet predicts a WGD (Pam02, Pam03, and Pam04) there is a large number of bins with higher values of clonality distance only when assuming the absence of a WGD.

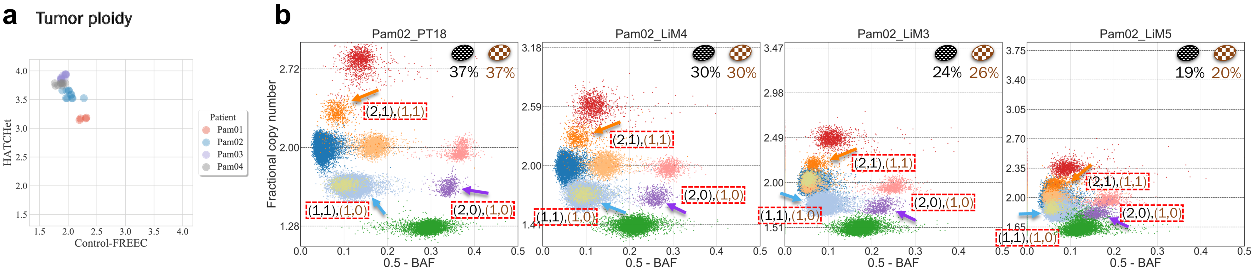

Supplementary Fig. 49: HATCHet identifies WGDs in three of four pancreas cancer patients. **a**, The tumor ploidy estimated by HATCHet is correspondingly high (> 3) for all these 31 samples with a WGD, while the tumor ploidy in published analyses is approximately 2 for all samples. While HATCHet also estimates high ploidy for the 4 samples of patient Pam01 (red points), HATCHet does not predict a WGD in this case but massive rearrangements. **b**, The best diploid (no WGD) solution produced by HATCHet on the same four samples contains three tumor clones (ellipses with related proportions), and each sample is a mixture of 2-3 clones. Curiously, three clusters (cyan, purple, and orange arrows) that were tumor-clonal in the WGD solution in Supplementary Fig. 5b are instead inferred to be sample-subclonal in the diploid solution, but with the same pair of copy number states and the same clone proportions (sum of black and brown clone proportions) in every sample. Thus, the extra inferred clone is a result of overfitting the data, due to too few copy number states for clonal clusters in a diploid solution.

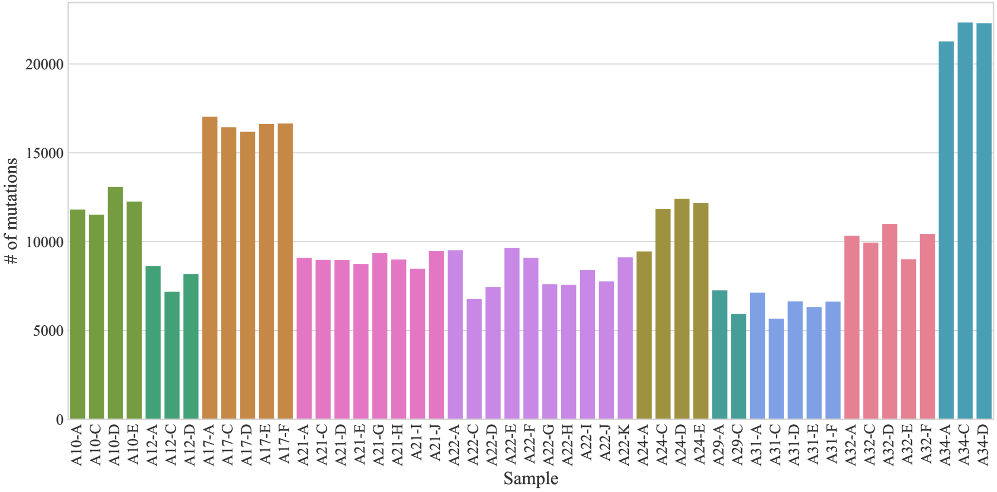

a

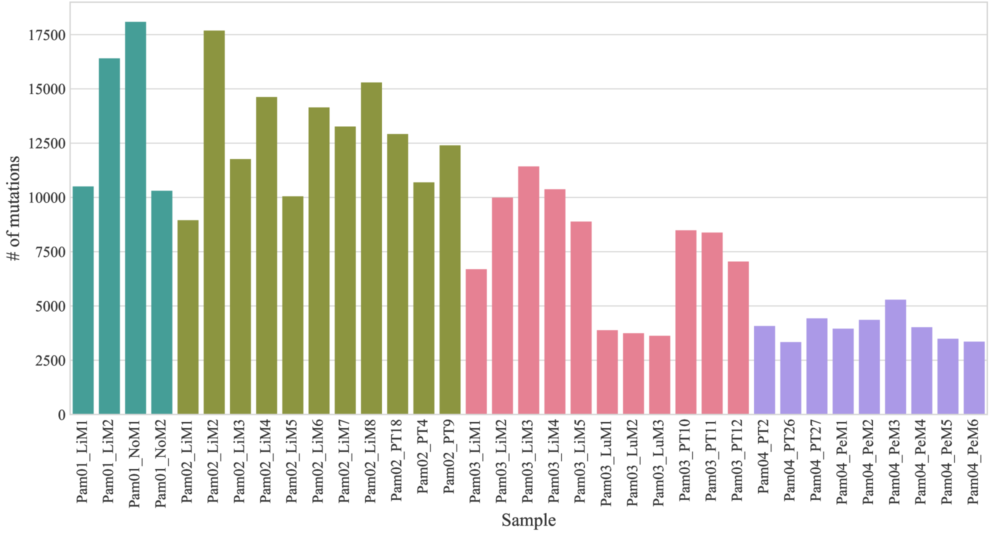

b

Supplementary Fig. 50: Number of somatic SNVs and small indels inferred from the samples of the prostate and pancreas cancer datasets. Each bar represents the count of somatic SNVs and small indels in a sample and is colored according to the corresponding prostate (**a**) and pancreas (**b**) cancer patient.

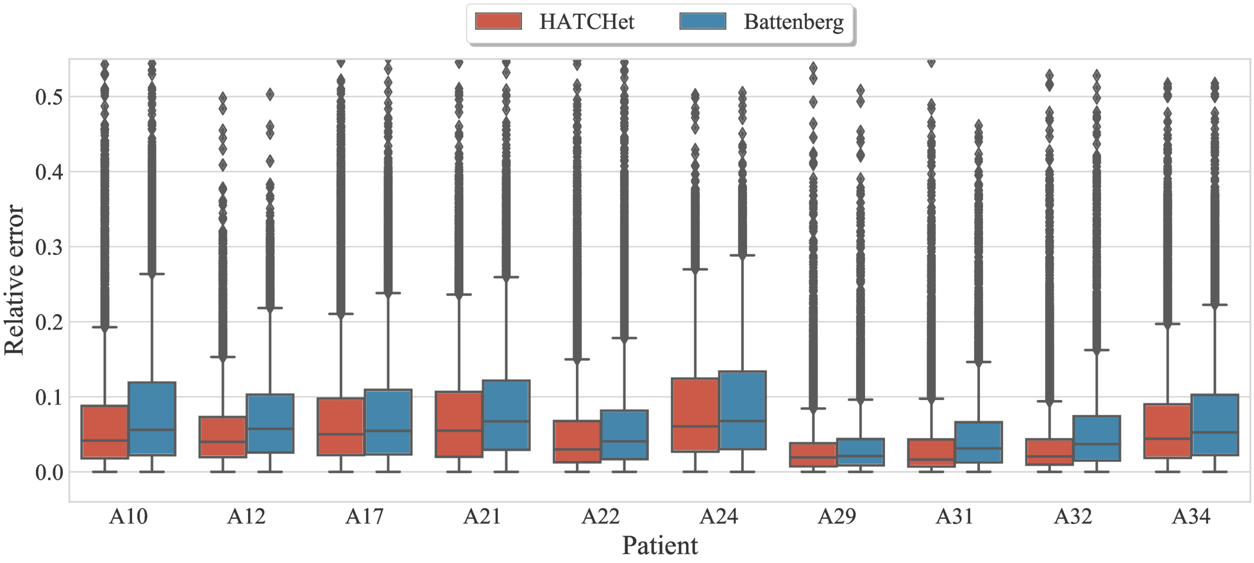

Supplementary Fig. 51: HATCHet consistently estimates predicted VAFs with lower errors than Battenberg on all prostate cancer patients. The relative error is computed between the observed and predicted VAF for every mutation in the samples of every prostate cancer patient by considering the copy numbers and proportions inferred by HATCHet and Battenberg. Box plots show the median and the interquartile range (IQR), and the whiskers denote the lowest and highest values within 1.5 times the IQR from the first and third quartiles, respectively.

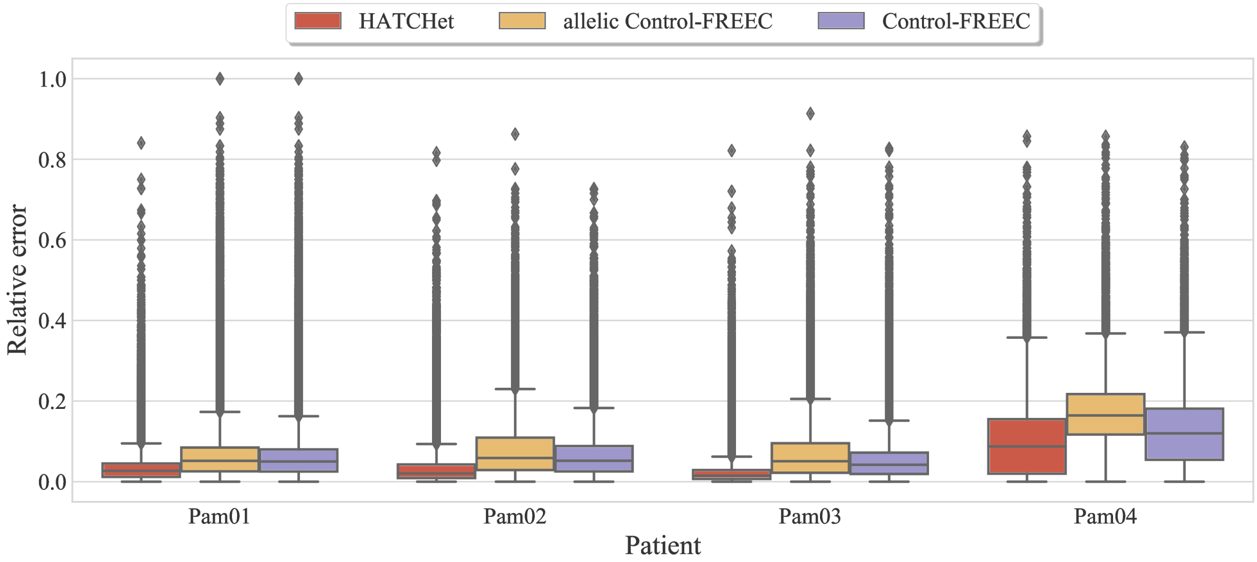

Supplementary Fig. 52: HATCHet consistently estimates predicted VAFs with lower errors than Control-FREEC on all pancreas cancer patients. The relative error is computed between the observed and predicted VAF for every mutation in the samples of every pancreas cancer patient by considering the copy numbers and proportions inferred by HATCHet and Control-FREEC. Since Control-FREEC only infers total copy numbers, we estimated the VAF both by considering total copy numbers without constraining the mutation to a single allele (Control-FREEC) and by considering the allele-specific copy numbers that better fit to the observed BAF (allelic Control-FREEC). Box plots show the median and the interquartile range (IQR), and the whiskers denote the lowest and highest values within 1.5 times the IQR from the first and third quartiles, respectively.

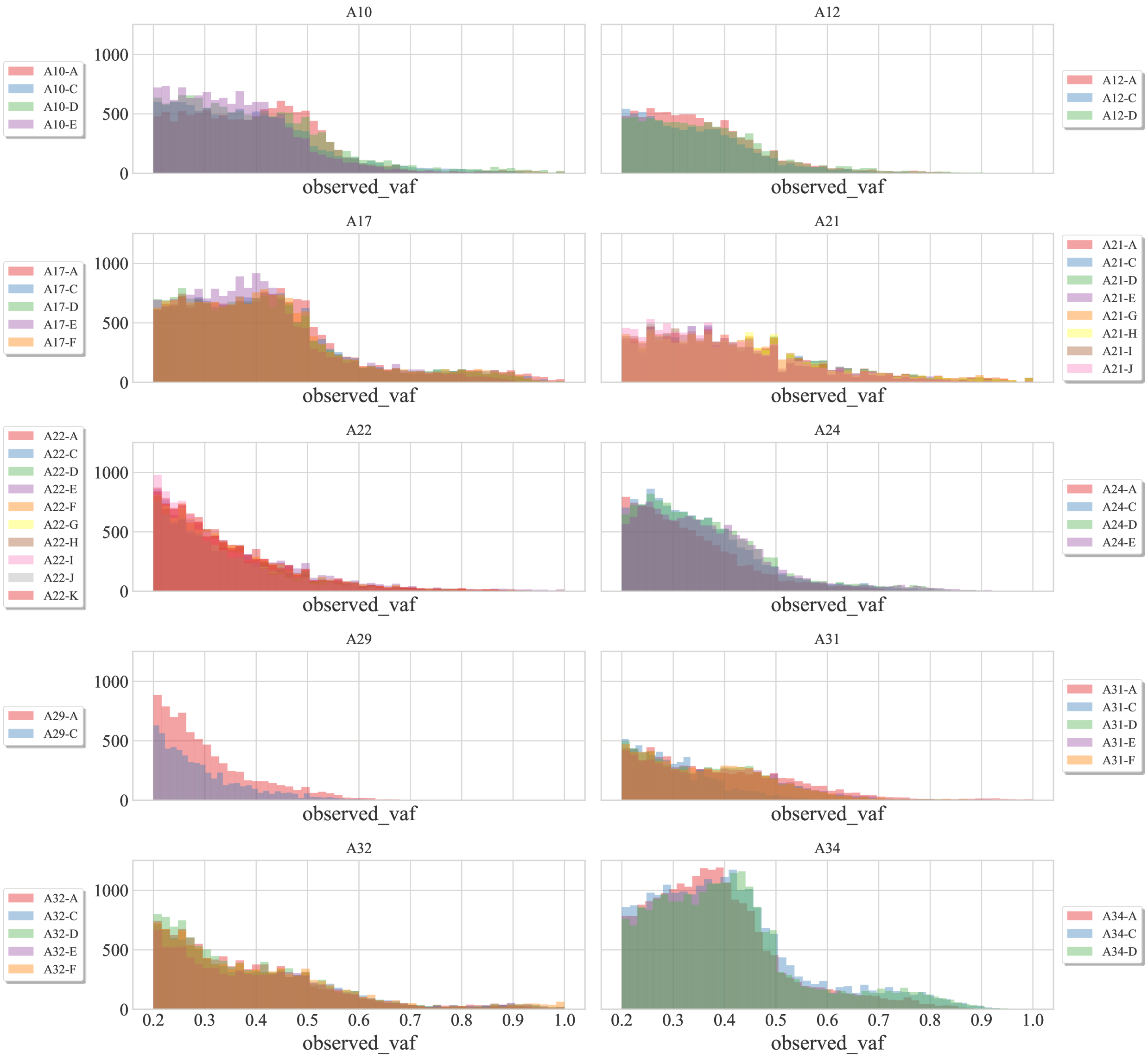

Supplementary Fig. 53: Observed VAF of somatic mutations for all samples of the prostate cancer patients. The observed VAF of each somatic mutation is computed as the ratio of the sequencing reads harboring the mutation over all the reads covering the corresponding genomic position. Each plot corresponds to a prostate cancer patient and represents the distribution of the VAFs for all somatic mutations (including somatic SNVs and small indels) inferred from each sample (color).

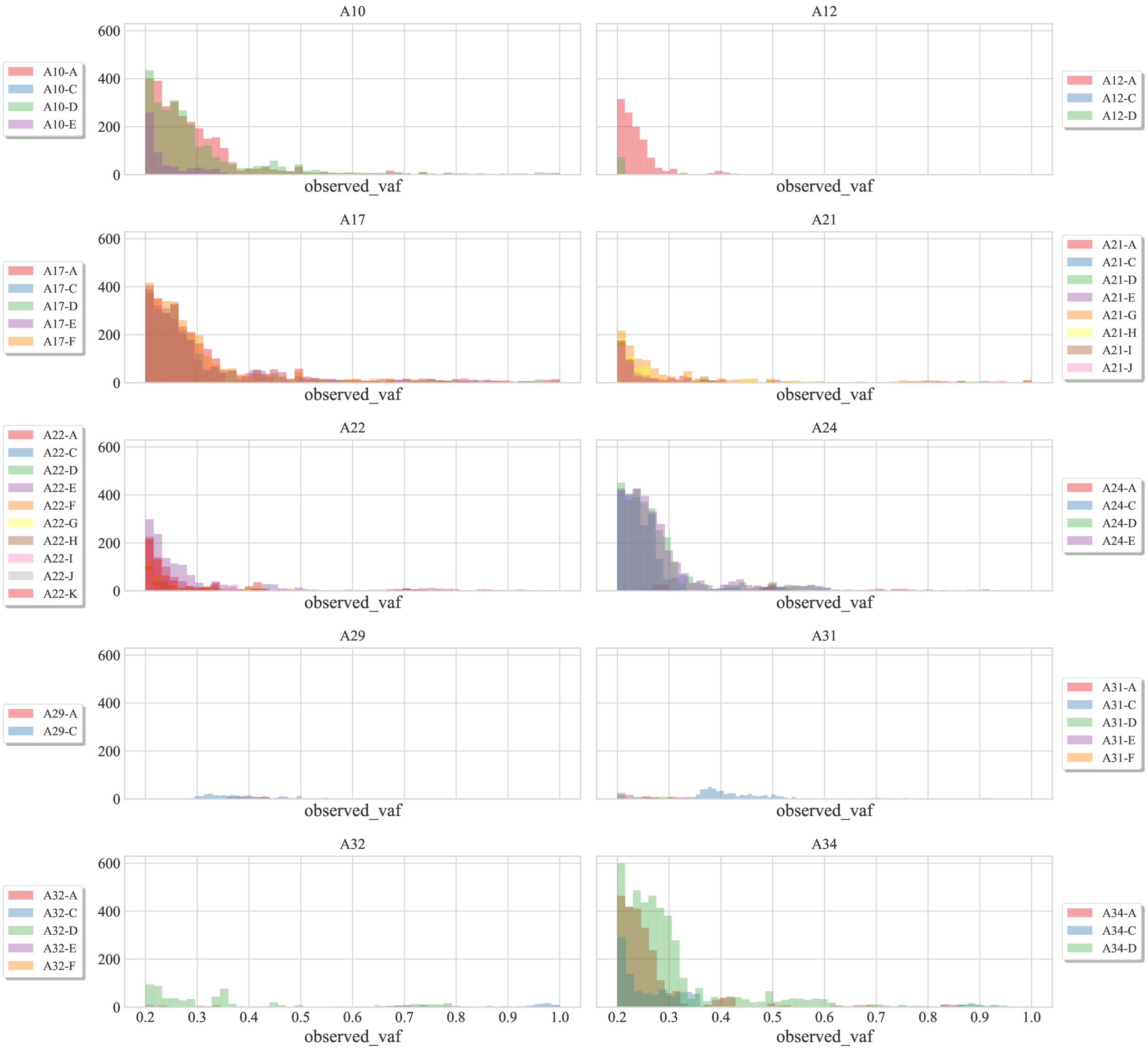

Supplementary Fig. 54: Somatic mutations non-explained by HATCHet have low VAF in the samples of the prostate cancer patients. The samples of most patients where HATCHet does not predict a WGD (A10, A17, A21, A22, A24, and A34) have several somatic mutations that are not explained by the copy numbers and clone proportions inferred by HATCHet. These mutations mostly have low values of observed VAF (≤ 0.3).

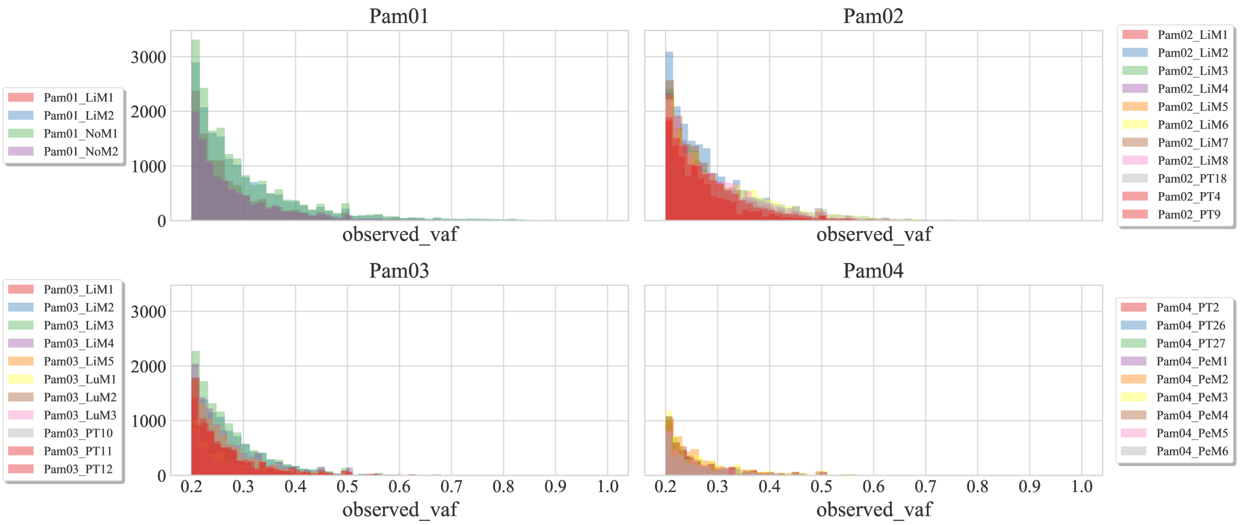

Supplementary Fig. 55: Observed VAF of somatic mutations for all samples of the pancreas cancer patients. The observed VAF of each somatic mutation is computed as the ratio of the sequencing reads harboring the mutation over all the reads covering the corresponding genomic position. Each plot corresponds to a pancreas cancer patient and represents the distribution of the VAFs for all somatic mutations (including somatic SNVs and small indels) inferred from each sample (color).

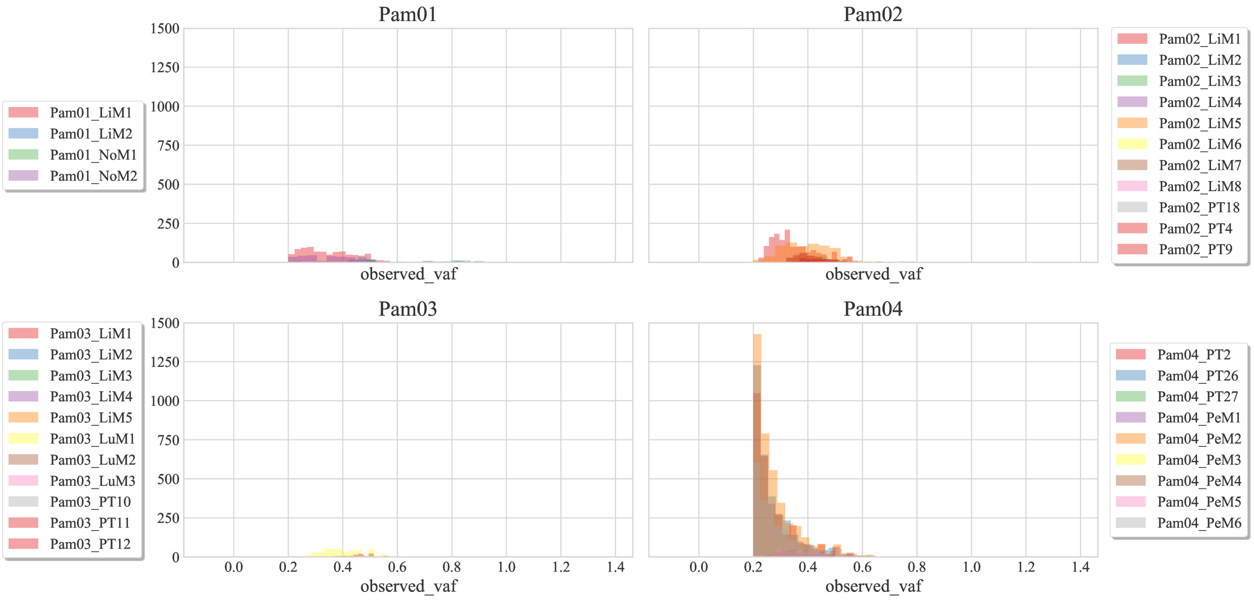

Supplementary Fig. 56: HATCHet explains most of the somatic mutations in the samples of 3/4 pancreas cancer patients. Most of the somatic mutations in the samples of 3 patients (Pam01, Pam02, and Pam03) are explained by the copy numbers and clone proportions inferred by HATCHet. In contrast, the samples of the remaining patient (Pam04) have several mutations not explained by HATCHet.

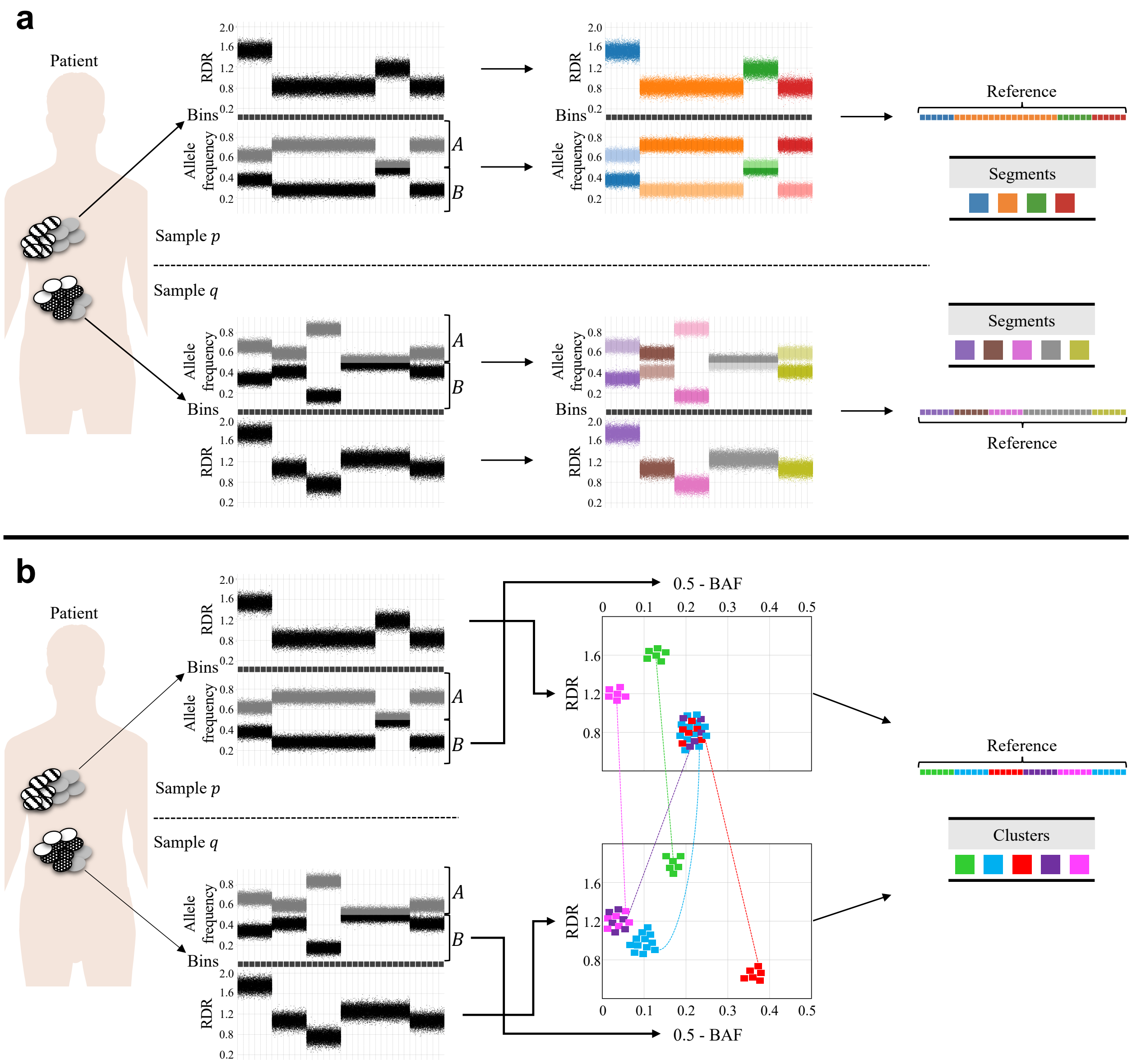

Supplementary Fig. 57: Local clustering and global clustering of genomic regions. Methods for CNA inference aim to identify the genomic segments that result from the accumulation of CNAs by splitting the reference genome into short genomic bins and clustering these bins in terms of RDR and BAF. Each segment thus comprises genomic regions that have the same copy-number states in all clones. **a**, Existing methods locally cluster neighboring bins, separately in each sample. As such, 4 and 6 segments (different colors) are identified in the two samples, respectively. **b**, HATCHet globally clusters the bins along the entire genome and jointly across multiple samples. As such, the reference genome is represented in a two-dimensional BAF-RDR plot for each sample, respectively, and HATCHet identifies 5 clusters (colors). Each cluster contains the same set of bins in all samples and may have different positions in each plot according to the different values of RDR and BAF of the included bins in the corresponding sample. Differently to the local clustering of existing methods, a cluster may contain non-neighboring bins (e.g. cyan bins) and bins in different clusters may have the same values of RDR and BAF in a sample (e.g. red, purple, and cyan in sample *p*) but not in all (e.g. red, purple, and cyan in sample *q*).

Supplementary Tables

Feature	HATCHet	Existing methods	Notes and exceptions
Integer copy numbers	Allele-specific copy numbers	Allele-specific copy numbers	Control-FREEC and THetA only infer total copy numbers
Copy-number model	Segments are **dependent** and modeled as **clones**	Segments are **independent**	THetA, cloneHD, CELLULOID, Canopy, and ReMixT model **clones**
	Samples are **dependent**	Samples are **independent**	• cloneHD: **every** sample contains the **same few** (2-3) clones<br>• Canopy: **only** jointly with SNVs, **manual** selection of major CNAs, and **known** fractional copy numbers
Clustering genomic regions	Globally along **genome** and **jointly** across **samples**	Locally for **neighboring** regions **per sample**	CELLULOID and FACETS: cluster **inferred segments** across genome **independently for each sample**
Fractional copy numbers	**Multiple values** estimated: with/without **WGD**	**No** direct **estimation**	• FALCON estimates **unique** value independently per sample<br>• Canopy **requires** fractional copy numbers in input
Model selection	Based on clonal composition: **trade-off** between **number of clones** and **WGD**	Based on values of tumor **purity** and **ploidy**	

Supplementary Table 1: Comparison of HATCHet and existing methods for copy-number deconvolution. Comparison of the main features of HATCHet and existing methods for copy-number deconvolution. The existing methods that are considered in this comparison are: ABSOLUTE[7], ASCAT[8], Battenberg[9], FALCON[10], Canopy[11], Sequenza[12], TITAN[13], FACETS[14], sclust[15], Control-FREEC[16], THetA[17,18], cloneHD[19], CELLULOID[20], and ReMixT[21].

Inferred parameter	HATCHet's step	Definition
m	Step (2) in first module	Number of clusters of genomic bins
$F^A = [f^A_{s,p}], F^B = [f^B_{s,p}]$	Step (3) in first module	Fractional copy number of segment/cluster s in sample p according to the occurrence or not of a WGD
γ_p	Step (3) in first module	Scale factor of every sample p according to the occurrence or not of a WGD
n	Step (5) in second module	Number of clones present across all k samples, including the normal diploid clone
Occurrence or not of a WGD	Step (5) in second module	
$A = [a_{s,i}], B = [b_{s,i}]$	Steps (4)-(5) in second module	Allele-specific copy numbers of segment/cluster s in clone i
$U = [u_{i,p}]$	Steps (4)-(5) in second module	Clone proportion of clone i in sample p

Supplementary Table 2: Parameters inferred by HATCHet. The table summarizes every parameter inferred by HATCHet, providing the HATCHet step where the inference is described and the corresponding definition.

Model selection parameters	Definition	Default value
c_{max}	Maximum total copy number	8 without WGD and 12 with WGD (HATCHet can also automatically infer c_{max} by rounding F^A and F^B)
u_{min}	Minimum clone proportion	0.03 (value can be increased when proportions U are equal to u_{min} and suggest overfitting)
$\mathfrak{U}$	Maximum number of clones	10 (value can be increased according to number of samples and when inferred n is close to the bound)
ε	Initialization of elbow criterion	0.35 (higher/lower values increase/decrease confidence of having only one tumor clone across all samples, i.e. $n = 2$)
ξ	Sensitivity to small CNAs	$+\infty$ (value 0.6 provides in practice a good trade-off between higher n and data noise, while higher/lower values decrease/increase the sensitivity)

Supplementary Table 3: Model selection parameters and their default values. The table summarizes all the model selection parameters, providing the corresponding definition and the corresponding default value.

Supplementary Methods

1 On the model of fractional copy numbers, tumor ploidy, and tumor purity

In this section, we explicitly model fractional copy numbers, tumor ploidy, and tumor purity according to the model defined in Methods. More specifically, we first formally define RDR and we show that RDR is directly proportional to the corresponding fractional copy number. Second, we formally define BAF and show that BAF estimates the proportion of B-specific fractional copy numbers. Last, we model the parameters of tumor ploidy and tumor purity to show that these are composite parameters depending on the unknown copy numbers and clone proportions. In addition, we show that tumor ploidy is equivalent to other related parameters that are considered by current methods, as the haploid coverage.

The models that we present in this section are based on the definitions previously described in Methods. Remember that we consider clusters of genomic bins instead of segments as described in Methods, using the same definitions for simplicity. In addition, we define the direct observations that we obtain from the DNA sequencing of k bulk tumor samples and a matched-normal sample. More specifically, we observe sequencing reads that are mapped to a human reference genome and, following the procedures later described in Supplementary Method 2, we have the following observations for the genomic regions of every cluster s:

1. A total read count $\tau_{s,p}$ for s in a bulk tumor sample p;
2. Allele-specific read counts $\tau^A_{s,p}, \tau^B_{s,p}$ for s in a bulk tumor sample p;
3. A total read count ν_s for s in the matched-normal sample.

1.1 Fractional copy numbers are directly proportional to RDRs

We show that the fractional copy number $f_{s,p}$ and the corresponding RDR $r_{s,p}$ of a cluster s in a sample p are directly proportional. The RDR $r_{s,p}$ is defined as the ratio between the read count $\tau_{s,p}$ in p and the corresponding read count ν_s in the matched-normal sample, i.e.

$$r_{s,p} = \frac{\tau_{s,p}}{\nu_s}. \quad (1)$$

Suppose that a total of R_p sequencing reads of length D have been sequenced uniformly from the genome of E_p cells present in p. Also, let L_i be the genome length of the cells that belong to clone i, such that $L_1 \approx 6 \cdot 10^9$ bases for the normal clone since the reference genome comprises $L \approx 3 \cdot 10^9$ bases and $L_1 = 2L$. Following a direct extension of the Lander-Waterman equation[22], the expected number of reads obtained from a single copy of a genomic position of an individual cell is

$$\frac{DR_p}{\sum_{1\leq i\leq n} E_p u_{i,p} L_i} \quad (2)$$

because there are $E_p u_{i,p}$ cells belonging to clone i whose genome length is L_i. In fact,

$$\frac{D}{\sum_{1\leq i\leq n} E_p u_{i,p} L_i} \quad (3)$$

is the probability that a read is sequenced from a single copy of a genomic position of an individual cell. The read count $\tau_{s,p}$ corresponds to the total number of reads obtained from all the copies of the genomic positions in s from all cells, and is hence equal to

$$\tau_{s,p} = \frac{DR_p}{\sum\limits_{1 \leq i \leq n} E_p u_{i,p} L_i} \ell_s \sum_{1 \leq i \leq n} E_p u_{i,p} c_{s,i} \tag{4}$$

by Supplementary Equation 2 because all the $E_p u_{i,p}$ cells belonging to every clone i contains $c_{s,i}$ copies of the ℓ_s genomic positions in s. Following the model of fractional copy numbers introduced in Methods, we know that

$$f_{s,p} = \sum_{1 \leq i \leq n} c_{s,i} u_{i,p} \tag{5}$$

and, consequently, we can rewrite Supplementary Equation 4 as the following

$$\tau_{s,p} = \frac{DR_p \ell_s f_{s,p}}{\sum\limits_{1 \leq i \leq n} u_{i,p} L_i}. \tag{6}$$

Similarly, we model the read count ν_s in the matched-normal sample as

$$\nu_s = \frac{DR\ell_s 2}{L_1} \tag{7}$$

where R is the total number of reads obtained from the matched-normal sample and because we assume that the matched-normal sample only contains a normal diploid clone with $c_{s,1} = 2$ for every cluster s. The genome lengths $L_1, \ldots, L_n$, the total numbers R, R_p of reads, and the proportions $u_{1,p}, \ldots, u_{n,p}$ are constant values in each sample p. Therefore, by combining Supplementary Equations 6 and 7 with Supplementary Equation 1 we observe that $r_{s,p}$ is directly proportional to $f_{s,p}$ by a constant $\gamma_p \in \mathrm{R}$ such that

$$f_{s,p} = \gamma_p r_{s,p} \tag{8}$$

where γ_p is the scaling factor representing all these constant factors in each sample p. More specifically, we have the following correspondence

$$\gamma_p = \frac{R \sum\limits_{1 \leq i \leq n} u_{i,p} L_i}{R_p L} \tag{9}$$

when considering that $L_1 = 2L$.

1.2 BAFs estimate the proportion of B-specific fractional copy numbers

We show that the BAF $\beta_{s,p}$ of a cluster s in a sample p estimates the proportion of the B-specific fractional copy number $f^B_{s,p}$ over the corresponding fractional copy number $f_{s,p} = f^A_{s,p} + f^B_{s,p}$. The BAF $\beta_{s,p}$ is defined as the ratio between the B-specific read count $\tau^B_{s,p}$ and the total read count $\tau_{s,p}$, i.e.

$$\beta_{s,p} = \frac{\tau^B_{s,p}}{\tau_{s,p}}. \tag{10}$$

Following the model of allele-specific fractional copy numbers introduced in Methods, we know that

$$f^B_{s,p} = \sum_{1 \leq i \leq n} b_{s,i} u_{i,p}. \tag{11}$$

As such, we model $\tau^B_{s,p}$ equivalently to $\tau_{s,p}$ in the previous Supplementary Equation 6 as

$$\tau^B_{s,p} = \frac{DR_p \ell_s f^B_{s,p}}{\sum_{1 \leq i \leq n} u_{i,p} L_i} \tag{12}$$

by considering the models of fractional copy numbers in Supplementary Equations 5 and 11. Therefore, we observe that the BAF $\beta_{s,p}$ corresponds to the ratio between $f^B_{s,p}$ and the fractional copy number $f_{s,p}$, i.e.

$$\beta_{s,p} = \frac{f^B_{s,p}}{f_{s,p}} \tag{13}$$

by combining Supplementary Equations 12 and 6 with Supplementary Equation 10.

1.3 Tumor ploidy and tumor purity are composite parameters

Tumor purity μ_p and tumor ploidy ρ_p are the two parameters used by existing methods as the coordinates of CNA inference in a tumor sample p. Tumor purity μ_p is defined as the fraction of tumor cells in p. When p comprises a single tumor clone (i.e. $i = 2$), μ_p simply corresponds to the clone proportion $u_{2,p}$. However, a tumor sample p generally contains multiple tumor clones; in this case, the tumor purity μ_p is the defined as the sum of the tumor clone proportions in p, i.e.

$$\mu_p = \sum_{i=2}^{n} u_{i,p}. \tag{14}$$

Tumor ploidy ρ_p is a measure of the actual DNA content in the genome of tumor cells and is hence used to investigate the changes in the genome length due to the effects of CNAs. As such, the tumor ploidy ρ_p is defined as the average copy number per genome position in a sample p. When p comprises a single tumor clone $i = 2$, ρ_p is simply the average copy number in the genome of the clone and we have the following

$$\rho_p = \frac{\sum_{1 \leq s \leq m} c_{s,2}}{L} = \frac{L_2}{L}. \tag{15}$$

The closer the value of ρ_p is to 2, the smaller the effect of CNAs is on the corresponding genome length L_2 with respect to the genome length L_1 of the normal cells, that is twice the reference length, i.e. $L_1 = 2L$. However, a tumor sample p generally contains multiple tumor clones; in this case, the tumor ploidy ρ_p is defined as the corresponding weighted average among the different tumor clones and we have the following

$$\rho_p = \frac{\sum_{2 \leq i \leq n} u_{i,p} L_i}{L}. \tag{16}$$

Tumor purity μ_p and tumor ploidy ρ_p are hence composite parameters: μ_p depends on the proportions of all tumor clones, and ρ_p depends both on the copy numbers and clone proportions of all tumor clones. As such, μ_p and ρ_p summarize the differences among distinct tumor clones. Existing methods consider μ_p and ρ_p as coordinates of the CNA inference because these parameters explain the direct proportionality between RDRs and fractional copy numbers. In fact, the scaling factor γ_p described above can be directly computed from μ_p and ρ_p as the following

$$\gamma_p = \frac{R}{R_p}(2(1 - \mu_p) + \rho_p) \tag{17}$$

Some existing methods do not directly consider tumor purity μ_p and tumor ploidy ρ_p but equivalently related parameters; the most common of these equivalent parameters is the haploid coverage[19,21]. The haploid coverage η_p of a sample p is defined as the average number of reads obtained from a single copy of a genomic position from all cells in p. Equivalently to Supplementary Equation 2, we can model the haploid coverage as

$$\eta_p = \frac{DR_p}{\sum_{1 \leq i \leq n} u_{i,p} L_i} \tag{18}$$

following a direct extension of the Lander-Waterman equation[22]. The existing methods that consider the haploid coverage η_p use η_p and μ_p as the coordinates for CNA inference. These coordinates are equivalently related to ρ_p and μ_p since η_p and μ_p uniquely determine ρ_p as following

$$\rho_p = \frac{DR_p}{L\eta_p} - 2(1 - \mu_p) \tag{19}$$

as D, R_p, and L are generally known.

2 HATCHet: global clustering along the genome and across samples

HATCHet clusters genomic bins with the same copy-number states along the entire genome and jointly across all samples using the estimated RDRs and BAFs. HATCHet partitions the reference genome into ζ bins of fixed size (50kb in this work but it is user adjustable). The bin size provides a lower bound on the length of CNAs that will be inferred by HATCHet; generally the bin size is chosen to be as small as possible while allowing for reliable estimation of RDRs and BAFs. As such, HATCHet aims to identify m clusters of genomic bins such that each cluster $s \subseteq \{1, \ldots, m\}$ only contains bins with the same copy-number state in every clone i, i.e. $|\{(\overline{a}_{t,i}, \overline{b}_{t,i}) : t \in s\}| = 1$ where $(\overline{a}_{t,i}, \overline{b}_{t,i})$ is the copy-number state of every genomic bin t in clone i.

We infer the m clusters of genomic bins by assuming that bins with similar values of RDR and BAF across every sample are part of the same cluster. We do this because RDR and BAF directly depend on allele-specific fractional copy numbers (Supplementary Method 1) and because we assume that two bins with the same allele-specific fractional copy numbers have the same copy-number states, similarly to existing methods[7–10,12–21,23,24].

We describe the details of the global clustering procedure in three subsections. First, we describe our direct observations from DNA sequencing data, which correspond to sequencing reads aligned to a reference genome. Second, we compute the RDR $\overline{r}_{t,p}$ and the BAF $\overline{\beta}_{t,p}$ of each bin t in every sample p from the observed read counts. Last, we identify the m clusters and we estimate the RDR $r_{s,p}$ and the BAF $\beta_{s,p}$ of each cluster s in sample p.

2.1 Direct observations from mapped sequencing reads

For every bulk tumor sample p, we consider two main observations from the sequencing reads that are mapped to a human reference genome. First, we observe the total number of reads, or read count, mapped to each bin t. We represent as $\overline{\tau}_{t,p}$ the read count of t in each tumor sample p and as $\overline{\nu}_t$ the corresponding read count of t in the matched-normal sample. HATCHet uses SAMtools[25] to obtain $\overline{\tau}_{t,p}$ and $\overline{\nu}_t$ from a file of mapped sequencing reads (BAM file).

Second, we observe the frequency $\psi_{e,p}$ of each germline heterozygous SNP e in sample p. A heterozygous germline SNP corresponds to a genomic position whose two copies have different nucleotides in the normal genome. As such, the frequency $\psi_{e,p}$ is computed as the fraction of reads in p covering one of the two variants of the genomic position e. For simplicity, we consider $\psi_{e,p}$ as the proportion of the minor variant, that is the nucleotide with the lowest read count between the two, and thus $\psi_{e,p} \in [0.0, 0.5]$. To identify potential germinal heterozygous SNPs in the matched-normal sample, HATCHet uses the combination of SAMtools[25] and BCFtools[26]. As the read counts may be affected by noise and errors, HATCHet selects the germinal heterozygous SNPs by considering a 95% confidence interval of the frequency obtained using a binomial model with a beta as prior, similarly to existing methods[17,18,27,28]. Finally, HATCHet also uses SAMtools[25] and BCFtools[26] for every tumor sample p to obtain the counts of the reads covering the variants and to compute $\psi_{e,p}$ for every germline heterozygous SNP e previously identified in the matched normal sample.

2.2 Estimating RDRs and BAFs of genomic bins

We compute two signals for each bin t from the observations from aligned DNA sequencing reads described above. These signals are the same used by existing methods for CNA identification[7–10,12–21]. The first signal is the RDR $\overline{r}_{t,p}$ of bin t in sample p that we obtain by normalizing the read count $\overline{\tau}_{t,p}$ with the corresponding read count $\overline{\nu}_t$ in the matched-normal sample, i.e. $\overline{r}_{t,p} = \frac{\overline{\tau}_{t,p}}{\overline{\nu}_t}$ (see formal definition in Supplementary Method 1). This normalization is used by existing methods[10,13,17–19] to correct for common biases that affect the read counts from the same genomic region, e.g. GC-content and mappability biases. The second signal is the BAF $\overline{\beta}_{t,p}$ that corresponds to the fraction of reads belonging to the two distinct copies, or alleles $\mathcal{A}$ and $\mathcal{B}$, of t in sample p (see formal definition in Supplementary Method 1). Since the definition of $\mathcal{A}$ and $\mathcal{B}$ is only relative to each genomic region, we define for simplicity $\overline{\beta}_{t,p}$ as the fraction of reads belonging to $\mathcal{B}$. Unfortunately, we cannot directly compute $\overline{\beta}_{t,p}$ from our observations because we do not know whether each read belongs to either $\mathcal{A}$ and $\mathcal{B}$. Therefore, we estimate $\overline{\beta}_{t,p}$ by combining the read counts from heterozygous SNPs across bin t using the standard mirrored BAF[8,29–32]: intuitively the idea is that the lower (respectively higher) read counts belong to the same allele when there is allelic imbalance. More specifically, we estimate the BAF $\overline{\beta}_{t,p}$ as the mirrored BAF $\hat{\beta}_{t,p}$ computed by averaging the frequency $\psi_{e,p}$ of every germline heterozygous SNP e within t. In fact, we assign the minor variant of every e to $\mathcal{B}$ according to the definition of $\psi_{e,p} \in [0, 0.5]$ above. In addition, we guarantee the consistency of the assignments across all samples by assigning all minor variants to $\mathcal{B}$ in a sample p and by swapping the assignment in any other sample q if the minor variants in p are clearly different from those in q (in terms of nucleotides).

The mirrored BAF $\hat{\beta}_{t,p}$ provides an unbiased estimation of the BAF $\overline{\beta}_{t,p}$ when $\overline{\beta}_{t,p} \not\approx 0.5$, but it is biased when $\overline{\beta}_{t,p} \approx 0.5$ since the variances of the frequency $\psi_{e,p}$ of every SNP e aggregate when consistently assigning the minor variants to the same allele[30]. We adopt two strategies to correct $\hat{\beta}_{t,p}$ for this bias and to estimate the BAF $\overline{\beta}_{t,p}$. The main strategy is to correct the mirrored BAFs by considering all the bins in the diploid (copy-number state $(1,1)$) or tetraploid (copy-number state $(2,2)$) clusters identified by HATCHet in the next steps. Since we know that $\beta_{s,p} = 0.5$ for the diploid/tetraploid cluster s, we proportionally correct the BAF $\hat{\beta}_{z,p}$ of every cluster z with mirrored BAF

approximately equal to 0.5 by a factor of $\frac{0.5}{\hat{\beta}_{s,p}}$, i.e. $\beta_{z,p} = \hat{\beta}_{z,p}\frac{0.5}{\hat{\beta}_{s,p}}$, where $\hat{\beta}_{s,p}, \hat{\beta}_{z,p}$ are the average mirrored BAFs for the bins within s and z, respectively. The second alternative strategy is to correct the mirrored BAF $\hat{\beta}_{t,p}$ of every bin t according to the deviation from 0.5 obtained when computing the same value for t in the matched-normal sample, similarly to previous methods[30].

2.3 Global clustering

We aim to cluster all the genomic bins across all samples based on their values of RDRs and BAFs estimated above. More specifically, we assign each bin t to a cluster s through a mapping function $h : \{1, \ldots, \zeta\} \rightarrow \{1, \ldots, m\}$ such that $h(t) = s$ when bin t is part of cluster s. A cluster s thus contains all bins which have similar values of RDR and BAF jointly across all samples, i.e.

$$\forall t, l, h(t) = h(l) = s \quad \text{if} \quad \forall p \in \{1, \ldots, k\}, \overline{r}_{t,p} \approx \overline{r}_{l,p} \wedge \overline{\beta}_{t,p} \approx \overline{\beta}_{l,p}. \tag{20}$$

We compute the clusters and we estimate their RDRs and BAFs in two steps.

We first compute the set of clusters $\{1, \ldots, m\}$ and the mapping function h through BNPY[33] which uses a bayesian nonparametric model based on the Dirichlet process to infer number m of clusters, the mapping function h, and the parameters of all the underlying distributions. To do this, we encode the RDRs and BAFs of each bin t as a vector $v_t \in \mathrm{R}^{2k}$ such that $v_t[2p] = \overline{r}_{t,p}$ and $v_t[2p+1] = \overline{\beta}_{t,p}$ for every sample p with $1 \leq p \leq k$. We apply BNPY with the same default values for all the hyperparameters and a Gaussian generative model with a diagonal covariance matrix as in THetA2[18]. Moreover, we merge clusters which have been identified with very similar values of RDR and BAF across every sample to avoid overfitting. Note that the applied clustering method is nonparametric and the number m of clusters is automatically inferred.

We then compute the RDR $r_{s,p}$ and BAF $\beta_{s,p}$ of every cluster s. In principle, every bin t in a cluster s has the same values of RDR and BAF. In practice, RDR and BAF have variance because we do not sequence an infinite number of reads. As such, we estimate the RDR $r_{s,p}$ and the BAF $\beta_{s,p}$ by averaging the corresponding values across all bins in s, i.e. $h(t) = s$, as in current CNA methods[8,10,12–20]. More specifically, we compute the RDR $r_{s,p}$ of a cluster s in sample p as the average RDR for all the bins in s, i.e.

$$r_{s,p} = \frac{1}{|\{t : h(t) = s\}|} \sum_{\forall t: h(t) = s} \overline{r}_{t,p}. \tag{21}$$

Similarly, we compute the BAF $\beta_{s,p}$ of cluster s in sample p as the average BAF for all bins in s, i.e.

$$\beta_{s,p} = \frac{1}{|\{t : h(t) = s\}|} \sum_{\forall t: h(t) = s} \overline{\beta}_{t,p}. \tag{22}$$

Notably, the estimations of $r_{s,p}$ and $\beta_{s,p}$ from this global approach are more robust to noise than the ones of local approaches; two distinct segments obtained by local clustering may have arbitrarily different values of RDR and BAF even if they have the same copy-number states in every clone. In contrast, all segments obtained by this global clustering are in the same cluster and have equal RDR and BAF if they have the same copy-number states in every clone.

3 HATCHet: estimation of fractional copy numbers

HATCHet estimates the fractional copy numbers $f_{s,p}, f^A_{s,p}, f^B_{s,p}$ from the RDR $r_{s,p}$ and BAF $\beta_{s,p}$ of every cluster s in every sample p. We do this in a two-step procedure. First, we describe a rigorous and sufficient condition to obtain the fractional copy numbers F, F^A, F^B by identifying 1 or 2 tumor-clonal clusters and their total copy numbers. Specifically, tumor-clonal clusters are clusters that have the same copy-number state in every tumor clone, i.e. a cluster s is tumor-clonal if $|\{(a_{s,2}, b_{s,2}), (a_{s,3}, b_{s,3}), \dots, (a_{s,n}, b_{s,n})\}| = 1$. Second, we design a heuristic to identify the required clusters and copy numbers from RDRs and BAFs jointly across samples. As we assume the presence of tumor-clonal clusters, we design this heuristic assuming either there is or not a WGD in all tumor clones. This assumption is consistent with previous pan-cancer studies[7,34–36].

3.1 Obtaining fractional copy numbers from tumor-clonal clusters

We describe a rigorous and sufficient condition to obtain the fractional copy numbers F, F^A, F^B given the total copy numbers of 1 or 2 tumor-clonal clusters. The fractional copy number $f_{s,p}$ of a cluster s in every sample p is directly proportional to the RDR $r_{s,p}$ by a scaling factor γ_p (Supplementary Method 1). Therefore, we can obtain all fractional copy numbers F by identifying γ_p of every sample p. To do this, the following theorem states a sufficient condition to obtain γ_p of every sample p based on the identification of either one tumor-clonal cluster with total copy number of 2 in every clone or two tumor-clonal clusters with total copy numbers $c_{s,i} = \omega_s, c_{z,i} = \omega_z$ in every tumor clone i.

Theorem 1. *Let $r_{s,p}$ be the RDR of every cluster s in a sample p.*

1. *If s is a tumor-clonal cluster with total copy number $c_{s,i} = 2$ in every tumor clone i then for every sample p*

$$\gamma_p = \frac{2}{r_{s,p}}.$$

2. *If s, z are two tumor-clonal clusters with total copy numbers $c_{s,i} = \omega_s, c_{z,i} = \omega_z$ in every tumor clone i, respectively, s.t. $\omega_s \neq \omega_z$ and $r_{s,p}(\omega_z - 2) \neq r_{z,p}(\omega_s - 2)$, then for every sample p*

$$\gamma_p = \frac{2\omega_z - 2\omega_s}{r_{s,p}(\omega_z - 2) + r_{z,p}(2 - \omega_s)}.$$

Proof. In the following, we prove each statement separately.

1. We know that $c_{s,i} = 2$ for every tumor clone i since s is a tumor-clonal cluster with copy number equal to 2. From Supplementary Equation 5, it follows that $f_{s,p} = 2$ in every sample p. Moreover, we know that $f_{s,p} = \gamma_p r_{s,p}$ by Supplementary Equation 8. Hence, $\gamma_p = \frac{2}{r_{s,p}}$ where the RDR $r_{s,p}$ is known.

2. We know that $c_{s,i} = \omega_s$ and $c_{z,i} = \omega_z$ for every tumor clone i since s, z are tumor-clonal clusters with total copy numbers equal to ω_s, ω_z, respectively. From Supplementary Equation 5, it follows that $f_{s,p} = 2(1 - \tau_p) + \omega_s \tau_p$ and $f_{z,p} = 2(1 - \tau_p) + \omega_z \tau_p$ in every sample p with tumor purity μ_p. Moreover, we know that $f_{s,p} = \gamma_p r_{s,p}$ by Supplementary Equation 8. Hence, when $\omega_s \neq \omega_z$ and $r_{s,p}(\omega_z - 2) \neq r_{z,p}(\omega_s - 2)$ there always exists

a unique solution for the unknown scale factor γ_p and purity μ_p in the following system of two equations for every sample p

$$\begin{cases} f_{s,p} = 2(1-\mu_p) + \omega_s\mu_p \\ f_{z,p} = 2(1-\mu_p) + \omega_z\mu_p \end{cases} = \begin{cases} \gamma_p r_{s,p} = 2(1-\mu_p) + \omega_s\mu_p \\ \gamma_p r_{z,p} = 2(1-\mu_p) + \omega_z\mu_p \end{cases} = \tag{23}$$

such that $\gamma_p = \frac{2\omega_z - 2\omega_s}{r_{s,p}(\omega_z-2)+r_{z,p}(2-\omega_s)}$ and $\mu_p = \frac{\gamma_p r_{s,p}-2}{\omega_s-2} = \frac{\gamma_p r_{z,p}-2}{\omega_z-2}$ where the RDRs $r_{s,p}, r_{z,p}$ are known and the copy numbers ω_s, ω_z are given.

□

Note that the second condition of Theorem 1 is also a sufficient condition for obtaining the purity μ_p of every sample p.

Corollary 2. *Let $r_{s,p}$ be the RDR of a cluster s in a sample p. If s, z are two tumor-clonal clusters with total copy numbers ω_s, ω_z, respectively, s.t. $\omega_s \neq \omega_z$ and $r_{s,p}(\omega_z - 2) \neq r_{z,p}(\omega_s - 2)$, then for every sample p*

$$\mu_p = \frac{\gamma_p r_{s,p} - 2}{\omega_s - 2} = \frac{\gamma_p r_{z,p} - 2}{\omega_z - 2}.$$

Theorem 1 provides a sufficient condition to obtain F. As such, we can easily obtain the allele-specific fractional copy numbers F^A, F^B by using the BAF $\beta_{s,p}$ of every cluster s. More specifically, we apply Supplementary Equation 13 to obtain the $\mathcal{B}$-specific fractional copy number $f^B_{s,p}$ for every cluster s in sample p by using using the inferred fractional copy number $f_{s,p}$ and the BAF $\beta_{s,p}$. We hence infer the $\mathcal{A}$-specific fractional copy number $f^A_{s,p}$ for each s in p from the difference $f_{s,p} - f^B_{s,p}$.

3.2 Identification of tumor-clonal clusters of genomic regions

We design a heuristic to identify the needed tumor-clonal clusters and their total copy numbers to apply Theorem 1 and obtain the fractional copy numbers F. We distinguish the cases when a WGD occurs or not. When there is no WGD, we expect there is a cluster s including a large amount of bins with copy-number state $(1, 1)$ as these are diploid bins which have not been affected by CNAs. As $f_{s,p} = 2$ and $f^B_{s,p} = 1$ by Supplementary Equations 5 and 11, the expected value of BAF $\beta_{s,p}$ is 0.5 in every sample p by Supplementary Equation 13. When there is a WGD, we expect there is a cluster s including a large amount of bins with copy-number state $(2, 2)$ as these were the diploid bins which have not been affected by CNAs but a WGD. The expected value of $\beta_{s,p}$ is still 0.5 in every sample p because $f_{s,p} = 2(1-\mu_p) + 4\mu_p$ and $f^B_{s,p} = 1 - \mu_p + 2\mu_p$ by Supplementary Equations 5 and 11, and the ratio of these values in Supplementary Equation 13 is equal to 0.5 for any value of tumor purity μ_p. Therefore, HATCHet aims to identify the cluster s with a base state (θ, θ), that is a copy-number state either equal to $(1, 1)$ or $(2, 2)$ without a WGD or with a WGD, respectively. More specifically, we denote by ℓ_s the size of every cluster s (i.e. the corresponding number of genomic bins) and HATCHet identifies s as the largest cluster with an estimated BAF $\beta_{s,p}$ approximately equal to 0.5 in every sample p, i.e.

$$s = \underset{z:\forall p, \beta_{z,p} \approx 0.5}{\operatorname{argmax}} \ \ell_z. \tag{24}$$

The identification of the cluster s with base state $(1,1)$ is sufficient to apply Theorem 1 when there is no WGD because the corresponding total copy number is equal to 2 in every clone, i.e. $c_{s,i} = 2$ for every $i \in \{1,\ldots,n\}$. However, when there is a WGD, the identification of the cluster s with base state $(2,2)$ is not sufficient to apply Theorem 1 as the total copy number is 4 for every tumor clone, i.e. $c_{s,i} = 4$ for every $i \in \{2,\ldots,n\}$. We thus need to identify another tumor-clonal cluster z and its total copy number ω_z in this case. As tumor-clonal clusters preserve their relative values of RDR and BAF in every sample (i.e. they preserve their relative position in each BAF-RDR plot, Supplementary Fig. 25), we restrict the candidates to clusters which do not change their relative RDR and BAF across the samples. For example, we discard candidate clusters that have a RDR lower than the one of the cluster s with base state $(2,2)$ in one sample and higher in another sample: a cluster z with $r_{z,p} \simeq r_{s,p}$ in sample p and $r_{z,q} < r_{s,q}$ or $r_{z,q} > r_{s,q}$ in another sample q cannot be a candidate tumor-clonal cluster. A similar change in terms of BAF is also not indicative for a tumor-clonal cluster. As a further example, we discard candidate clusters that clearly change the relative value of BAF across samples with respect to the lowest value of BAF within each sample, if the lowest value is given by a large cluster with the same relative RDR with respect to s in every sample. Moreover, we focus on clusters whose copy-number states correspond to common CNAs as single amplifications and deletions[34]. As such, we aim to identify tumor-clonal clusters whose copy-number states are either equal to $(2,1)$ or $(3,2)$ (and symmetrically to $(1,2)$ or $(2,3)$) for CNAs occurred after WGD, or either equal to $(2,0)$ or $(4,2)$ (and symmetrically to $(0,2)$ or $(2,4)$) for CNAs occurred before WGD. As we expect some of these tumor-clonal clusters to be present in every tumor clone for a large portion of the genome, we additionally restrict the candidates to sufficiently-large clusters that are present in multiple chromosomes, especially when the tumor is characterized by many CNAs.

We can generally identify several candidate tumor-clonal clusters and we also need to know their total copy number to apply Theorem 1 when there is a WGD. To find the best candidate and its total copy number, the key idea of our heuristic is based on Corollary 2: since we have already identified the cluster s with base state $(2,2)$, the identification of another tumor-clonal cluster z and its copy-number state (α,ι) correspondingly provides the associated values of the scaling factor $\tilde{\gamma}_{p,z,\alpha,\iota}$ from Theorem 1 and tumor purity $\tilde{\tau}_{p,z,\alpha,\iota}$ from Corollary 2 for every sample p. We thus consider the values of $\tilde{\gamma}_{p,z,\alpha,\iota}$ and $\tilde{\tau}_{p,z,\alpha,\iota}$ associated to each choice of z and (α,ι) to select the best candidate and its total copy number. More specifically, we use $\tilde{\gamma}_{p,z,\alpha,\iota}$ and $\tilde{\tau}_{p,z,\alpha,\iota}$ to identify, in addition to z, any other associated tumor-clonal cluster $\mathfrak{z}$ with copy-number state $(\overline{\alpha},\overline{\iota})$. In fact, given $\tilde{\gamma}_{p,z,\alpha,\iota}$ and $\tilde{\tau}_{p,z,\alpha,\iota}$, a tumor-clonal cluster $\mathfrak{z}$ with copy-number state $(\overline{\alpha},\overline{\iota})$ has RDR corresponding to

$$r_{\mathfrak{z},p} \approx \frac{2(1-\tilde{\tau}_{p,z,\alpha,\iota}) + (\overline{\alpha}+\overline{\iota})\tilde{\tau}_{p,z,\alpha,\iota}}{\tilde{\gamma}_{p,z,\alpha,\iota}} \tag{25}$$

by Supplementary Equations 5 and 8, and BAF corresponding to

$$\beta_{\mathfrak{z},p} \approx \frac{1-\tilde{\tau}_{p,z,\alpha,\iota} + \overline{\iota}\tilde{\tau}_{p,z,\alpha,\iota}}{2(1-\tilde{\tau}_{p,z,\alpha,\iota}) + (\overline{\alpha}+\overline{\iota})\tilde{\tau}_{p,z,\alpha,\iota}} \tag{26}$$

by Supplementary Equations 5, 11, and 13. Therefore, for every candidate clonal cluster z and every possible copy-

number state (α, ι), we obtain a set $\Theta_{z,\alpha,\iota}$ containing all the associated tumor-clonal clusters such that

$$\Theta_{z,\alpha,\iota} = \{\mathfrak{z} \mid \mathfrak{z} \in \{1, \dots, m\}, \overline{\alpha} \in \mathbb{N}, \overline{\iota} \in \mathbb{N} : \\ \forall p \in \{1, \dots, k\}, \beta_{\mathfrak{z},p} \approx \frac{1 - \tilde{\tau}_{p,z,\alpha,\iota} + \overline{\iota}\tilde{\tau}_{p,z,\alpha,\iota}}{2(1 - \tilde{\tau}_{p,z,\alpha,\iota}) + (\overline{\alpha} + \overline{\iota})\tilde{\tau}_{p,z,\alpha,\iota}} \wedge r_{\mathfrak{z},p} \approx \frac{2(1 - \tilde{\tau}_{p,z,\alpha,\iota}) + (\overline{\alpha} + \overline{\iota})\tilde{\tau}_{p,z,\alpha,\iota}}{\tilde{\gamma}_p}\}. \tag{27}$$

We use $\Theta_{z,\alpha,\iota}$ to evaluate each candidate clonal cluster z and its copy-number state (α, ι). We expect tumor-clonal clusters to correspond to a large fraction of the genome for tumor clones from the same patient as they accumulate common CNAs during tumor evolution (similarly to existing methods[8,9]). Therefore, we choose the best candidate tumor-clonal cluster z^* and its total copy-number γ_{z^*} as the candidate cluster z associated with the largest set $\Theta_{z,\alpha,\iota}$, i.e.

$$z^*, \gamma_{z^*} = \underset{\substack{z \in \{1,\dots,m\}, \alpha \in \mathbb{N}, \iota \in \mathbb{N}: \\ \gamma_{z^*} = \alpha + \iota}}{\operatorname{argmax}} \sum_{\mathfrak{z} \in \Theta_{z,\alpha,\iota}} \ell_{\mathfrak{z}}. \tag{28}$$

Moreover, as said before, we restrict the possible copy-number states (α, ι) of any candidate cluster z to $\{(2,1), (1,2), (0,2), (2,0)\}$ for a cluster z with $r_{z,p} < r_{s,p}$, whereas we consider $\{(3,2), (2,3), (4,2), (2,4)\}$ for a cluster z with $r_{z,p} > r_{s,p}$. We thus apply Theorem 1 when there is a WGD by considering the cluster s with total copy number $\omega_s = 4$ and cluster z^* with total copy number $\omega_z = \gamma_{z^*}$.

4 HATCHet: constrained and distance-based allele-specific copy-number factorization

We aim to infer the allele-specific copy numbers A, B and the clone proportions U from the estimated allele-specific fractional copy numbers F^A, F^B. We first provide details for the constrained and distance-based variant D-CACF formulation of ACF problem that we introduced to deal with the practical issues related to DNA sequencing reads described in Methods. We next design a coordinate-descent (CD) algorithm and an integer-linear programming (ILP) formulation to solve those. The ILP formulation solves exactly the problem but does not scale, while the CD algorithm does not guarantee the optimality of the solution but it is very efficient. HATCHet uses CD as the main method to infer A, B, U but it can also use ILP to evaluate the gap between the solution found by CD and the optimum, and to possibly improve the solution.

4.1 Problem formulation

Let F^A and F^B be the inferred $m \times k$ matrices of allele-specific fractional copy numbers. We assume n is the number of clones. According to the ideal problem formulation of ACF in Methods, we aim to factorize F^A, F^B into two $m \times n$ matrices A, B of allele-specific copy numbers and a $n \times k$ matrix U of clone proportions such that $F^A = AU$ and $F^B = BU$. ACF is an underdetermined problem with many degenerate solutions. To avoid degenerate cases, we constrain the factorization by assuming c_{max} is the maximum total copy number. Moreover, since the capability to infer the presence of clones in certain proportions is limited by our observations and corresponding noise, we also introduce a minimum clone proportion u_{min} for all tumor clones present in a sample. Therefore, we have the following constrained variant of the problem.

Problem 1 (Constrained Allele-specific Copy-number Factorization (CACF) problem)**.** *Given two $m \times k$ matrices of allele-specific fractional copy numbers F^A, F^B, a number of clones n, a maximum total copy number c_{max}, and a minimum clone proportion u_{min}, find two $m \times n$ matrices of allele-specific copy numbers $A = [a_{s,i}]$, $B = [b_{s,i}]$, and a $n \times k$ matrix of clone proportions $U = [u_{i,p}]$ such that $F^A = AU$, $F^B = BU$, $a_{s,i} + b_{s,i} \leq c_{max}$, and $u_{i,p} \geq u_{min}$ or $u_{i,p} = 0$.*

The hardness of CACF is an open question, but we suspect the problem to be NP-hard as closely-related problems are computationally hard as well[37,38]. We also do not observe F^A, F^B directly but we estimate these (Supplementary Method 3). As such, measurement errors may affect the values of A, B, U and even their existence. For this reason, we introduce a distance-based version of CACF. More specifically, we define the distance between the estimated allele-specific fractional copy numbers F^A, F^B and the correspondingly inferred fractional copy numbers AU, BU as $D = \|F^A - AU\| + \|F^B - BU\|$ such that

$$\|F^A - AU\| = \sum_{1 \leq s \leq m} \ell_s \sum_{1 \leq p \leq k} |f^A_{s,p} - \sum_{1 \leq i \leq n} a_{s,i} u_{i,p}| \tag{29}$$

and

$$\|F^B - BU\| = \sum_{1 \leq s \leq m} \ell_s \sum_{1 \leq p \leq k} |f^B_{s,p} - \sum_{1 \leq i \leq n} b_{s,i} u_{i,p}| \tag{30}$$

where the distances are correspondingly weighted by the size ℓ_s of each cluster s. We thus have the following distance-based variant of the problem.

Problem 2 (Distance-based CACF (D-CACF) problem)**.** *Given the allele-specific fractional copy numbers F^A, F^B, a number of clones n, a maximum total copy number c_{max}, and a minimum clone proportion u_{min}, find the allele-specific copy numbers $A = [a_{s,i}]$, $B = [b_{s,i}]$, and the clone proportions $U = [u_{i,p}]$ for n clones such that $\|F^A - AU\| + \|F^B - BU\|$ is minimum, $c^A_{s,i} + c^B_{s,i} \leq c_{max}$, and $u_{i,p} \geq u_{min}$ or $u_{i,p} = 0$.*

We previously showed[23,24] that constraints involving the evolution of CNAs may improve the results of a related factorization problem. As such, we also consider a variant of D-CACF where we impose an evolutionary relationship between the tumor clones by asking that the same allele of every cluster s cannot be simultaneously amplified and deleted in distinct clones. More specifically, we have for every cluster s that

$$\forall i \in \{1, \ldots, n\}\ a_{s,i} \geq \theta \veebar \forall i \in \{1, \ldots, n\}\ a_{s,i} \leq \theta \tag{31}$$

$$\forall i \in \{1, \ldots, n\}\ b_{s,i} \geq \theta \veebar \forall i \in \{1, \ldots, n\}\ b_{s,i} \leq \theta. \tag{32}$$

such that $\theta = 2$ or $\theta = 1$ when there is or not a WGD, respectively. We thus consider a variant of D-CACF which also includes these constraints. These are optional and less restrictive constraints than the ones usually applied in existing methods[9,13,14,17–19] which, for example, use one or more of the following assumptions:

1. The same allele-specific copy number $a_{s,i}, a_{s,j}$ or $b_{s,i}, b_{s,j}$ of each cluster s in different tumor clones i, j have a difference of at most 1, i.e. $|a_{s,i} - a_{s,j}| \leq 1$ and $|b_{s,i} - b_{s,j}| \leq 1$[9].

2. The total copy number $c_{s,i}$ of every cluster s is either $c_{s,i} \leq 2$ or $c_{s,i} \geq 2$ for every tumor clone i[17,18].

3. There is at most one copy-number state different than $(1,1)$ per each cluster s, i.e. $|\{(a_{s,i}, b_{s,i}) : 1 \leq s \leq m, 1 \leq i \leq n, (a_{s,i}, b_{s,i}) \neq (1,1)\}| = 1$ for every cluster s[13,14].

We thus have the following final variant of the problem formulation for D-CACF.

Problem 3 (Distance-based Constrained Allele-specific Copy-number Factorization (D-CACF) problem)**.** *Given the allele-specific fractional copy numbers F^A and F^B, a number n of clones, a maximum total copy number c_{max}, a minimum clone proportion u_{min}, and a constant value $\theta \in \{1,2\}$, find allele-specific copy numbers $A = [a_{s,i}]$, $B = [b_{s,i}]$, and clone proportions $U = [u_{i,p}]$ such that: the distance $D = \|F^A - AU\| + \|F^B - BU\|$ is minimum; $a_{s,i} + b_{s,i} \leq c_{max}$ for every cluster s and clone i; either $u_{i,p} \geq u_{min}$ or $u_{i,p} = 0$ for every clone i and sample p; for every cluster s, either $a_{s,i} \geq \theta$* or *$a_{s,i} \leq \theta$ for all clones i; for every cluster s, either $b_{s,i} \geq \theta$* or *$b_{s,i} \leq \theta$ for all clones i.*

4.2 Coordinate-descent algorithm

In this section we design a coordinate-descent algorithm (CD) for D-CACF. The coordinate-descent procedure of this algorithm is inspired to the method that we introduced[23,24] for a related factorization problem. Let F^A, F^B be the given matrices of allele-specific fractional copy numbers, n the given number of clones, and $c_{\max}$, $u_{\min}$ the given maximum total copy number and minimum clone proportion, respectively. We split the variables of D-CACF and we obtain two subproblems where either matrices A, B or matrix U are fixed. Both the subproblems have the same objective of D-CACF, that is to minimize the distance $\|F^A - AU\| + \|F^B - BU\|$. For simplicity, we denote as $d(A, B, U)$ the distance $\|F^A - AU\| + \|F^B - BU\|$ for the given F^A and F^B. The CD algorithm is iterative and each iteration t consists of two steps: the C-step and the the U-step. In the C-step, we are given a matrix of clone proportions U_{t-1} and we seek for two matrices of allele-specific copy numbers A_t, B_t minimizing $d(A_t, B_t, U_{t-1})$ such $a_{s,i} + b_{s,i} \leq c_{\max}$ for every cluster s in clone i and such that constraints in Supplementary Equations 31 and 32 hold. In the U-step, we are given two matrices of allele-specific copy numbers A_t, B_t and we seek for a matrix of clone proportions U_t minimizing $d(A_t, B_t, U_t)$ such that $u_{i,p} \geq u_{\min}$ or $u_{i,p} = 0$ for every clone i in sample p.

To account for local optima, we restart the iterative procedure initiating from Q different usage matrices $U_{0,1}, \ldots, U_{0,Q}$ that we randomly generate in a sparse way using random-number partitions[39]. The iterative process yields a sequence of non-increasing values of the distance from each restart q, i.e. $d(A_{t,q}, B_{t,q}, U_{t,q}) \leq d(A_{t+1,q}, B_{t+1,q}, U_{t,q})$ in the C-step and $d(A_{t+1,q}, B_{t+1,q}, U_{t,q}) \leq d(A_{t+1,q}, B_{t+1,q}, U_{t+1,q})$ in the U-step of every iteration t. This is because we can choose $(A_{t,q}, B_{t,q}) = (A_{t+1,q}, B_{t+1,q})$ and $U_{t,q} = U_{t+1,q}$. Therefore, we iterate until convergence.

We first describe an ILP formulation for solving the C-step and next an ILP formulation for solving the U-step. In both steps, we are given two $m \times k$ matrices of allele-specific fractional copy numbers $F^A = [f^A_{s,p}]$ and $F^B = [f^B_{s,p}]$ such that $f^A_{s,p}$ and $f^B_{s,p}$ are the corresponding allele-specific fractional copy numbers of every cluster s in each sample p. In addition, we are given the number n of clones, the maximum total copy number $c_{\max}$, the minimum clone proportion $u_{\min}$, and a constant value $\theta \in \{1,2\}$. At every step, the variables of each ILP are initialized using the

solutions at the previous step to guarantee the monotonicity of the objective function.

C-step We design an ILP formulation for solving the C-step consisting of $O(mk+nk+mn)$ variables and $O(mk+nk+mn)$ constraints. Let $U = [u_{i,p}]$ be the given $n \times k$ matrix of clone proportions such that $u_{i,p}$ is the clone proportion of clone i in sample p. We seek to find two matrices of allele-specific copy numbers A and B which minimize the distance $d(A, B, U)$. As such, we introduce two integer variables $a_{s,i} \in \{0, \ldots, c_{\max}\}$ and $b_{s,i} \in \{0, \ldots, c_{\max}\}$ for every cluster s in clone i and we add the following constraints to require the total copy number to be non-greater than $c_{\max}$.

$$a_{s,i} + b_{s,i} \leq c_{\max} \qquad 1 \leq s \leq m, 1 \leq i \leq n \tag{33}$$

Next, we encode the optional allele-specific constraints concerning CNA evolution in Supplementary Equations 31 and 32. We consider a constant $\theta \in \{1, 2\}$ representing the allele-specific copy number of clusters with a base state, i.e. $\theta = 1$ if no WGD or $\theta = 2$ if there is a WGD. We model these constraints by introducing two binary variables $v_s^A, v_s^B \in \{0, 1\}$ for every cluster s and the following linear constrains, as $c_{\max}$ is the maximum value for both $a_{s,i}$ and $b_{s,i}$ for every clone i.

$$a_{s,i} \leq c_{\max} v_s^A + \theta - \theta v_s^A \qquad 1 \leq s \leq m, 1 \leq i \leq n \tag{34}$$

$$a_{s,i} \geq \theta v_s^A \qquad 1 \leq s \leq m, 1 \leq i \leq n \tag{35}$$

$$b_{s,i} \leq c_{\max} v_s^B + \theta - \theta v_s^B \qquad 1 \leq s \leq m, 1 \leq i \leq n \tag{36}$$

$$b_{s,i} \geq \theta v_s^B \qquad 1 \leq s \leq m, 1 \leq i \leq n \tag{37}$$

Last, we encode the objective function as the distance $d(A, B, U)$. The allele-specific weighted sums of copy numbers $\sum_{1 \leq i \leq n} a_{s,i} u_{i,p}$ and $\sum_{1 \leq i \leq n} b_{s,i} u_{i,p}$ correspond to our estimation of the allele-specific fractional copy numbers $f_{s,p}^A, f_{s,p}^B$ for each cluster s in samples p. As such, we model the absolute differences $|f_{s,p}^A - \sum_{1 \leq i \leq n} a_{s,i} u_{i,p}|$ and $|f_{s,p}^B - \sum_{1 \leq i \leq n} b_{s,i} u_{i,p}|$ for each cluster s in sample p by introducing the variables $y_{s,p}^A$ and $y_{s,p}^B$ and adding the following constraints.

$$y_{s,p}^A \geq f_{s,p}^A - \sum_{1 \leq i \leq n} a_{s,i} u_{i,p} \qquad 1 \leq s \leq m, 1 \leq p \leq k \tag{38}$$

$$y_{s,p}^A \geq \sum_{1 \leq i \leq n} a_{s,i} u_{i,p} - f_{s,p}^A \qquad 1 \leq s \leq m, 1 \leq p \leq k \tag{39}$$

$$y_{s,p}^B \geq f_{s,p}^B - \sum_{1 \leq i \leq n} b_{s,i} u_{i,p} \qquad 1 \leq s \leq m, 1 \leq p \leq k \tag{40}$$

$$y_{s,p}^B \geq \sum_{1 \leq i \leq n} b_{s,i} u_{i,p} - f_{s,p}^B \qquad 1 \leq s \leq m, 1 \leq p \leq k \tag{41}$$

The distance $d(A, B, U)$ hence corresponds to the sum of all variables $y_{s,p}^A$ and $y_{s,p}^B$ weighted by the size ℓ_s of the corresponding cluster s, i.e.

$$\sum_{1 \leq s \leq m} \ell_s \sum_{1 \leq p \leq k} y_{s,p}^A + y_{s,p}^B. \tag{42}$$

We have the following ILP:

$$\min: \sum_{1 \le s \le m} \ell_s \sum_{1 \le p \le k} y^A_{s,p} + y^B_{s,p} \tag{43}$$

$$\text{s.t. } (33), (34), (35), (36), (37), (38), (39), (40), \text{ and } (41).$$

U-step We design an ILP formulation for solving the U-step consisting of $O(mk + nk)$ variables and $O(mk + nk)$ constraints. Let $A = [a_{s,i}]$ and $B = [b_{s,i}]$ be the given $m \times n$ matrices of allele-specific copy numbers such that $a_{s,i}$ and $b_{s,i}$ are the allele-specific copy-numbers of each cluster s in each sample p. We seek to find a $n \times m$ matrix U of clone proportions to minimize the distance $d(A, B, U)$. As such, we introduce a variable $u_{i,p} \in [0, 1]$ for every clone i in sample p and we add the following constraints to force the clone proportions in every sample to sum up to 1.

$$\sum_{1 \le i \le n} u_{i,p} = 1 \qquad 1 \le p \le k \tag{44}$$

Moreover, we request that the clone proportion $u_{i,p}$ of every clone i in sample p is either greater than u_{min} when i is present in p, i.e. $u_{i,p} \ge u_{\text{min}}$, or is equal to 0 when i is not present in p, i.e. $u_{i,p} = 0$. To model the two cases, we introduce a binary variable $x_{i,p} \in \{0, 1\}$ to indicate whether a clone i is present or not in sample p, i.e. $u_{i,p} > 0$ or $u_{i,p} = 0$, respectively. Hence, we correspondingly model these cases with the following constraints.

$$u_{i,p} + 1 - x_{i,p} \ge u_{\text{min}} \qquad 1 \le i \le n, 1 \le p \le k \tag{45}$$

$$u_{i,p} \le x_{i,p} \qquad 1 \le i \le n, 1 \le p \le k \tag{46}$$

We encode the objective function as the distance $d(A, B, U)$ similarly to the C-step by introducing the variables $y^A_{s,p}$ and $y^B_{s,p}$ for each cluster s in sample p and the constraints (38), (39), (40) and (41). Hence, we have the following ILP:

$$\min: \sum_{1 \le s \le m} \ell_s \sum_{1 \le p \le k} y^A_{s,p} + y^B_{s,p} \tag{47}$$

$$\text{s.t. } (44), (45), (46), (38), (39), (40) \text{ and } (41)$$

4.3 Integer-linear programming formulation

In this section we design an integer-linear programming (ILP) formulation for D-CACF consisting of $O(mk + nk + mn)$ variables and $O(mk + nk + mn)$ constraints. We mainly use this method to improve the solution found by the CD algorithm and to evaluate the gap between its objective value and the optimum. In fact, the variables of this ILP can be initialized using the values of a known solution and the computation of the optimum starts from that point. Let $F^A = [f^A_{s,p}]$ and $F^B = [f^B_{s,p}]$ be the given $m \times k$ matrices of the allele-specific fractional copy numbers for every cluster s in each sample p. In addition, let n be the given number of clones, c_{max} the given maximum total copy number, and u_{min} the given minimum clone proportion. We seek to find two $m \times n$ matrices of allele-specific copy

numbers A, B and a $n \times m$ matrix U of clone proportions which minimize the distance $\|F^A - AU\| + \|F^B - BU\|$, i.e. $d(A, B, U)$.

Similarly to the C-step above, we introduce integer variables $a_{s,i} \in \{0, \ldots, c_{\text{max}}\}$ and $b_{s,i} \in \{0, \ldots, c_{\text{max}}\}$ for each cluster s in clone i and the constraints (33) to encode the allele-specific copy numbers. We additionally introduce binary variables $\bar{a}_{s,i,j}$ and $\bar{b}_{s,i,j}$ for $j \in \{1, \ldots, \Pi\}$ with $\Pi = \lceil \log_2 c_{\text{max}} \rceil + 1$ to express the binary representation of $a_{s,i}$ and $b_{s,i}$ for each cluster s in clone i through the following constraints.

$$a_{s,i} = \sum_{j=0}^{\Pi} 2^j \bar{a}_{s,i,j} \qquad 1 \leq s \leq m, 1 \leq i \leq n \tag{48}$$

$$b_{s,i} = \sum_{j=0}^{\Pi} 2^j \bar{b}_{s,i,j} \qquad 1 \leq s \leq m, 1 \leq i \leq n \tag{49}$$

Similarly to the U-step above, we also introduce the variable $u_{i,p} \in [0, 1]$ and the binary variable $x_{i,p} \in \{0, 1\}$ together with constraints (44), (45), and (46) to model the clone proportion $u_{i,p}$ for each clone i in sample p. As for the CD algorithm above, we also introduce the constrains (34), (35), (36), and (37) to model the optional allele-specific constraints concerning CNA evolution.

We cannot model the distance $d(A, B, U)$ as for the C-step and the U-step because here $d(A, B, U)$ is nonlinear. However, we can represent the allele-specific weighted sums of copy numbers as $\sum_{1 \leq i \leq n} \sum_{1 \leq j \leq \Pi} 2^j \bar{a}_{s,i,j} u_{i,p}$ and $\sum_{1 \leq i \leq n} \sum_{1 \leq j \leq \Pi} 2^j \bar{b}_{s,i,j} u_{i,p}$ using the binary representation of the allele-specific copy numbers. Therefore, we model the internal products $\bar{a}_{s,i,j} u_{i,p}$ and $\bar{b}_{s,i,j} u_{i,p}$ using the variables $z^A_{s,i,j,p}, z^B_{s,i,j,p} \in [0, 1]$ and the following constraints.

$$z^A_{s,i,j,p} \leq \bar{a}_{s,i,j} \qquad 1 \leq s \leq m, 1 \leq i \leq n, 1 \leq j \leq \Pi, 1 \leq p \leq k \tag{50}$$

$$z^A_{s,i,j,p} \leq u_{i,p} \qquad 1 \leq s \leq m, 1 \leq i \leq n, 1 \leq j \leq \Pi, 1 \leq p \leq k \tag{51}$$

$$z^A_{s,i,j,p} \geq \bar{a}_{s,i,j} + u_{i,p} - 1 \qquad 1 \leq s \leq m, 1 \leq i \leq n, 1 \leq j \leq \Pi, 1 \leq p \leq k \tag{52}$$

$$z^B_{s,i,j,p} \leq \bar{b}_{s,i,j} \qquad 1 \leq s \leq m, 1 \leq i \leq n, 1 \leq j \leq \Pi, 1 \leq p \leq k \tag{53}$$

$$z^B_{s,i,j,p} \leq u_{i,p} \qquad 1 \leq s \leq m, 1 \leq i \leq n, 1 \leq j \leq \Pi, 1 \leq p \leq k \tag{54}$$

$$z^B_{s,i,j,p} \geq \bar{b}_{s,i,j} + u_{i,p} - 1 \qquad 1 \leq s \leq m, 1 \leq i \leq n, 1 \leq j \leq \Pi, 1 \leq p \leq k \tag{55}$$

Similarly to the C-step and the U-step above, we can now model the absolute differences $|f^A_{s,p} - \sum_{1 \leq i \leq n} c^A_{s,i} u_{i,p}|$ and $|f^B_{s,p} - \sum_{1 \leq i \leq n} c^B_{s,i} u_{i,p}|$ for each cluster s in sample p by introducing the variables $y^A_{s,p}$ and $y^B_{s,p}$ and adding the

following constraints.

$$y^A_{s,p} \geq f^A_{s,p} - \sum_{1\leq i\leq n} \sum_{1\leq j\leq \Pi} 2^j z^A_{s,i,j,p} \qquad 1 \leq s \leq m, 1 \leq p \leq k \tag{56}$$

$$y^A_{s,p} \geq \sum_{1\leq i\leq n} \sum_{1\leq j\leq \Pi} 2^j z^A_{s,i,j,p} - f^A_{s,p} \qquad 1 \leq s \leq m, 1 \leq p \leq k \tag{57}$$

$$y^B_{s,p} \geq f^B_{s,p} - \sum_{1\leq i\leq n} \sum_{1\leq j\leq \Pi} 2^j z^B_{s,i,j,p} \qquad 1 \leq s \leq m, 1 \leq p \leq k \tag{58}$$

$$y^B_{s,p} \geq \sum_{1\leq i\leq n} \sum_{1\leq j\leq \Pi} 2^j z^B_{s,i,j,p} - f^B_{s,p} \qquad 1 \leq s \leq m, 1 \leq p \leq k \tag{59}$$

The distance $d(A, B, U)$ hence corresponds to the sum of all variables $y^A_{s,p}$ and $y^B_{s,p}$, and we have the following ILP:

$$\min : \sum_{1\leq s\leq m} \ell_s \sum_{1\leq p\leq k} y^A_{s,p} + y^B_{s,p} \tag{60}$$

s.t. (33), (44), (45), (46), (34), (35), (36), (37), (48), (49),

(50), (51), (52), (53), (54), (55), (56), (57), (58) and (59). (61)

Finally, we observe that this ILP is characterized by symmetries of the solutions since the clones can be permuted without changing the objective value of the distance $d(A, B, U)$. Therefore, we introduce the following constraints to break symmetries by imposing an order on the clones.

$$\sum_{1\leq p\leq k} u_{i,p} + \sum_{1\leq s\leq m} s^2 a_{s,i} + s b_{s,i} \leq \sum_{1\leq p\leq k} u_{i+1,p} + \sum_{1\leq s\leq m} s^2 a_{s,i+1} + s b_{s,i+1} \qquad 1 \leq i < n \tag{62}$$

These constraints do not break all symmetries and solutions with the same objective value may be still characterized by the same order of the clones. However, these constraints typically have a substantial impact in practice[40].

5 HATCHet: joint selection of number of clones and WGD

In this section, we describe the details of the model-selection criterion used by HATCHet to jointly infer the number n of clones and the occurrence of a WGD. Our model selection procedure consists of two steps. First, we observe that the distance D is a monotonically decreasing function of the number n of clones[23,24]. Under the assumption that no WGD has occurred, we compute the distance D using the no-WGD-scaled F_A and F_B and find the value n^*_2 where the decrease in D first becomes small using a standard elbow criterion[41–43], which we describe below in this section. Similarly under the assumption that a WGD occurred, we compute the distance D using the WGD-scaled F_A and F_B, and find the value n^*_4 where the decrease in D first becomes small. Second, we jointly infer the number of clones and the occurrence of a WGD based on a principle of parsimony. If n^*_4 is smaller, we select this solution and infer a WGD. Otherwise, we select the solution with n^*_2 clones and no WGD.

Our model-selection criterion differs from existing methods in two crucial points. First, our criterion explicitly examines the trade-off in number of clones (introducing subclonal CNAs) and the presence/absence of WGD while fitting the data to underlying parameters A, B, and U. In contrast, existing methods do not consider presence/absence

of WGD during model selection and instead select a solution based on values of tumor purity and tumor ploidy, both of which are averages over the distinct tumor clones. Second, our criterion is based on an objective function that is monotonic in the parameter n and thus is better suited for model selection. This is in contrast to existing methods which attempt to fit the data using composite parameters of tumor purity and tumor ploidy, where model selection is complex due to non-monotonicity of distance/likelihood functions. In the remaining of this section, we describe the details of the standard elbow criterion that we use to infer both n_2^* and n_4^*.

We aim to infer the number n of clones when assuming that a WGD occurred or not and, therefore, when the allele-specific fractional copy numbers F^A, F^B are known, as described in Methods. We consider an increasing value of n starting from the presence of a single tumor clone, i.e. $n = 2$, and we denote by $d(n)$ the value of the objective function of D-CACF when n is the given number of clones. Under a principle of parsimony and in the absence of noise, we would choose $n = n^*$ such that n^* is the smallest value with $d(n^*) = 0$. However, the inferred allele-specific fractional copy numbers F^A, F^B are noisy or affected by measurement errors and the objective function is generally greater than 0, i.e. $d(n^*) > 0$. As such, small improvements of the objective function $d(n^*)$ obtained by increasing the value of n^* may not reflect the actual presence of additional tumor clones but they only allow to better explain the noise; this phenomenon is typically known as overfitting. For this reason we use the standard elbow criterion[41], which is typically used in several applications[42–51] and it has been previously applied to a copy-number related problem[23,24].

The key idea of the elbow criterion is to choose a point n^* of the function $d(n)$ such that $d(n^*)$ is a substantial improvement of $d(n^* - 1)$ (i.e. left improvement), while $d(n^* + 1)$ is not a substantial and further improvement of $d(n^*)$ (i.e. right improvement)[41–51]. We introduce a function f to encode this criterion for every value of n such that

$$f(n) = \frac{d(n-1) - d(n)}{d(n-1)} - \frac{d(n) - d(n+1)}{d(n)} \tag{63}$$

where we represent the left improvement by $\frac{d(n-1)-d(n)}{d(n-1)}$ and the right improvement by $\frac{d(n)-d(n+1)}{d(n)}$. The function f measures the tradeoff between the left and right improvements of the objective function $d(n)$ for every n and is closely related to the 2nd-order central finite difference, that is typically used to estimate the value of the second derivative for discrete functions. As such, we choose n^* such that

$$n^* = \underset{2 \leq n \leq \mathcal{U}}{\operatorname{argmax}} f(n) \tag{64}$$

for a given upper bound $\mathcal{U}$ on the number n of clones.

The value of $d(1)$ is needed to compute the left improvement $d(2)$ in the formula above, but is not defined. Thus, we introduce a parameter ε that we use to estimate $d(1)$ as $d(1) = \frac{d(2)}{1-\varepsilon}$. We selected a default value $\varepsilon = 0.35$, based on independent simulations (independent from those in Results) and based on previous results on a related factorization problem[23,24]. This parameter is proportional to the confidence in the presence of a single tumor clone across all the samples, and is user adjustable.

The value of the objective function $d(n)$ and thus the sensitivity of the proposed criterion depends on the size of the clusters. For example, a lower error in the factorization for a small cluster may only produce a small improvement of the function d and lead to choose a lower n. As such, we introduce a second optional parameter ξ that allow to

correspondingly adjust the sensitivity. More specifically, ξ corresponds to an upper bound to the left improvement of the function $d(n)$ and we correspondingly define the function $f(n)$ for every value of n as

$$f(n) = \min\left(\xi, \frac{d(n-1) - d(n)}{d(n-1)}\right) - \frac{d(n) - d(n+1)}{d(n)} \tag{65}$$

As such, the sensitivity of the criterion increases by considering smaller values of ξ as smaller improvements of the objective function d will be sufficient to consider a higher number of clones. In general, this means that variations in the fractional copy numbers of small clusters are more-likely considered to be a signal for a higher number of clones n^* instead of noise when smaller values of ξ are given. HATCHet does not consider the value of ξ by default, but the user can decide to do it when interested in evaluating the presence of more tumor clones characterized by small CNAs.

6 MASCoTE: simulating sequencing reads from multiple mixed samples

In this section, we describe MASCoTE (Multiple Allele-specific Simulation of Copy-number Tumor Evolution), a novel framework to simulate DNA sequencing reads from multiple bulk tumor samples containing both shared and unique clones that acquired different CNAs and WGDs during a common evolutionary scenario. Specifically, we consider each sample as a mixture of a normal clone and a subset of the $n-1$ tumor clones. To simulate the sequencing reads from a mixed sample, most of existing methods mix the sequencing reads that have been separately obtained from the genome of every clone according to the clone proportions[10,13,19,52–55]. These approaches do not account for the different genome lengths of distinct clones; in fact, large CNAs, e.g. CNAs involving whole chromosomes or chromosomal's arms and WGDs, are frequent in cancer[7,34–36,55] and substantially affect the genome length of the corresponding clone. However, the proportions of sequencing reads in a mixed sample depends on the genome lengths of all the clones in the sample[56] and discarding this dependency leads to incorrect proportions of sequencing reads (Supplementary Figs. 4 and 5).

We introduce MASCoTE, an algorithm to simulate the DNA sequencing reads obtained from multiple bulk tumor samples of the same patient by accounting for the different genome lengths of distinct clones (Supplementary Fig. 6). The main feature of MASCoTE is the algorithm described in Methods to identify the proportions of sequencing reads in the samples according to the genome lengths of the distinct clones present in these samples. Additionally, MASCoTE comprises other key features:

- MASCoTE specifically simulates the two haplotypes of each chromosome in the normal diploid genome, i.e. the homologous copies of each chromosome. Each haplotype is thus characterized by specific heterozygous and homozygous germline SNPs.
- MASCoTE randomly simulates a phylogenetic tree which describes the evolution of the tumor clones. Each node of the the phylogenetic tree corresponds to a clone and the tree is always rooted in the normal clone. The root has a single edge, called trunk, going into the internal node representing the founder tumor clone which accumulates the somatic mutations inherited by all tumor clones. As such, the extant tumor clones present in the samples correspond to the leaves of the tree, while the ancestor clones correspond to the internal nodes and are not present in the samples.

- MASCoTE simulates haplotype-specific CNAs for each phylogenetic branch (i.e. edge of the tree) and these mutations are applied in arbitrary order to the the genome of the parent clone in order to obtain the genome of the child clone. MASCoTE simulates CNAs as duplications and deletions, and the number of simulated CNAs for each branch is obtained from a normal distribution whose mean and variance are given (the values for the trunk and the other edges may differ). Moreover, MASCoTE allows the generation of different kinds of CNAs, ranging from focal CNAs to CNAs involving chromosomal's arms or whole chromosomes. As such, the genome of each tumor clone is affected by an ordered sequence of CNAs obtained by traversing the path from the root to the node and applying the CNAs in corresponding order.
- MASCoTE can simulate WGDs in the branches of the phylogenetic tree. Each WGD duplicates the entire genome content and, therefore, each haplotype of every chromosome. When a WGD is simulated in a certain branch, MASCoTE applies the WGD in a random order with respect to the other CNAs in the same branch. As such, MASCoTE can simulate CNAs that occur both before and after WGDs, especially when a WGD occurs in the trunk of the phylogenetic tree and affects the genome of all tumor clones.

In the following we describe the four steps of MASCoTE, which have been applied to generate the bulk tumor samples in Results.

6.1 Simulating diploid human genome

MASCoTE simulates a diploid human genome by introducing heterozygous and homozygous germline SNPs to a reference genome (e.g. hg19) similarly to current simulating methods[57,58] (Supplementary Fig. 6a). In particular, we provided MASCoTE with lists of germline SNPs obtained from existing databases (e.g. dbSNP or 1000 Genome Project). As such, the haplotype of each chromosome is characterized by specific SNPs and the two haplotypes are considered independently.

6.2 Simulating tumor evolution

MASCoTE simulates a random phylogenetic tree and introduces a number of CNAs to each branch (distinguishing between the trunk and the other edges of the tree) and a WGD in the trunk, when required (Supplementary Fig. 6b). We thus consider distributions of the quantity and size of CNAs that are in accordance with those reported in previous pan-cancer analysis[34]. More specifically, we simulated CNAs of different size according to the frequencies described in previous pan-cancer analysis including: focal CNAs with <1Mb, small between 3-5Mb, CNAs between 10-20Mb, and chromosomal-arm aberrations (involving either a haplotype's arm or the whole haplotype). The genomic location of the CNAs is randomly chosen according to the chromosome's length (which varies under the effects of the CNAs already applied). We simulate haplotype-specific CNAs corresponding to tandem duplications and deletions by randomly choosing the affected haplotype. A WGD is also simulated in the trunk by duplicating the whole genome content. The CNAs as well as the WGD assigned to each edge are sorted randomly.

6.3 Simulating DNA sequencing reads

MASCoTE uses ART[59] to simulate sequencing reads from the genome of each simulated clone (Supplementary Fig. 6c). More specifically, we considered a fold sequencing coverage ranging between 40-60$\times$ and we used the default parameters for paired-end read simulation. MASCoTE also uses BWA[60] to map the sequencing reads to a human reference genome; in this case, we considered the same reference genome used for simulating the genomes of the clones (i.e. hg19). As we consider the haplotypes separately for each chromosome and we jointly sequence them, we guarantee that the reads are uniformly sequenced from the two haplotypes according to their length and their copy numbers. This is important because HATCHet as well as the other considered methods for allele-specific CNA inference rely on the observed BAF, which estimates the proportion of reads belonging to the two alleles of each genomic region that are part of the two distinct haplotypes.

6.4 Simulating DNA sequencing reads from multiple bulk tumor samples

MASCoTE simulates k bulk tumor samples by mixing the reads from the simulated clones according to the proportions obtained through the method described in Methods for given clone proportions (Supplementary Fig. 6d). Observe that the genome lengths of all the clones are known and we are thus able to apply the method described in Methods because the CNAs have been simulated and stored together with the corresponding allele-specific copy numbers by MASCoTE. For each patient, we simulated every sample by randomly choosing a subset of the clones and by selecting random clone proportions. We constrained each subset to contain the normal clone and at least one tumor clone. Multiple samples for the same patient were hence simulated by considering different subsets from the same simulated collection of clones and guaranteeing every clone is present in at least one sample. Furthermore, MASCoTE samples and mixes the sequencing reads from each clone according to the corrected proportions using SAMtools[25] and each sample has been processed according to GATK best practices[61].

Supplementary Notes

1 Experimental setup for simulated data

We simulated bulk tumor samples using MASCoTE to assess the performance of HATCHet. Specifically, we simulated 256 bulk tumor samples for 64 patients, 128 without WGDs and 128 with a WGD (Supplementary Fig. 7). Each tumor comprises a normal clone and 2-3 tumor clones, and 3-5 samples are obtained from each patient such that each sample contains a subset of the corresponding clones. We compared HATCHet with six current state-of-the-art methods for copy-number deconvolution, i.e. Battenberg[9], TITAN[13], THetA[17,18], cloneHD[19], Canopy[11] (with fractional copy numbers from FALCON[10]), and ReMixT[21], on these simulated samples.

We executed every method with default parameters and according to available instructions. Here, we provide details for the execution of each method.

Battenberg We executed Battenberg on each sample by using the full pipelines that are available at `https://github.com/cancerit/cgpBattenberg` and `https://github.com/Wedge-Oxford/battenberg` using default parameters. More specifically, we used the whole-genome sequencing (WGS) pipeline implementation of R Battenberg v2.2.7. As only autosomes are present in the simulated data, we removed the sex chromosomes X and Y from the Battenberg configuration files. Battenberg directly considers in input the sequencing reads in a BAM file and uses specific tools for some computations, including: ASCAT[8], that is used to infer possible values of tumor ploidy and purity, and IMPUTE2[62], that is used to phase germline heterozygous SNPs in order to better estimate the values of BAF. In addition to ASCAT, Battenberg uses other methods to further infer possible values of the tumor ploidy and tumor purity. When running Battenberg on the simulated samples with no WGD by fixing the true value of the main parameters, we ran Battenberg by selecting the inferred values of tumor ploidy and tumor purity that are the closest to the true values for the corresponding sample or by providing the true values of tumor ploidy and purity. In fact, Battenberg finds among all possibilities at least one pair of values of tumor ploidy and purity that are approximately equal to the true values for most of the simulated datasets with no WGD in this work. We provided the true values of tumor ploidy and tumor purity when this was not the case. When running Battenberg on all simulated samples with or without WGD considering free values of the parameters, we ran Battenberg with all the default values.

TITAN We executed TITAN on each sample by using the pipelines and scripts that are available at `https://github.com/gavinha/TitanCNA` using default parameters. More specifically, we used the standard whole-genome sequencing analysis pipeline for TITAN v1.13.1 that directly considers a BAM file in input. The only additional tool needed by TITAN is HMMcopy[63] that is used to compute the RDR and to correct it by biases, including GC and mappability biases, and to obtain a rough estimation of the copy numbers. TITAN has three main parameters: an integer initialization for tumor ploidy, a number of clonal clusters, and the maximum copy number $c_{\max}$. More specifically, TITAN assumes there is only a single copy-number state different from $(1, 1)$ for every segment and, thus, clusters all segments with the same cellular prevalence (i.e. the sum of the proportions of clones with this non-diploid state).

For example, there are generally 3 clusters for 2 tumor clones in a sample: a cluster is representing CNAs only in the first clone, another cluster is representing CNAs only in the second clone, and the last cluster for CNAs present in both the clones. When running TITAN on the simulated samples with no WGD by fixing the true value of the main parameters, we provided the initialization of tumor ploidy that is the closest integer to the true value, the true number of clonal clusters, and the true value of $c_{\max}$. When running TITAN on all simulated samples with or without WGD by considering free values of the parameters, we ran TITAN with the default initialization values of the tumor ploidy in $\{2, 3, 4\}$, the default maximum number of clonal cluster equal to 5, and $c_{\max} = 8$ that is the same value used by all the other methods as it is a reasonable value both in the presence or absence of a WGD and it is in accordance with our simulated data. We used the script included in the standard whole-genome sequencing analysis pipeline to automatically choose the best solution among the ones found using different initializing values of tumor ploidy and different number of clonal clusters. In particular, the best ploidy initialization is chosen as the one with consistently larger log-likelihood when considering the solutions with the same number of clonal clusters and the best number of clonal clusters is determined using the minimum "S_Dbw validity index" (that uses both log ratio and allele ratio).

THetA We executed THetA on each sample by using the scripts that are available at `https://github.com/raphael-group/THetA` using default parameters for THetA 2. THetA does not directly consider in input a BAM file for the tumor sample but it considers the read counts for segments of the genome and the allele counts for germline SNPs. We hence computed these data using the same process applied by HATCHet; more specifically, we obtained the segments by merging neighboring bins that are part of the same cluster computed by HATCHet. THetA has two main parameters: the number of tumor clones and the maximum copy number $c_{\max}$. When running THetA on the simulated samples with no WGD by fixing the true value of the main parameters, we provided the true number of tumor clones present in the sample and the true value of $c_{\max}$. THetA has not been run on all simulated samples with or without WGD considering free values of the parameters because THetA has not been designed to deal with WGDs.

cloneHD We executed cloneHD jointly on all the samples from the same patient by using the full pipeline that is available and described at `https://github.com/andrej-fischer/cloneHD`, by using default values of all parameters, and by excluding the SNV analysis. cloneHD does not directly consider in input a BAM file and we thus computed the required input by using the same values of read counts obtained by HATCHet. cloneHD also includes a pre-processing step and a method called filterHD[19] to infer the segments by jointly considering RDR and BAF. cloneHD has two main parameters: the number of tumor clones and the maximum copy number $c_{\max}$. When running cloneHD on the simulated samples with no WGD by fixing the true value of the main parameters, we provided the true number of clones and the true value of $c_{\max}$. When running cloneHD on all simulated samples with or without WGD considering free values of the parameters, we ran cloneHD by considering the default values for all the parameters and by providing a maximum number of tumor clones equal to 3 as in our simulations, and $c_{\max} = 8$ that is the same value used by all the other methods as it is a reasonable value both in the presence or absence of a WGD and it is in accordance with our simulated data. In addition, as suggested in previous benchmarks[64] we ran cloneHD by considering different values in $\{0.95, 0.99, 1.0\}$ for the penalty concerning the prediction of a WGD. We thus chose to use a value equal to 0.95 as

done in previous benchmarks[64] even if the different values of this penalty resulted in little or no differences for all the samples in terms of predictions of WGDs.

Canopy with FALCON We executed Canopy with FALCON jointly on all the samples from the same patient by using the MARATHON pipeline[65] that is available and described at `https://github.com/yuchaojiang/MARATHON` (commit 553595a) and the scripts available at `https://github.com/yuchaojiang/Canopy` (commit 150fbac), by considering the detailed demo available at `https://rawgit.com/yuchaojiang/MARATHON/master/notebook/MARATHON.html#tumor-allele-specific-copy-number-by-wgs`, by using default values of all parameters, and by excluding the SNV analysis. FALCON analyzes each sample independently and considers in input the read counts for heterozygous germline SNPs that we identified and obtained with the same approach used by HATCHet. We followed the previously described manual procedure[11] to obtain the input segments for Canopy from the results of FALCON. When running Canopy with FALCON on the simulated samples with no WGD by fixing the true value of the main parameters, we provided the true number of clones (the only available true parameter). When running Canopy with FALCON on all simulated samples with or without WGD considering free values of the parameters, we ran Canopy with FALCON by considering the default values for all the parameters and settings and by providing a maximum number of clones of 5. The pipeline automatically selects the best solution based on the Bayesian information criterion (BIC).

ReMixT We executed ReMixT jointly on all the samples from the same patient by using the full pipeline which is available at `https://github.com/amcpherson/remixt` (commit bd18fe0) by using the default values of all parameters and by providing the true known breakpoints. ReMixT considers each sample independently and directly starts from the BAM file of each bulk tumor sample and the BAM file of the corresponding matched-normal sample. When running ReMixT on the simulated samples with no WGD by fixing the true value of the main parameters, we ran ReMixT by providing the true value of the maximum copy number $c_{\max}$ and the true value of the tumor ploidy within a relatively small interval between the lower and upper bounds. When running ReMixT on all simulated samples with or without WGD considering free values of the parameters, we ran ReMixT by considering the default values for all the parameters, and $c_{\max} = 8$ that is the same value used by all the other methods as it is a reasonable value both in the presence or absence of a WGD and it is in accordance with our simulated data. Additionally, we increased the maximum value of tumor ploidy to 5 according to the true values of tumor ploidy for the simulated samples with a WGD. Note that we provided the true known breakpoints even when running ReMixT on all simulated samples with or without WGD considering free values of the parameters.

HATCHet We executed HATCHet both individually on each sample and jointly on all the samples from the same patient using default parameters. The full pipeline is available at `https://github.com/raphael-group/hatchet/tree/master/script` and directly starts from the BAM file of every bulk tumor sample and the BAM file of a matched-normal sample. HATCHet has three main parameters: the number of clones, the maximum copy number $c_{\max}$, and the minimum clone proportion $u_{\min}$. When running HATCHet on the simulated samples with no WGD by fixing the true value of the main parameters, we provided the true number of clones and the true value of $c_{\max}$. When running

HATCHet on all simulated samples with or without WGD considering free values of the parameters, we ran HATCHet by considering the default values for all the parameters, by providing a maximum number of tumor clones equal to 8, and $c_{\text{max}} = 8$ that is the same value used by all the other methods as it is a reasonable value both in the presence or absence of a WGD and it is in accordance with our simulated data. In all the cases, a default value of u_{min} equal to 0.03 was considered.

2 Metrics for evaluating performance on simulated data

To investigate different aspects of the solutions inferred by HATCHet and by the other methods on simulated data, we considered different metrics. Each metric corresponds to a measure of the distance for a sample between the solution inferred by one of the methods and the true solution that is available from the simulating process. For simplicity, we describe the metrics for a single sample as we apply those on each sample separately. The definitions of the metrics are based on some values that we can easily compute from the solutions inferred by every method. We first define these basic values and we next describe each metric separately.

Basic values All the basic values that we compute from the solution of a method and from the corresponding true solution are based on a collection of $\mathfrak{m}$ segments. To obtain these $\mathfrak{m}$ segments, we partition the L genomic positions of the reference genome such that each segment $\mathfrak{s}$ contains $\mathfrak{l}_{\mathfrak{s}}$ neighboring genomic positions with the same copy-number state in every clone present in the inferred and true solutions. As such, we describe three basic values.

First, we compute the set $\mathcal{S}_{\mathfrak{s}}$ containing the copy-number states inferred for a segment $\mathfrak{s}$ in different clones. For example, when considering the solutions of HATCHet we obtain

$$\mathcal{S}_{\mathfrak{s}} = \{(a_{i,s}, b_{s,i}) : 1 \leq i \leq n\} \tag{66}$$

assuming that segment $\mathfrak{s}$ is part of the cluster s. We equivalently compute the set $\overline{\mathcal{S}}_{\mathfrak{s}}$ containing the true copy-number states in the different clones of the true solution for each segment $\mathfrak{s}$.

Second, we compute the set $\mathcal{C}_{\mathfrak{s}}$ containing the inferred total copy numbers for a segment $\mathfrak{s}$ in different clones. When considering the solutions of HATCHet we obtain

$$\mathcal{C}_{\mathfrak{s}} = \{c_{s,i} : 1 \leq i \leq n, c_{s,i} = a_{s,i} + b_{s,i}\} \tag{67}$$

assuming that segment $\mathfrak{s}$ is part of the cluster s. We equivalently compute the set $\overline{\mathcal{C}}_{\mathfrak{s}}$ containing the true total copy numbers in the different clones of the true solution for each segment $\mathfrak{s}$.

Last, we compute the mixing proportion $\mathfrak{u}_{\mathfrak{s},\mathfrak{c}}$ for every segment $\mathfrak{s}$ and every possible value $\mathfrak{c} \in \mathbb{N}$ of the total copy numbers as the sum of the proportions of all clones with a total copy number equal to $\mathfrak{c}$ in $\mathfrak{s}$. For example, when considering the solutions of HATCHet we obtain

$$\mathfrak{u}_{\mathfrak{s},\mathfrak{c}} = \sum_{1 \leq i \leq n : c_{s,i} = \mathfrak{c}} u_{i,p} \tag{68}$$

assuming that segment $\mathfrak{s}$ is part of the cluster s and that we are considering a sample p. We equivalently compute the mixing proportion $\overline{\mathfrak{u}}_{\mathfrak{s},\mathfrak{c}}$ for every segment $\mathfrak{s}$ and every possible value $\mathfrak{c} \in \mathbb{N}$ of total copy numbers as the sum of the proportions of all clones in the true solution with a total copy number equal to $\mathfrak{c}$ in $\mathfrak{s}$.

Observe that we can easily compute every $\mathcal{S}_{\mathfrak{s}}, \mathcal{C}_{\mathfrak{s}}, \mathfrak{u}_{\mathfrak{s},\mathfrak{c}}$ from the solution inferred by each considered method as well as we can easily compute every $\overline{\mathcal{S}}_{\mathfrak{s}}, \overline{\mathcal{C}}_{\mathfrak{s}}, \overline{\mathfrak{u}}_{\mathfrak{s},\mathfrak{c}}$ from true solutions. Hence, we now present all the metrics based on these values that we use to compare the performance of all methods.

Precision and recall of total copy numbers We assess the inferred total copy numbers in terms of precision and recall that we compute according to the standard definitions used in the context of information retrieval. As such, we compare for each segment the retrieved copy-number states, which are the inferred ones in $\mathcal{C}_{\mathfrak{s}}$, with the relevant copy-number states, which are the true ones in $\overline{\mathcal{C}}_{\mathfrak{s}}$. The precision of each segment corresponds to the fraction of copy-number states that are relevant and retrieved over all the retrieved copy-number states. The recall of each segment corresponds to the fraction of copy-number states that are relevant and retrieved over all the relevant copy-number states. We consider the average values of precision and recall per genome position by considering the length $\mathfrak{l}_{\mathfrak{s}}$ of each segment $\mathfrak{s}$. Thus, we compute the precision as

$$\frac{1}{L} \sum_{1 \leq \mathfrak{s} \leq \mathfrak{m}} \mathfrak{l}_{\mathfrak{s}} \frac{|\overline{\mathcal{C}}_{\mathfrak{s}} \cap \mathcal{C}_{\mathfrak{s}}|}{|\mathcal{C}_{\mathfrak{s}}|} \tag{69}$$

and the recall as

$$\frac{1}{L} \sum_{1 \leq \mathfrak{s} \leq \mathfrak{m}} \mathfrak{l}_{\mathfrak{s}} \frac{|\overline{\mathcal{C}}_{\mathfrak{s}} \cap \mathcal{C}_{\mathfrak{s}}|}{|\overline{\mathcal{C}}_{\mathfrak{s}}|}. \tag{70}$$

In addition, we combine precision and recall to compute the accuracy as

$$\frac{1}{L} \sum_{1 \leq \mathfrak{s} \leq \mathfrak{m}} \mathfrak{l}_{\mathfrak{s}} \frac{|\overline{\mathcal{C}}_{\mathfrak{s}} \cap \mathcal{C}_{\mathfrak{s}}|}{|\overline{\mathcal{C}}_{\mathfrak{s}} \cup \mathcal{C}_{\mathfrak{s}}|}. \tag{71}$$

Precision and recall of copy-number states We assess the inferred copy-number states in terms of precision and recall that we define similarly as above by considering copy-number states in $\mathcal{S}_{\mathfrak{s}}, \overline{\mathcal{S}}_{\mathfrak{s}}$ instead of total copy numbers $\mathcal{C}_{\mathfrak{s}}, \overline{\mathcal{C}}_{\mathfrak{s}}$. As such, the precision of each segment corresponds to the fraction of copy-number states that are relevant and retrieved over all the retrieved copy-number states and the recall corresponds to the fraction of copy-number states that are relevant and retrieved over all the relevant copy-number states. We consider the average values of precision and recall per genome position by considering the length $\mathfrak{l}_{\mathfrak{s}}$ of the each segment $\mathfrak{s}$. Thus, we compute the precision as

$$\frac{1}{L} \sum_{1 \leq \mathfrak{s} \leq \mathfrak{m}} \mathfrak{l}_{\mathfrak{s}} \frac{|\overline{\mathcal{S}}_{\mathfrak{s}} \cap \mathcal{S}_{\mathfrak{s}}|}{|\mathcal{S}_{\mathfrak{s}}|} \tag{72}$$

and the recall as

$$\frac{1}{L} \sum_{1 \leq \mathfrak{s} \leq \mathfrak{m}} \mathfrak{l}_{\mathfrak{s}} \frac{|\overline{\mathcal{S}}_{\mathfrak{s}} \cap \mathcal{S}_{\mathfrak{s}}|}{|\overline{\mathcal{S}}_{\mathfrak{s}}|}. \tag{73}$$

In addition, we combine precision and recall to compute the accuracy as

$$\frac{1}{L} \sum_{1 \leq \mathfrak{s} \leq \mathfrak{m}} \mathfrak{l}_{\mathfrak{s}} \frac{|\overline{\mathcal{S}}_{\mathfrak{s}} \cap \mathcal{S}_{\mathfrak{s}}|}{|\overline{\mathcal{S}}_{\mathfrak{s}} \cup \mathcal{S}_{\mathfrak{s}}|}. \tag{74}$$

Precision and recall of total copy numbers per segment To assess the capability to infer the copy numbers of short genomic regions, we also compute the precision and recall for total copy numbers per segment. As such, we consider the unweighted versions of the previous definitions of precision and recall for total copy numbers. Moreover, we only consider segments that have a size of at least $50kb$, i.e. $\{\mathfrak{s} : \mathfrak{l}_{\mathfrak{s}} > 50k\}$, that is the minimum value higher than the resolution of every method. Thus, we compute the precision as

$$\frac{1}{\mathfrak{m}} \sum_{\substack{1 \leq \mathfrak{s} \leq \mathfrak{m}: \\ \mathfrak{l}_s > 50k}} \frac{|\overline{\mathcal{C}}_{\mathfrak{s}} \cap \mathcal{C}_{\mathfrak{s}}|}{|\mathcal{C}_{\mathfrak{s}}|} \tag{75}$$

and the recall as

$$\frac{1}{\mathfrak{m}} \sum_{\substack{1 \leq \mathfrak{s} \leq \mathfrak{m}: \\ \mathfrak{l}_s > 50k}} \frac{|\overline{\mathcal{C}}_{\mathfrak{s}} \cap \mathcal{C}_{\mathfrak{s}}|}{|\overline{\mathcal{C}}_{\mathfrak{s}}|}. \tag{76}$$

In addition, we combine precision and recall to compute the accuracy as

$$\frac{1}{\mathfrak{m}} \sum_{\substack{1 \leq \mathfrak{s} \leq \mathfrak{m}: \\ \mathfrak{l}_s > 50k}} \frac{|\overline{\mathcal{C}}_{\mathfrak{s}} \cap \mathcal{C}_{\mathfrak{s}}|}{|\overline{\mathcal{C}}_{\mathfrak{s}} \cup \mathcal{C}_{\mathfrak{s}}|}. \tag{77}$$

Average error per genome position We jointly assess the inferred copy numbers and the corresponding clone proportions as the error per genome position that we define now. The mixing proportions $\mathfrak{u}_{\mathfrak{s},\mathfrak{c}}$ define a probability distribution among all possible values of total copy numbers in a segment $\mathfrak{s}$ as $\mathfrak{u}_{\mathfrak{s},\mathfrak{c}} \in [0, 1]$ for very $\mathfrak{c} \in \mathbb{N}$ and $\sum_{\mathfrak{c} \in \mathbb{N}} \mathfrak{u}_{\mathfrak{s},\mathfrak{c}} = 1$. As such, we compute the error for each segment $\mathfrak{s}$ as the total variation distance between the probability distributions defined by the inferred and true mixing proportions, i.e. $\mathfrak{u}_{\mathfrak{s},\mathfrak{c}}$ and $\overline{\mathfrak{u}}_{\mathfrak{s},\mathfrak{c}}$, respectively, across all values $\mathfrak{c} \in \mathbb{N}$ of total copy numbers. The total variation distance is in fact a standard measure to compare two probability distributions. We thus compute the average error per genome position by considering the length of each segment and we have the following

$$\frac{1}{L} \sum_{1 \leq \mathfrak{s} \leq \mathfrak{m}} \mathfrak{l}_{\mathfrak{s}} \sup_{\mathfrak{c} \in \mathbb{N}} |\mathfrak{u}_{\mathfrak{s},\mathfrak{c}} - \overline{\mathfrak{u}}_{\mathfrak{s},\mathfrak{c}}|. \tag{78}$$

The inferred mixing proportion $\mathfrak{u}_{\mathfrak{s},\mathfrak{c}}$ as well as the true mixing proportion $\overline{\mathfrak{u}}_{\mathfrak{s},\mathfrak{c}}$ have non-zero values only for a finite number of values for $\mathfrak{c}$ included in $\mathcal{C}_{\mathfrak{s}} \cup \overline{\mathcal{C}}_{\mathfrak{s}}$. Therefore, we compute the average error per genome position as

$$\frac{1}{L} \sum_{1 \leq \mathfrak{s} \leq \mathfrak{m}} \mathfrak{l}_{\mathfrak{s}} \max_{\mathfrak{c} \in \mathcal{C}_{\mathfrak{s}} \cup \overline{\mathcal{C}}_{\mathfrak{s}}} |\mathfrak{u}_{\mathfrak{s},\mathfrak{c}} - \overline{\mathfrak{u}}_{\mathfrak{s},\mathfrak{c}}|. \tag{79}$$

Observe that this metric jointly accounts for the error made when a total copy number is retrieved correctly but with wrong clone proportions, as well as the error made when a total copy number is retrieved incorrectly (not present in the true solution).

Average amplification-deletion error per genome position One may be only interested in knowing whether a segment is amplified or deleted with respect to the other segments; in this case, the exact copy numbers of a segment or the presence of a WGD are not relevant. We introduce the amplification-deletion error per genome position to assess the capability of retrieving this information. To distinguish whether a total copy number is either amplified or deleted

or none, we consider the base total copy number Θ such that $\Theta = 2$ when there is no WGD and $\Theta = 4$ when there is a WGD. More specifically, we define a function $z : \mathbb{N} \to \{-1, 0, 1\}$ to reduce every inferred total copy number $\mathfrak{c} \in \mathcal{C}_{\mathfrak{s}}$ of each segment $\mathfrak{s}$ to the set $\{-1, 0, 1\}$ based on Θ as follows

$$z(\mathfrak{c}) = \begin{cases} -1 & \mathfrak{c} < \Theta \\ 0 & \mathfrak{c} = \Theta \\ 1 & \mathfrak{c} > \Theta \end{cases}. \tag{80}$$

As such, we define the set $\mathcal{Z}_{\mathfrak{s}}$ for every segment $\mathfrak{s}$ as

$$\mathcal{Z}_{\mathfrak{s}} = \{\mathfrak{z} : \mathfrak{c} \in \mathcal{C}_{\mathfrak{s}}, \mathfrak{z} = z(\mathfrak{c})\} \tag{81}$$

and we equivalently compute the set $\overline{\mathcal{Z}}_{\mathfrak{s}}$ by considering the true total copy numbers in $\overline{\mathcal{C}}_{\mathfrak{s}}$ as

$$\overline{\mathcal{Z}}_{\mathfrak{s}} = \{\mathfrak{z} : \mathfrak{c} \in \overline{\mathcal{C}}_{\mathfrak{s}}, \mathfrak{z} = z(\mathfrak{c})\}. \tag{82}$$

Given these reductions, we additionally compute for every segment $\mathfrak{s}$ the reduced inferred mixing proportion $\mathfrak{v}_{\mathfrak{s},\mathfrak{z}}$ and the reduced true mixing proportion $\overline{\mathfrak{v}}_{\mathfrak{s},\mathfrak{z}}$ for every value $\mathfrak{z} \in \mathcal{Z}_{\mathfrak{s}}$ and $\mathfrak{z} \in \overline{\mathcal{Z}}_{\mathfrak{s}}$, respectively such that

$$\mathfrak{v}_{\mathfrak{s},\mathfrak{z}} = \sum_{\mathfrak{c} \in \mathcal{C}, \mathfrak{z} = z(\mathfrak{c})} \mathfrak{u}_{\mathfrak{s},\mathfrak{c}} \tag{83}$$

and

$$\overline{\mathfrak{v}}_{\mathfrak{s},\mathfrak{z}} = \sum_{\mathfrak{c} \in \mathcal{C}, \mathfrak{z} = z(\mathfrak{c})} \overline{\mathfrak{u}}_{\mathfrak{s},\mathfrak{c}}. \tag{84}$$

Using these reductions, we compute the average amplification-deletion error per genome position similarly to the average error per genome position described above as

$$\frac{1}{L} \sum_{1 \leq \mathfrak{s} \leq \mathfrak{m}} \mathfrak{l}_{\mathfrak{s}} \max_{\mathfrak{z} \in \mathcal{Z}_{\mathfrak{s}} \cup \overline{\mathcal{Z}}_{\mathfrak{s}}} |\mathfrak{v}_{\mathfrak{s},\mathfrak{z}} - \overline{\mathfrak{v}}_{\mathfrak{s},\mathfrak{z}}|. \tag{85}$$

Relative error of tumor purity We compare the inferred tumor purity μ_p with the true tumor purity $\overline{\mu}_p$ of a sample p as the relative error that we compute as

$$\frac{|\overline{\mu}_p - \mu_p|}{\overline{\mu}_p}. \tag{86}$$

The values of μ_p and $\overline{\mu}_p$ are directly provided by the solution of each method and the true solution of sample p, respectively.

Relative error of tumor ploidy We compare the inferred tumor ploidy ρ_p with the true tumor ploidy $\overline{\rho}_p$ as the relative error that we compute as

$$\frac{|\overline{\rho}_p - \rho_p|}{\overline{\rho}_p}. \tag{87}$$

We compute ρ_p and $\overline{\rho}_p$ from the solution of each method and the true solution of sample p, respectively. More specifically, we compute ρ_p and $\overline{\rho}_p$ following the model in Supplementary Method 1 and we thus obtain the inferred

tumor ploidy as

$$\rho_p = \frac{1}{L} \sum_{1 \leq \mathfrak{s} \leq \mathfrak{m}} \sum_{\mathfrak{c} \in \mathfrak{C}_\mathfrak{s}} \mathfrak{c} \hat{\mathfrak{u}}_{\mathfrak{s},\mathfrak{c}} \tag{88}$$

where $\hat{\mathfrak{u}}_{\mathfrak{s},\mathfrak{c}}$ is the inferred mixing proportion $\mathfrak{u}_{\mathfrak{s},\mathfrak{c}}$ scaled by the inferred tumor purity μ_p, i.e. $\hat{\mathfrak{u}}_{\mathfrak{s},\mathfrak{c}} = \frac{\mathfrak{u}_{\mathfrak{s},\mathfrak{c}}}{\mu_p}$, and we obtain the true tumor ploidy as

$$\overline{\rho}_p = \frac{1}{L} \sum_{1 \leq \mathfrak{s} \leq \mathfrak{m}} \sum_{\mathfrak{c} \in \overline{\mathfrak{C}}_\mathfrak{s}} \mathfrak{c} \tilde{\mathfrak{u}}_{\mathfrak{s},\mathfrak{c}} \tag{89}$$

where $\tilde{\mathfrak{u}}_{\mathfrak{s},\mathfrak{c}}$ is the true mixing proportion $\overline{\mathfrak{u}}_{\mathfrak{s},\mathfrak{c}}$ scaled by the true tumor purity $\overline{\mu}_p$, i.e. $\tilde{\mathfrak{u}}_{\mathfrak{s},\mathfrak{c}} = \frac{\overline{\mathfrak{u}}_{\mathfrak{s},\mathfrak{c}}}{\overline{\mu}_p}$.

Precision and recall for the absence and presence of a WGD We assess the predictions of the presence and absence of WGDs in terms of precision and recall. While HATCHet explicitly predicts the presence of a WGD, existing methods base the prediction on the inferred value of the tumor ploidy ρ_p in a sample p. According both to these methods and to the values in our simulations, we say that a method predicts for a sample p the absence of a WGD if $\rho_p \in [0, 3)$ or predicts the presence of a WGD if $\rho_p \in [3, 4.8]$. Moreover, higher values of the ploidy, i.e. $\rho_p \in (4.8, +\infty)$, indicate the presence of multiple WGDs in p. We compute precision and recall considering these definitions for existing methods. We do the same also for HATCHet because the explicit predictions of HATCHet provide the same results for all simulated samples when computing the tumor ploidy from the HATCHet's solutions as described in Supplementary Method 1. More specifically, we consider the standard definitions of precision and recall in the context of classification and we compute these considering both the absence and presence as follows:

- Precision for the absence of a WGD corresponds to

$$\frac{|\{p : 1 \leq p \leq \mathfrak{k}, \overline{\rho}_p \in [0,3), \rho_p \in [0,3)\}|}{|\{p : 1 \leq p \leq \mathfrak{k}, \overline{\rho}_p \in [0,3), \rho_p \in [0,3)\}| + |\{p : 1 \leq p \leq \mathfrak{k}, \overline{\rho}_p \in [3,+\infty), \rho_p \in [0,3)\}|}. \tag{90}$$

- Recall for the absence of a WGD corresponds to

$$\frac{|\{p : 1 \leq p \leq \mathfrak{k}, \overline{\rho}_p \in [0,3), \rho_p \in [0,3)\}|}{|\{p : 1 \leq p \leq \mathfrak{k}, \overline{\rho}_p \in [0,3), \rho_p \in [0,3)\}| + |\{p : 1 \leq p \leq \mathfrak{k}, \overline{\rho}_p \in [0,3), \rho_p \in [3,+\infty)\}|}. \tag{91}$$

- Precision for the presence of a WGD corresponds to

$$\frac{|\{p : 1 \leq p \leq \mathfrak{k}, \overline{\rho}_p \in [3,4.8], \rho_p \in [3,4.8]\}|}{|\{p : 1 \leq p \leq \mathfrak{k}, \overline{\rho}_p \in [3,4.8], \rho_p \in [3,4.8]\}| + |\{p : 1 \leq p \leq \mathfrak{k}, \overline{\rho}_p \notin [3,4.8], \rho_p \in [3,4.8]\}|}. \tag{92}$$

- Recall for the presence of a WGD corresponds to

$$\frac{|\{p : 1 \leq p \leq \mathfrak{k}, \overline{\rho}_p \in [3,4.8], \rho_p \in [3,4.8]\}|}{|\{p : 1 \leq p \leq \mathfrak{k}, \overline{\rho}_p \in [3,4.8], \rho_p \in [3,4.8]\}| + |\{p : 1 \leq p \leq \mathfrak{k}, \overline{\rho}_p \in [3,4.8], \rho_p \notin [3,4.8]\}|}. \tag{93}$$

Observe that we compute precision and recall separately for the prediction of the absence or presence of a WGD because multiple WGDs can be predicted and because precision and recall do not consider true negatives.

3 Additional results on simulated data with fixed parameters

We ran all methods on the 128 samples from 32 patients without a WGD and also providing the true value of the main parameters (e.g. tumor ploidy, number of clones, and maximum copy number) required for each method. This provides a baseline comparison of the performance of each method in determining copy numbers and proportions without the difficulty of model selection. Note that for ReMixT we provided the true simulated breakpoints in both parts, and we therefore did not assess breakpoint predictions in this analysis. We found that HATCHet and single-sample HATCHet outperform all other methods (Fig. 2a, Supplementary Fig. 8a, and Supplementary Figs. 9–12). The advantage demonstrated by single-sample HATCHet is likely due to HATCHet's key features other than the joint analysis of multiple samples, which are described in the comparison of different algorithms in Methods. In particular, we observed that Battenberg and TITAN, which infer the copy numbers of genomic regions independently, performed substantially worse than THetA, cloneHD, ReMixT, and HATCHet, which group copy numbers into the clones present in a sample. Note that Canopy exhibited the lowest performance, suggesting that Canopy's integrative model of SNVs and CNAs is not suitable for the deconvolution of CNAs only. Furthermore, we observed that cloneHD – the only existing method that performs copy-number deconvolution from multiple samples simultaneously – shows only a modest gain over THetA and ReMixT which analyze samples individually; however, cloneHD performed worse than single-sample HATCHet. This suggests that cloneHD is not deriving maximum benefit from multiple samples, perhaps because its model assumes that the same few clones are present in all samples.

Our extensive analysis with different metrics (Supplementary Note 2) further reveals interesting differences among the considered methods. TITAN performs better than Battenberg for the majority of the metrics when considering total copy numbers and, especially, when considering the average error per segment and the average amplification-deletion error per genomic position (Supplementary Figs. 9, 10, and 12). However, Battenberg performs better than TITAN when considering allele-specific metrics (Supplementary Fig. 11); this result may be due to a better estimation of BAFs that Battenberg achieves by jointly phasing germline heterozygous SNPs. Moreover, THetA and, especially, TITAN are the methods that constantly show the highest variances in the results compared to the others (Fig. 2a, and Supplementary Figs. 9, 10, 11, and 12). Last, we would expect a general improvement of the performance when considering the amplification-deletion average error compared to the average error per genome position, as the first metric does not require to identify the exact copy numbers. However, all methods show only a limited improvement of their performance in this case: this suggests that all methods infer the correct copy number for most of the genomic positions when they correctly identifies their status of amplification or deletion (Supplementary Fig. 12).

Finally, we used the integer-linear programming (ILP) formulation of HATCHet to assess the convergence of the related coordinate-descent (CD) algorithm to an optimal solution for D-CACF. In fact, the variables of the ILP can be initialized with a known solution (Supplementary Method 4) and standard ILP solvers estimate the gap between the given solution and the optimal solution by seeking for upper/lower bounds on the objective function[66]. As such, we applied and initialized the ILP with the solutions found by CD for all the 128 samples and found that the gap was $< 5\%$ in nearly all the cases. The combination of this result with the low errors obtained by HATCHet through CD (as described above) suggest that CD converged to an optimal solution in most of the cases. We also varied the number

of random restarts between 50-400 without noticing any clear difference, suggesting that such number of restarts are sufficient for convergence in most of the cases for instances with the size similar to our simulated samples. However, the gap substantially increased in several instances when considering a low number of restarts between 10-20.

4 Additional results on simulated data with free parameters

We ran all the methods on all the 256 samples with and without a WGD by considering free values of the parameters; as such, the values of the main parameters are not fixed and the methods directly infer those. To investigate different aspects of these solutions, we consider, as done above, different metrics described in Supplementary Note 2 to compare five current state-of-the-art methods, including Battenberg, TITAN, cloneHD, Canopy with FALCON, and ReMixT, with HATCHet, which was applied separately on single samples (single-sample HATCHet) or jointly on multiple samples from the same patient (HATCHet). THetA was excluded from this analysis as it does not automatically infer presence/absence of WGDs.

As described in Results, all methods exhibit worse performance but the performance of HATCHet remains robust and HATCHet continues to outperform the other methods (Fig. 2b and Supplementary Figs. 15–17). While the worse performance can be partially explained by erroneous predictions of tumor ploidy and WGDs, HATCHet is also the only method that exhibits a low average amplification-deletion error for most of the samples (Supplementary Fig. 18). The amplification-deletion error is generally independent from the correct prediction of a WGD and only assesses whether the total copy number of each segment has been correctly inferred as either amplified or deleted or unaffected (i.e. having a total copy number equal to 2 or 4 without or with a WGD, respectively). As such, a method that does not correctly identify the exact copy numbers because it wrongly predicts the presence/absence of a WGD can still obtain a low amplification-deletion error by correctly inferring the amplified-deleted segments. However, the high amplification-deletion error obtained by the existing methods for nearly all the samples indicates that these methods do not only fail the predictions of WGDs but also the inference of amplified-deleted segments. As all existing methods base the prediction of WGDs on the inferred value of tumor ploidy, these results highlight the challenges in modeling the dependency between tumor ploidy, copy numbers, and clone proportions (see comparison in Methods).

We assessed the tumor purity and tumor ploidy inferred for each sample by all methods in terms of their relative errors (Supplementary Figs. 13 and 14). The inference of tumor purity and tumor ploidy is crucial for existing methods, as they use these two parameters as the coordinates for CNA inference. However, HATCHet substantially outperforms all the other methods especially when jointly considering multiple samples. While HATCHet only exhibits a limited number of outlying samples with high errors, all existing methods exhibit an extensive number of samples with varying errors in the inference of both tumor purity and tumor ploidy. These results confirm the challenges in the inference of tumor purity and ploidy, and their being non-ideal coordinates for CNA inference as described in Methods. Interestingly, while Battenberg generally has clearly worse performance than cloneHD, Battenberg infers tumor ploidy and, especially, tumor purity with lower errors similar to those of cloneHD. These results highlight that the inference of tumor purity and tumor ploidy with a low error is not sufficient for accurate CNA inference, and the inference of copy numbers and proportions from these inferred values remain very challenging.

Finally, we assessed the predictions of WGDs for all methods in terms of precision and recall. We additionally considered in the comparison a consensus of the existing methods similarly to approaches adopted by recent pan-cancer studies[64]. While current methods as well as their consensus are biased towards one of the two predictions, HATCHet is the only method with high ($> 75\%$) precision and recall in the identification of both the presence and absence of a WGD (Fig. 2c). Moreover, we observed a high agreement among the samples with no WGD between the methods biased towards the absence of a WGD (Battenberg and Canopy with FALCON) and HATCHet, as well a high agreement among the samples with a WGD between the methods biased towards the presence of a WGD (TITAN, cloneHD, and ReMixT) and HATCHet (Supplementary Fig. 19).

The significantly lower performance of all existing methods when performing model selection as well as the high error-rate in the prediction of WGDs illustrate the challenges in selecting a solution using the coordinates of tumor purity and ploidy. While these coordinates are used by all these existing methods, HATCHet's model-selection criterion is based on the natural variables of the problem (i.e. the copy numbers A, B and the clone proportions U), enabling HATCHet to achieve the robust performance described above. To further investigate this point, we compared the fractional copy numbers inferred by HATCHet to those inferred by FALCON, one of the few existing methods that explicitly predict fractional copy numbers. We found that FALCON has much higher error rates than HATCHet (Supplementary Fig. 20a). The poor performance of FALCON relative to HATCHet is a result of different design choices. In particular, FALCON estimates a single value for the fractional copy number of each genomic segment, requiring that FALCON correctly determine whether a WGD has occurred or not. However, it is difficult to determine the occurrence of a WGD without knowledge of the clonal composition. In contrast, HATCHet computes a separate estimate of fractional copy numbers in the case with WGD and in the case without WGD, and uses a model selection criterion based on the inferred clonal structure to select between the two cases (Supplementary Figs. 20b,c). By deferring the selection of fractional copy numbers until after the copy-number deconvolution, HATCHet accounts for trade-offs in different solutions.

5 Comparing HATCHet's results with single-cell copy-number profiles

We further assessed the performance of HATCHet by comparing copy-number profiles derived by HATCHet on bulk tumor sequencing data with copy-number profiles from DOP-PCR single-cell DNA sequencing data from the same tumors. Specifically, we used HATCHet to analyze whole-exome sequencing data of 21 bulk tumor samples from 8 breast cancer patients: 12 bulk tumor samples from 4 breast cancer patients (P6, P9, P14, and P11) from Kim et al.[1] and 9 bulk tumor samples from 4 breast cancer patients (P4, P5, P6, and P10) from Casasent et al.[2]. We compared the copy numbers inferred by HATCHet jointly across the 2-3 bulk tumor samples from each patient with the copy-number profiles inferred from the DOP-PCR single-cell sequencing data from the same patient. More specifically, we applied HATCHet to jointly analyze the 2-3 samples of each patient with default values of all parameters and genomic bin size of 200kb on the available BAM files.

We observed a reasonable consistency between HATCHet's results and the single-cell copy-number profiles (Supplementary Figs. 21 and 22). Specifically, HATCHet correctly identified ≈93% of the clonal CNAs reported in the

single-cell copy-number profiles across all 8 patients. In 5/8 patients, HATCHet identifies a single tumor clone. In some of these patients, more than one distinct single-cell copy-number profile was reported, but most of these additional copy-number profiles were associated with a small fraction of cells ($<7\%$). Such low prevalence profiles are difficult to detect in bulk tumor samples, and may be present at different frequencies in the bulk samples, since each bulk sample and the single cells are distinct collections of cells from the same tumor. In the other 3/8 patients, HATCHet identified multiple tumor clones. In particular, HATCHet identified subclonal CNAs in $\approx 76\%$ of the genomic regions where the single-cell copy-number profiles exhibit different copy numbers across cells. In most of these regions, HATCHet correctly identified the copy numbers of the most prevalent tumor clone. While HATCHet is unable to identify all subclonal CNAs found in the single-cell profiles, there are notable limitations in this comparison. First, identification of CNAs from whole-exome sequencing data is much more challenging than from whole-genome sequencing data since whole-exome sequencing targets $<2\%$ of the genome. Second, the clonal composition of the bulk samples and the single-cell samples may be different. Third, the published DOP-PCR copy-number profiles are particularly noisy as DOP-PCR sequencing has very low coverage per cell ($<0.3\times$).

6 Experimental setup for cancer data

We used HATCHet to analyze two published whole-genome, multi-sample tumor sequencing datasets: a prostate cancer dataset[3] (Supplementary Fig. 23) and a pancreas cancer dataset[5] (Supplementary Fig. 24). First, the prostate cancer dataset comprises 10 patients (A10, A12, A17, A21, A22, A24, A29, A31, A32, and A34) with 50 samples. We excluded one sample (A21-F) from this dataset because it exhibits extremely outlying values of RDR and BAF. In the previous analysis[3], Battenberg has been applied to each sample of the prostate cancer dataset and the corresponding results are publicly available[3]. Second, the pancreas cancer dataset comprises 4 patients (Pam01, Pam02 Pam03, and Pam04) with 35 samples. In the previous analysis[5], Control-FREEC[16] has been applied to all these samples of this dataset assuming that tumor ploidy is equal to 2 but the corresponding results are not publicly available. We thus obtained the results of Control-FREEC via direct communication with the authors of Makohon et al.[5].

We applied HATCHet jointly considering all the samples from each patient and using the default values of all parameters. In particular, we used a maximum number of clones up to 8 for every patient as this was clearly sufficient in all cases. We did not choose a specific value for the maximum copy number c_{max} but HATCHet automatically chose c_{max} as the nearest integer that is higher or equal than the fractional copy number of every cluster s, i.e. $c_{\text{max}} = \max_{1 \leq s \leq m} \lceil f_{s,p} \rceil$ for every sample p. Additionally, we dealt with samples with outlying and noisy values of RDRs and BAFs by increasing the minimum clone proportion u_{min}; more specifically, we increased u_{min} from 0.03 up to 0.15 whenever we observed results with several clone proportions that are identical to the given u_{min} as this event may possibly indicate overfitting. All the executions of HATCHet have been run on a computer server equipped with Intel(R) Xeon(R) CPU E5-2690 and terminated in less than 8 hours, most of those were needed for obtaining RDRs and BAFs from all samples.

7 Comparing fractions of the genome with CNAs and subclonal CNAs

We compared the fractions of the genome with CNAs and subclonal CNAs reported by Battenberg and Control-FREEC in published results with those inferred by HATCHet. In this section, we formally define the computation of these fractions and we describe the corresponding results that we obtained when computing these values for all the samples of the prostate and pancreas cancer datasets.

First, the fraction of the genome with CNAs is defined as the fraction of the genome with a copy-number state different from the copy-number state (θ, θ) with $\theta = 1$ when there is no WGD and $\theta = 2$ when there is a WGD. More specifically, we compute the fraction of the genome with CNAs $\mathcal{M}_p$ in every sample p as

$$\mathcal{M}_p = \frac{1}{L} \sum_{\substack{1 \leq s \leq m, \exists i \; u_{i,p} > 0: \\ (a_{s,i}, b_{s,i}) \neq (\theta, \theta)}} \ell_s \tag{94}$$

when considering the allele-specific copy numbers A, B inferred by HATCHet. The fraction of the genome with CNAs can be equivalently computed when considering the results of Battenberg and Control-FREEC. However, as Control-FREEC only infers total copy numbers, we computed the fraction of the genome with CNAs both by identifying genomic regions with total copy number equal to 2θ (Control-FREEC) and by splitting the total copy numbers into the allele-specific copy numbers that better fit the observed BAFs (allelic Control-FREEC). While HATCHet and Battenberg infer very similar fractions of the genome with CNAs across all samples (Supplementary Fig. 30a), HATCHet infers a substantially higher fraction of the genome with CNAs than Control-FREEC on several samples of 3 over 4 patients (Pam02, Pam03, and Pam04) when both considering total and allele-specific copy numbers (Supplementary Fig. 34a).

Second, the fraction of the genome with subclonal CNAs is the fraction of the genome belonging to a sample-subclonal cluster relative to the fraction $\mathcal{M}_p$ of the genome with CNAs. Thus, we compute the fraction of the genome with subclonal CNAs in every sample p as

$$\frac{\frac{1}{L} \sum_{\substack{1 \leq s \leq m, \exists i,j \; u_{i,p}, u_{j,p} > 0: \\ (a_{s,i}, b_{s,i}) \neq (a_{s,j}, b_{s,j})}} \ell_s}{\mathcal{M}_p} \tag{95}$$

when considering the allele-specific copy numbers A, B inferred by HATCHet. The fraction of the genome with subclonal CNAs can be equivalently computed when considering the results of Battenberg and Control-FREEC. Similarly to the previous case, we computed the fraction of the genome with subclonal CNAs for Control-FREEC by both considering the total copy numbers (Control-FREEC) and the allele-specific copy numbers obtained according to the observed BAFs (allelic Control-FREEC). While Battenberg identifies a high fraction of the genome with subclonal CNAs in every sample of the prostate cancer dataset, HATCHet identifies several samples comprising a single tumor clone and a corresponding fraction equal to zero (Supplementary Fig. 30b). Conversely, while Control-FREEC does not infer subclonal CNAs in any sample of the pancreas cancer dataset, HATCHet identifies several samples having a high fraction of the genome with subclonal CNAs (Supplementary Fig. 34b). Interestingly, most of the samples where HATCHet identifies subclonal CNAs (Supplementary Fig. 34b) correspond to samples where Control-FREEC infers a

fraction of the genome with CNAs substantially lower than HATCHet (Supplementary Fig. 34a); this result suggests that Control-FREEC classifies as noisy diploid regions a high fraction of subclonal CNAs.

8 Measuring data fitting and number of parameters

We compared how well the results inferred by HATCHet and those reported in published results fit the data in terms of RDR and we simultaneously counted the corresponding number of used parameters. To measure the data fitting, we consider the relative error between the observed RDR and the estimated RDR that we obtain from the inferred copy numbers and proportions. More specifically, we do this in two steps when considering the results of HATCHet. First, we obtain the fractional copy number $\hat{f}_{t,p}$ of each bin t by Supplementary Equation 5 given the total copy numbers $c_{s,1}, \ldots, c_{s,n}$ and clone proportions $u_{1,p}, \ldots, u_{n,p}$ inferred by HATCHet for every cluster s in each sample p. Second, we estimate the related RDR $\hat{r}_{t,p} = \frac{\hat{f}_{t,p}}{\gamma_p}$ by applying Theorem 1 to compute the corresponding scaling factor γ_p. We equivalently estimate the RDR $\hat{r}_{t,p}$ related to the copy numbers and proportions inferred by Battenberg and Control-FREEC. We hence compute the relative error between the observed RDR $\overline{r}_{t,p}$ and $\hat{r}_{s,p}$ for every bin t in each sample p as

$$\frac{|\overline{r}_{t,p} - \hat{r}_{t,p}|}{\overline{r}_{t,p}}. \tag{96}$$

In the rest of the section, we first describe the results on the prostate cancer dataset and next on the pancreas cancer dataset.

8.1 Data fitting and number of parameters in the prostate cancer dataset

We computed the relative error between the observed and estimated RDRs when considering the copy numbers and proportions correspondingly inferred by HATCHet and Battenberg for all the samples of the prostate cancer dataset. In addition, we simultaneously counted the number of parameters used in the corresponding solutions. As HATCHet and Battenberg similarly predict the absence or presence of a WGD in most of the samples, we applied Theorem 1 to compute the corresponding scaling factor γ_p by selecting the two largest sample-clonal clusters for which HATCHet and Battenberg infer the same copy-number state. We did this to limit the biases that may result from considering different values of γ_p due to noise and we thus consider the same value of γ_p for HATCHet and Battenberg. We observed that HATCHet achieves the same average error of Battenberg for the samples of 8/10 patients and an average error lower than Battenberg for the samples of the remaining 2/10 patients (Supplementary Fig. 31). Moreover, HATCHet resulted in a lower maximum error in most of the samples, while Battenberg decreases the maximum errors only in two patients where the improvement is limited or is achieved at the cost of a worse average error (Supplementary Fig. 31).

We next consider the number of corresponding parameters for HATCHet and Battenberg in the prostate cancer dataset. First, the number of parameters used by HATCHet corresponds to the number of allele-specific copy numbers for all clusters in A, B and the number of clone proportions in U for all samples. Second, the number of parameters used by Battenberg corresponds to the number of allele-specific copy numbers for every segment (that are at most 4 for each segment as Battenberg assumes there are at most 2 distinct copy-number states per segment in tumor clones)

and the number of corresponding proportions, that are specific for each segment as Battenberg models each segment independently (Supplementary Fig. 28). We observed that HATCHet describes the data with at least 6 times less parameters than Battenberg (Supplementary Fig. 29), even though HATCHet explains the data as well as Battenberg, or better.

8.2 Data fitting and number of parameters in the pancreas cancer dataset

We computed the relative error between the observed and estimated RDRs when considering the copy numbers and proportions correspondingly inferred by HATCHet and Control-FREEC for all the samples of the pancreas cancer dataset. In addition, we simultaneously counted the number of parameters used in the corresponding solutions. As Control-FREE has been applied assuming the absence of a WGD while HATCHet predicts a WGD in most of the samples, we apply Theorem 1 by selecting the two largest sample-clonal clusters with copy-number states equal to either $(2, 2)$ and $(2, 1)$ or $(2, 2)$ and $(2, 0)$ for HATCHet and the two largest sample-clonal clusters with total copy numbers equal to either 2 and 3 or 2 and 1 for Control-FREEC. We observed that HATCHet consistently achieves a lower error between the observed and estimated RDRs than Control-FREEC on the samples of all the patients (Supplementary Fig. 35a); in fact, the solutions of Control-FREEC exhibit a substantially higher number of bins with higher errors than HATCHet (Supplementary Fig. 35b).

We next consider the number of corresponding parameters for HATCHet and Control-FREEC in the pancreas cancer dataset. First, as described before, the number of parameters used by HATCHet corresponds to the number of allele-specific copy numbers for all clusters in A, B and the number of clone proportions in U for all samples. Second, the number of parameters used by Control-FREEC simply corresponds to the number of total copy numbers for all segments as Control-FREEC assumes there is a single tumor clone in each sample. We observed that HATCHet describes the data with at least 3 times less parameters than Control-FREEC (Supplementary Fig. 36), even though HATCHet explains the data better than Control-FREEC.

9 Comparing HATCHet with Battenberg and ReMixT on prostate cancer patients

We evaluated ReMixT's performance on 17/49 the prostate cancer samples from Gundem et al.[3] that we analyzed in our manuscript: A10-C, A12-C-D, A17-D, A21-G-H, A24-C-D-E, A31-A-D-E-F, A34A-C-D, and A32-E. More specifically, ReMixT was provided with the breakpoints inferred in the previous analysis[3]. We found that ReMixT identified subclonal CNAs and shared subclonal CNAs across all samples, similar to Battenberg (Supplementary Fig. 32). More specifically, ReMixT identified subclonal CNAs in a higher fraction of the genome than both HATCHet and Battenberg in most of the samples (Supplementary Fig. 32a), and ReMixT also identified shared subclonal CNAs in nearly all the samples with higher fractions than those reported by Battenberg (Supplementary Fig. 32b). Notably, ReMixT identifies a fraction of the genome with subclonal CNAs closer to HATCHet's results (average difference of 18.6%) than Battenberg's results (average difference of 34.3%) in samples where HATCHet identifies subclonal CNAs (Supplementary Fig. 32b).

10 HATCHet enables the identification of tumor clones shared across samples

We used HATCHet to investigate the presence of tumor clones that are present in more than one sample from the same patient. Previous analyses of SNVs in the prostate and pancreas cancer datasets reached different conclusions on the presence of such shared tumor clones: in the prostate dataset, Gundem et al.[3] reported that 27/49 samples had two or more clones that were also present in other samples from the same patient, providing evidence of polyclonal migrations between metastatic sites. In contrast, in the pancreas dataset, Makohon-Moore et al.[5] reported that all samples contained at most one tumor clone that was shared with another sample from the same patient, supporting monoclonal migrations between metastases. An important question is whether these differences are a result of variability across patients, variability across cancer types, or differences in the bioinformatic approaches used in the two studies.

HATCHet reported that 14/49 samples from five prostate cancer patients contain at least two tumor clones that are also present in other samples from the same patient (Supplementary Fig. 38). Notably, in three of these patients (A22, A31, and A32), HATCHet reported that at least two samples share at least two identical tumor clones, providing evidence of polyclonal migrations (Supplementary Fig. 43) between these samples. These same three patients were the only patients from this cohort that were reported to have polyclonal migrations in an independent analysis of this same dataset[4]. In contrast, the Battenberg-derived CNAs reported in Gundem et al.[3] suggest a more complicated story with polyclonal migrations in 9/10 patients (Supplementary Fig. 37). However, based on analysis of SNVs, Gundem et al.[3] reported polyclonal migrations in only 5 patients: the 3 identified by HATCHet plus A24 and A34. Thus, HATCHet's copy-number analysis is more consistent with the polyclonal migrations reported in Gundem et al.[3] than the copy-number analysis from the same publication.

HATCHet reported that 13/35 samples from all four pancreas cancer patients contain at least two tumor clones that are also present in other samples from the same patient (Supplementary Fig. 39). For example, in patient Pam01 HATCHet identified two clones in a lymph node metastasis sample Pam01_NoM1 (Fig. 4b), one of which is found in a liver metastasis (sample Pam01_LiM2 in Fig. 4c) and the other found in a different liver metastasis (sample Pam01_LiM1 in Fig. 4d). This result reveals a crucial role for the lymph node in the metastatic spread of this tumor, a finding consistent with standard models of metastasis[67] but in contradiction to recent studies from other cancer types that suggest lymph nodes do not actively participate in the metastatic process[68,69]. In the other three patients (Pam02, Pam03, and Pam04), HATCHet reported that at least two samples share at least two identical tumor clones, providing evidence of polyclonal migrations (Supplementary Fig. 43). This result is consistent with the reports of polyclonal migrations in mouse models of pancreatic tumors[6].

11 Analysis of heterogeneity across samples through sample-specific copy-number states

The published analysis of both the prostate and pancreas cancer patients reported that most SNVs are shared across samples from the same patient. This observation led to the conclusion that there is limited heterogeneity between the samples, with several samples sharing the same set of SNV-derived tumor clones[3,5]. However, the CNAs previously computed in such analyses do not support the reported limited heterogeneity. To quantify this discordance,

we identified sample-specific copy-number states, i.e. copy-number states (a, b), or CNAs, that are unique to a single sample (Supplementary Fig. 40a). We observed that the published CNAs for the prostate and pancreas cancer datasets contain sample-specific copy-number states in every sample in a high fraction of the genome (Supplementary Figs. 40b,c and 41); these correspond to many, large sample-specific CNAs distributed across all chromosomes (Supplementary Fig. 42). This suspicious result is likely due to the independent analysis of each sample performed by the CNA methods used in previous studies, i.e. Battenberg and Control-FREEC. Moreover, we showed in the analysis of subclonal CNAs that Battenberg is prone to overfitting and the extensive presence of subclonal CNAs may also explain the extensive presence sample-specific copy-number states. In contrast, HATCHet identified sample-specific copy-number states in only a few samples, a consequence of HATCHet's joint analysis of tumor clones across samples. Overall, the CNAs inferred by HATCHet support the previously-reported limited heterogeneity across samples (especially metastatic samples) better than published CNAs. In the rest of this section, we first provide the details of the measure used to quantify heterogeneity across samples, and next we analyze the corresponding results on the prostate and pancreas patients.

We introduce a quantitative measure for analyzing the heterogeneity across samples from the same patient when considering the CNAs inferred by HATCHet and published in the previous analysis of both the prostate and pancreas cancer datasets. Different CNAs are generally inferred in different samples from the same patient as distinct tumor clones may be present in each sample. As such, we define the measure based on sample-specific copy-number states, which are copy-number states that are unique to a single sample for a certain segment and correspond to sample-specific CNAs (Supplementary Fig. 40a). In particular, let A, B be the allele-specific copy numbers and U be the clone proportions inferred by HATCHet, and let θ be equal to either 1 or 2 without or with a WGD, respectively. We say that a cluster s has a sample-specific copy-number state $(a_{s,i}, b_{s,i})$ in sample p if $(a_{s,i}, b_{s,i}) \neq (\theta, \theta)$ for a tumor clone i with $u_{i,p} > 0$ and if there is no tumor clone j (including $i = j$) present in a different sample q with $(a_{s,i}, b_{s,i}) = (a_{s,j}, b_{s,j})$. Similarly to the computation of the fraction of the genome with subclonal CNAs (Supplementary Note 7), we thus compute the fraction of the genome with sample-specific copy-number states relative to the fraction $\mathcal{M}_p$ of the genome with CNAs in every sample p as

$$\frac{\frac{1}{L} \sum_{\substack{1 \leq s \leq m, \nexists i,j,q \; u_{i,p}, u_{j,q} > 0, p \neq q: \\ (a_{s,i}, b_{s,i}) = (a_{s,j}, b_{s,j}), (a_{s,i}, b_{s,i}) \neq (\theta, \theta)}} \ell_s}{\mathcal{M}_p}. \tag{97}$$

We equivalently compute the same fraction when considering the copy-number states and proportions inferred by Battenberg and Control-FREEC from the samples of the prostate and pancreas cancer datasets, respectively.

We compared the fractions of the genome with sample-specific copy-number states inferred by HATCHet and Battenberg from the samples of the prostate cancer dataset (Supplementary Fig. 41a), and those inferred by HATCHet and Control-FREEC from the samples of the pancreas cancer dataset (Supplementary Fig. 41b). Battenberg and Control-FREEC identify sample-specific states in substantial fractions of all samples (Supplementary Figs. 40b,c). In addition, we observed that both Battenberg and Control-FREEC infer large genomic regions with sample-specific copy-number states in all chromosomes of every patient (Supplementary Figs. 42a,b). These results appear to be clearly in contradiction with the limited heterogeneity across samples that has been reported in the published SNV analyses[3,5]. In contrast,

the results of HATCHet support the limited heterogeneity across samples; in fact, HATCHet only identifies a limited number of samples with sample-specific states in the prostate and pancreas datasets (Supplementary Figs. 40b,c). This demonstrates the advantage of HATCHet's joint analysis of multiple samples.

The results of HATCHet also suggest that large CNAs are mainly acquired before the metastatic phase of tumor progression, as suggested by recent models of CNAs[70]. More specifically, the absence of sample-specific copy-number states in most of the samples suggests that, at least in these cases, distinct tumor clones acquired CNAs and, subsequently, migrate to seed metastases in different sites without acquiring additional CNAs. In contrast, the extensive presence of sample-specific copy-number states inferred by both Battenberg and Control-FREEC suggests a progressive accumulation of CNAs during tumor evolution.

12 Assessing the presence of subclonal CNAs through clonality distance

We introduce a quantitative measure, called clonality distance, to analyze the presence of subclonal CNAs directly from the observed RDRs and BAFs. We applied the clonality distance to investigate the support for the CNAs and WGDs inferred by HATCHet from the samples of the prostate and pancreas cancer patients. Specifically, the clonality distance is computed for a genomic region and aims to estimate the distance between the region and the closest potential sample-clonal cluster in terms of RDR and BAF, where a sample-clonal cluster is a cluster with the same copy-number state in all tumor clones. Intuitively, the higher the distance is, the more likely is that the region is part of a sample-subclonal cluster and corresponds to a subclonal CNA.

12.1 Computation of clonality distance

We now describe the computation of the clonality distance for ζ bins of fixed size (equal to 50kb for all the results in this work) that we obtained by partitioning the reference genome and considering the RDR $\overline{r}_{t,p}$ and the BAF $\overline{\beta}_{t,p}$ for each bin t in every sample p as described in Supplementary Method 2. Suppose to know the tumor purity μ_p of every sample p, we can compute the RDR and BAF of any potential sample-clonal cluster by applying Theorem 1. More specifically, we aim to compute the RDR $\mathfrak{r}_{\alpha,\iota,\mu_p}$ and the BAF $\mathfrak{b}_{\alpha,\iota,\mu_p}$ of any potential sample-clonal cluster with copy-number state (α, ι), which is a cluster with the same copy-number state in all tumor clones present in p, i.e. $\forall i \in \{2, \ldots, n\}$ with $u_{i,p} > 0$, $(a_{s,i}, b_{s,i}) = (\alpha, \iota)$. We do this in two steps. First, we identify either the cluster s with copy-number state $(1, 1)$ when there is no WGD or the cluster s with copy-number state $(2, 2)$ when there is a WGD as described in Supplementary Method 3. As such, we compute the scaling factor $\gamma(\mu_p)$ associated to the known μ_p by combining Supplementary Equations 5 and 8 in the following

$$\gamma(\mu_p) = \frac{2\theta\mu_p + 2(1 - \mu_p)}{r_{s,p}} \tag{98}$$

where $r_{s,p}$ is the RDR of s in p and either $\theta = 1$ when there is no WGD or $\theta = 2$ when there is a WGD. Second, we explicitly compute $\mathfrak{r}_{\alpha,\iota,\mu_p}$ by combining Supplementary Equations 5 and 8 as

$$\mathfrak{r}_{\alpha,\iota,\mu_p} = \frac{(\alpha + \iota)\mu_p + 2(1 - \mu_p)}{\gamma(\mu_p)} \tag{99}$$

and we compute $\mathfrak{b}_{\alpha,\iota,\mu_p}$ by combining Supplementary Equations 5, 11, and 13 as

$$\mathfrak{b}_{\alpha,\iota,\mu_p} = \frac{\iota\mu_p + 1 - \mu_p}{(\alpha + \iota)\mu_p + 2(1 - \mu_p)} \tag{100}$$

assuming that $\iota \leq \alpha$ without loss of generality.

Finally, we compute the clonality distance for a genomic bin t in a sample p. When the tumor purity μ_p is given, we define the clonality distance $d(p, t, \mu_p)$ for every bin t in sample p as the Euclidean distance between t and the closest tumor-clonal cluster in terms of RDR and BAF as follows

$$d(p, t, \mu_p) = \min_{\alpha,\iota \in \{0,\Xi\}} \sqrt{(\overline{r}_{t,p} - \mathfrak{r}_{\alpha,\iota,\mu_p})^2 + (\overline{\beta}_{t,p} - \mathfrak{b}_{\alpha,\iota,\mu_p})^2} \tag{101}$$

by assuming for simplicity that the allele-specific copy numbers $\alpha \leq \Xi$ and $\iota \leq \Xi$ for every potential sample-clonal cluster. As the tumor purity μ_p is unknown, we choose the value υ_p of μ_p that minimizes the sum of the distances $d(p, t, \upsilon_p)$ for every bin t. Assuming we aim to estimate υ_p with a maximum error of 0.01, we have the following

$$\upsilon_p = \operatorname*{argmin}_{\mu \in \{0, 0.01, \ldots, 1\}} \sum_{1 \leq t \leq \zeta} d(p, t, \mu). \tag{102}$$

As such, we compute the clonality distance $\lambda(t, p)$ for every bin t in sample p as

$$\lambda(p, t) = d(p, t, \upsilon_p). \tag{103}$$

12.2 Analysis of the clonality distance

In principle, values of the clonality distance higher than zero correspond to genomic regions with subclonal CNAs. In practice, the presence of noise in the data and their variance affect the values of the clonality distance; consequently, small differences among the values of the clonality distance may not be indicative for the presence of subclonal CNAs. As such, we only consider samples with bins that exhibit clear variations in the values of the clonality distance. In particular, we computed the clonality distance $\lambda(p, t)$ for every bin t in every sample p by assuming either the absence or presence of a WGD for all the patients in the prostate cancer dataset (Supplementary Fig. 48a) and in the pancreas cancer dataset (Supplementary Fig. 48b).

We first observed that the values of the clonality distance support the presence of subclonal CNAs in the samples where HATCHet infers multiple tumor clones (Supplementary Figs. 44 and 45). In particular, we observed a clear difference in terms of clonality distance between samples with multiple tumor clones and those with a single tumor clone inferred by HATCHet. When considering both the prostate and pancreas cancer datasets, samples where multiple tumor clones have been identified by HATCHet exhibit a large amount of bins with high values of the clonality distance, corresponding to subclonal CNAs. In contrast, samples where a single tumor clone has been identified by HATCHet mostly exhibit only bins with low values of the clonality distance.

We next observed that the values of the clonality distance support the WGDs predicted by HATCHet (Supplementary Figs. 48a and 48b). Similarly to the previous case, we observed a clear difference in terms of clonality distance between samples with and without a WGD according to the results of HATCHet. We observed in all samples from the

patients where HATCHet identifies a WGD (prostate cancer patients A29, A31, and A32, and pancreas cancer patients Pam02, Pam02, and Pam04) a clear trade-off between subclonal CNAs and WGDs, as opposed to the other patients. More specifically, all these samples exhibit a large amount of bins with high values of the clonality distance when assuming the absence of a WGD, while nearly all bins have low values when assuming the presence of a WGD. This result confirms the trade-off that we expect in samples with WGDs, where the high number of clusters correspond to either sample-subclonal cluster when there is not a WGD (i.e. subclonal CNAs) or sample-clonal clusters when there is a WGD (Supplementary Fig. 1).

13 Analysis of divergent predictions of WGD across multiple samples

We examined the prediction of WGDs on the prostate and pancreas cancer datasets. The previously published analyses of these datasets reached opposite conclusions regarding the landscape of WGDs in these tumors. Gundem et al.[3] reported WGDs in 12 samples of 4 prostate cancer patients (A12, A29, A31, and A32). In contrast, Makohon-Moore et al.[5] did not evaluate the presence of WGDs in the pancreas cancer samples, despite reports of high prevalence of WGD in pancreas cancer[71]. We investigated whether HATCHet's analysis would confirm or refute the different prevalence of WGD reported in the previous studies.

On the prostate cancer dataset, there is strong agreement between WGD predictions from Battenberg and HATCHet, with discordance on only 2 of 49 samples (Supplementary Figs. 46a,b). Note that Battenberg does not explicitly state whether a WGD is present in a sample, and thus we used the criterion from previous pan-cancer analysis[7,34,35,64,72] that a tumor sample with ploidy > 3 corresponds to WGD. Since Battenberg's solutions were manually chosen from many alternatives in the published analysis, the strong agreement between these predictions is a positive indicator for HATCHet's automated model selection. The two discordant samples, A12-C and A29-C, are single samples from patients A12 and A29, respectively (Supplementary Figs. 46a,b). Battenberg predicted a WGD only in A12-C and no WGD in the other samples from this patient. Conversely, Battenberg predicted no WGD in A29-C but a WGD in the other sample from this patient. However, the divergent predictions of WGD in only one sample of these patients is not well-supported by the data. In particular, the observation of a large number of distinct clusters of genomic bins (i.e. distinct copy-number states) in a sample has two reasonable explanations: subclonal CNAs or a WGD (Supplementary Fig. 1). Since Battenberg analyzes each sample independently it may choose a different explanation (subclonal CNAs vs. WGD) for the large number of clusters observed in each sample from the same patient. In some cases like sample A12-C, Battenberg predicts both subclonal CNAs and WGD (Supplementary Figs. 46c and 47a). Since both subclonal CNAs and WGDs increase the total number of copy-number states available to explain distinct clusters, they will generally provide a better fit to the data. However, there is a danger of overfitting since both WGD and subclonal CNAs increase the number of parameters in the model. In contrast, HATCHet jointly analyzes multiple samples and predicts the absence/presence of a WGD consistently across all samples from the same patient (Supplementary Figs. 46a,b): no WGD in all samples of patient A12 and a WGD in all samples of patient A29. Moreover, HATCHet integrates the choice of WGD into the model selection procedure, providing a simpler explanation of the data (with only subclonal CNAs or only WGD) with an equally good fit to the observed RDRs and

BAFs (Supplementary Figs. 46d, 47b, and 48a).

On the pancreas dataset, the published analysis excluded the possibility of WGDs and assumed that tumor ploidy is always equal to 2 (Supplementary Fig. 33). Instead, HATCHet predicted a WGD in all 31 samples from three of the four patients (Fig. 5a and Supplementary Fig. 49a). These results are consistent with recent reports of the high frequency of WGD ($\sim$45%) and massive rearrangements in pancreatic cancer[20,71], and also supported by additional analyses (Supplementary Fig. 48b). All 31 samples from the 3 patients with a WGD display a large number of clusters of genomic regions with clearly distinct values of RDR and BAF. When jointly considering all samples from the same patient, these clusters are clearly better explained by the occurrence of a WGD (Fig. 5b) than by the presence of many subclonal CNAs, as the latter would result in the unlikely presence of distinct tumor clones with the same proportions in all samples (Supplementary Fig. 49b). By directly evaluating the trade-off between subclonal CNAs and WGDs in the model selection, HATCHet makes more reasonable predictions of the occurrence of WGDs.

14 Analysis of somatic mutations

We further assessed the copy numbers and proportions inferred by HATCHet and existing methods by using an orthogonal analysis of somatic mutations, including somatic SNVs and small indels. We used VarScan 2 (v2.3.9)[73,74] to call, process, and filter somatic mutations from every sample of the prostate and pancreas cancer datasets, using the default pipeline described at `http://varscan.sourceforge.net` and retaining only those classified with high confidence and high frequency (VAF>0.2). As such, we identified $\approx$10 600 mutations on average across the samples of the prostate cancer dataset (Supplementary Fig. 50a) and $\approx$9 000 mutations on average across the samples of the pancreas cancer dataset (Supplementary Fig. 50b). In the next two subsections, we first use somatic mutations to compare the results of HATCHet with those from previous analyses and next we interpret the observed VAFs based on the HATCHet's results: we show that both these analyses provide further evidence for the results of HATCHet.

14.1 Comparison between HATCHet and existing methods

We propose two measures based on the orthogonal DNA sequencing signals from somatic mutations to compare the results of HATCHet with published results. Specifically, we observe the VAF $\psi_{e,p}$ of every mutation e from every sample p as the fraction of reads in p harboring e at the corresponding locus (Supplementary Figs. 53 and 55). When the mutation e is located in a genomic region belonging to cluster s, we thus model the *predicted* VAF $\overline{\psi}_{e,p}$ of e in p similarly to the BAF $\beta_{s,p}$ in Supplementary Method 1 as the following

$$\overline{\psi}_{e,p} = \frac{\dot{f}_{s,p,e}}{f_{s,p}} \tag{104}$$

where is $\dot{f}_{s,p,e}$ is the mutated fractional copy number correspondingly equal to

$$\dot{f}_{s,p,e} = \sum_{2 \leq i \leq n} \dot{c}_{s,i,e} u_{i,p} \tag{105}$$

where $\dot{c}_{s,i,e}$ is the related mutated total copy number for every clone i, i.e. the number of copies of s that harbor the mutation e over the total copy number $c_{s,i}$.

As a first measure, we compared the observed VAF $\psi_{e,p}$ with the predicted VAF $\overline{\psi}_{e,p}$ that we estimate according to the copy numbers and proportions inferred by HATCHet and reported in published results. To estimate the predicted VAF $\overline{\psi}_{e,p}$ of a somatic mutation from the inferred copy numbers and proportions, we need to infer the unknown mutated total copy number $\dot{c}_{s,i,e}$ for every mutation e in cluster s of clone i. We do this by choosing the values that allow to obtain the better estimation $\overline{\psi}_{e,p}$ of the observed VAF $\psi_{e,p}$. Moreover, we assume that every mutation occurs in the same genomic position at most once, similarly to standard models based on the infinite-site assumption[27,28,75], and we hence constrain the mutation to be present only in the copies of one allele of s, i.e. $\dot{c}_{s,i,e} \leq \max\{a_{s,i}, b_{s,i}\}$. Given the allele-specific copy numbers A, B and clone proportions U inferred by HATCHet, we thus choose the mutated copy number $\dot{c}_{s,i,e}$ for every mutation e in a cluster s in order to correspondingly obtain the closest estimation to the corresponding observed VAF $\psi_{e,p}$ in every sample p. Thus, we have the following

$$\dot{c}_{s,2,e}, \dots, \dot{c}_{s,n,e} = \operatorname*{argmin}_{\substack{\tilde{c}_2,\dots,\tilde{c}_n: \\ \tilde{c}_i \leq \max\{a_{s,i}, b_{s,i}\}, 2 \leq i \leq n}} \left| \frac{\sum_{2 \leq i \leq n} \tilde{c}_i u_{i,p}}{\sum_{1 \leq i \leq n} (a_{s,i} + b_{s,i}) u_{i,p}} - \psi_{e,p} \right| \tag{106}$$

where the VAF $\overline{\psi}_{e,p}$ is estimated as $\frac{\dot{f}_{s,p,e}}{f_{s,p}}$ according to the model in Supplementary Equation 104, the fractional copy number is given by $f_{s,p} = \sum_{1 \leq i \leq n} (a_{s,i} + b_{s,i}) u_{i,p}$, and the mutated fractional copy number $\dot{f}_{s,p,e}$ is computed as in Supplementary Equation 105. We thus obtain the predicted VAF $\overline{\psi}_{e,p}$ as

$$\overline{\psi}_{e,p} = \frac{\sum_{2 \leq i \leq n} \dot{c}_{s,i,e} u_{i,p}}{\sum_{1 \leq i \leq n} (a_{s,i} + b_{s,i}) u_{i,p}} \tag{107}$$

where the mutated copy numbers $\dot{c}_{s,2,e}, \dots, \dot{c}_{s,n,e}$ are computed as in Supplementary Equation 106. The mutated copy numbers and the predicted VAF can be equivalently computed by considering the results inferred by Battenberg and Control-FREEC. Moreover, we computed the mutated copy numbers independently for each sample as only HATCHet jointly model multiple samples.

We assessed the results inferred by HATCHet and the ones reported in published analysis by comparing the observed and predicted VAF. More specifically, we computed the relative error between the observed VAF $\psi_{e,p}$ and the predicted VAF $\overline{\psi}_{e,p}$ of every somatic mutation e in every sample p of the prostate and pancreas cancer datasets when considering both the copy numbers and proportions inferred by HATCHet and published analysis as

$$\frac{|\psi_{e,p} - \overline{\psi}_{e,p}|}{\psi_{e,p}}. \tag{108}$$

The copy numbers and proportions inferred by HATCHet allow to consistently obtain better estimations than the ones inferred by Battenberg across the samples of all prostate cancer patients (Supplementary Fig. 51) and than the ones inferred by Control-FREEC across the samples of all pancreas cancer patients (Supplementary Fig. 52). Control-FREEC only infers total copy numbers and we hence computed the mutated total copy numbers both without constraining the mutation to a single allele (Control-FREEC) and by considering the allele-specific copy numbers that better fit to the observed BAF (allelic Control-FREEC), similarly to Supplementary Note 7.

The observed VAF of the mutations in different samples is affected by variance and noise as the total number of sequencing reads that cover each mutation varies. To take into account these variations and to directly consider

the read counts, we compute a standard 95% confidence interval (CI) obtained using a binomial model with a beta distribution as a prior[27,28]. As a second measure, we thus say that every mutation e in a sample p is explained by the inferred copy numbers and proportions when the corresponding predicted VAF $\overline{\psi}_{e,p}$ is contained in the associated CI. We applied this test to every mutation e in each sample p of the prostate and pancreas cancer datasets by considering the copy numbers and proportions inferred by HATCHet and Battenberg, and by HATCHet and Control-FREEC, respectively. As in the previous case, we showed that HATCHet outperforms Battenberg and Control-FREEC. First, HATCHet consistently explains more mutations than Battenberg across the samples of all prostate cancer patients but one (patient A29) where however the difference is limited (Fig 6b). Second, HATCHet explains substantially more mutations than Control-FREEC across the samples of all pancreas cancer patients (Fig 6c). Note that we observed similar results for Control-FREEC when either assigning the mutation to both alleles (Control-FREEC) or considering the allele-specific copy numbers that better fit the observed BAFs (allelic Control-FREEC).

14.2 The observed VAFs of somatic mutations support the results of HATCHet

We investigated the observed VAFs of somatic mutations to assess whether these observations support the results of HATCHet. First, we observed that the VAFs of somatic mutations in the samples where HATCHet predicts a WGD or massive rearrangements are generally lower than the ones observed in the samples where HATCHet does not predict a WGD (Supplementary Figs. 53 and 55). For example, the values of VAF are lower in all samples of the prostate cancer patient A29 and pancreas cancer patients Pam02, Pam03, and Pam04 where HATCHet predicts a WGD as well as in all samples of the prostate cancer patient A22 and pancreas cancer patient Pam01 where HATCHet predicts massive rearrangements. These observations support the results of HATCHet: in fact, the low values of VAF that we systematically observe in all these samples are compatible with the higher fractional copy number $f_{s,p}$ that we expect in these cases as only few copies of s may be mutated and the frequency is consequently lower (Supplementary Equation 104). The samples of patients A31 and A32, where HATCHet also predicts a WGD, apparently are two exceptions as the observed VAFs are not clearly lower than the VAFs in other patients. However, this difference is explained by the predominance of deletions in these patients (Supplementary Fig. 23) that result in fractional copy numbers lower than expected when a WGD occurs.

Finally, we analyzed the observed VAFs for all the somatic mutations unexplained by the copy numbers and clone proportions inferred by HATCHet (Supplementary Figs. 54 and 56). Interestingly, we observed that these unexplained mutations generally have lower values of the observed VAF than the other mutations in the samples of most of prostate cancer patients and pancreas cancer patient Pam04 (Supplementary Figs. 53 and 55). These findings are consistent with the presence of further distinct tumor clones that have the same CNAs but have distinct somatic mutations, which have been accumulated after CNAs during tumor evolution.

Supplementary References

[1] Kim, C. *et al.* Chemoresistance evolution in triple-negative breast cancer delineated by single-cell sequencing. *Cell* **173**, 879–893 (2018).

[2] Casasent, A. K. *et al.* Multiclonal invasion in breast tumors identified by topographic single cell sequencing. *Cell* **172**, 205–217 (2018).

[3] Gundem, G. *et al.* The evolutionary history of lethal metastatic prostate cancer. *Nature* **520**, 353 (2015).

[4] El-Kebir, M., Satas, G. & Raphael, B. J. Inferring parsimonious migration histories for metastatic cancers. *Nature Genetics* **50**, 718–726 (2018).

[5] Makohon-Moore, A. P. *et al.* Limited heterogeneity of known driver gene mutations among the metastases of individual patients with pancreatic cancer. *Nature Genetics* **49**, 358 (2017).

[6] Maddipati, R. & Stanger, B. Z. Pancreatic cancer metastases harbor evidence of polyclonality. *Cancer Discovery* **5**, 1086–1097 (2015).

[7] Carter, S. L. *et al.* Absolute quantification of somatic DNA alterations in human cancer. *Nature Biotechnology* **30**, 413 (2012).

[8] Van Loo, P. *et al.* Allele-specific copy number analysis of tumors. *Proceedings of the National Academy of Sciences* **107**, 16910–16915 (2010).

[9] Nik-Zainal, S. *et al.* The life history of 21 breast cancers. *Cell* **149**, 994–1007 (2012).

[10] Chen, H., Bell, J. M., Zavala, N. A., Ji, H. P. & Zhang, N. R. Allele-specific copy number profiling by next-generation DNA sequencing. *Nucleic Acids Research* **43**, e23–e23 (2014).

[11] Jiang, Y., Qiu, Y., Minn, A. J. & Zhang, N. R. Assessing intratumor heterogeneity and tracking longitudinal and spatial clonal evolutionary history by next-generation sequencing. *Proceedings of the National Academy of Sciences* **113**, E5528–E5537 (2016).

[12] Favero, F. *et al.* Sequenza: allele-specific copy number and mutation profiles from tumor sequencing data. *Annals of Oncology* **26**, 64–70 (2014).

[13] Ha, G. *et al.* TITAN: inference of copy number architectures in clonal cell populations from tumor whole-genome sequence data. *Genome Research* **24**, 1881–1893 (2014).

[14] Shen, R. & Seshan, V. E. FACETS: allele-specific copy number and clonal heterogeneity analysis tool for high-throughput DNA sequencing. *Nucleic Acids Research* **44**, e131–e131 (2016).

[15] Cun, Y., Yang, T.-P., Achter, V., Lang, U. & Peifer, M. Copy-number analysis and inference of subclonal populations in cancer genomes using Sclust. *Nature Protocols* **13**, 1488 (2018).

[16] Boeva, V. *et al.* Control-FREEC: a tool for assessing copy number and allelic content using next-generation sequencing data. *Bioinformatics* **28**, 423–425 (2011).

[17] Oesper, L., Mahmoody, A. & Raphael, B. J. THetA: inferring intra-tumor heterogeneity from high-throughput DNA sequencing data. *Genome Biology* **14**, R80 (2013).

[18] Oesper, L., Satas, G. & Raphael, B. J. Quantifying tumor heterogeneity in whole-genome and whole-exome sequencing data. *Bioinformatics* **30**, 3532–3540 (2014).

[19] Fischer, A., Vázquez-García, I., Illingworth, C. J. & Mustonen, V. High-definition reconstruction of clonal composition in cancer. *Cell Reports* **7**, 1740–1752 (2014).

[20] Notta, F. *et al.* A renewed model of pancreatic cancer evolution based on genomic rearrangement patterns. *Nature* **538**, 378 (2016).

[21] McPherson, A. W. *et al.* ReMixT: clone-specific genomic structure estimation in cancer. *Genome Biology* **18**, 140 (2017).

[22] Lander, E. S. & Waterman, M. S. Genomic mapping by fingerprinting random clones: a mathematical analysis. *Genomics* **2**, 231–239 (1988).

[23] Zaccaria, S., El-Kebir, M., Klau, G. W. & Raphael, B. J. The copy-number tree mixture deconvolution problem and applications to multi-sample bulk sequencing tumor data. In *International Conference on Research in Computational Molecular Biology*, 318–335 (Springer, 2017).

[24] Zaccaria, S., El-Kebir, M., Klau, G. W. & Raphael, B. J. Phylogenetic copy-number factorization of multiple tumor samples. *Journal of Computational Biology* **25**, 689–708 (2018).

[25] Li, H. *et al.* The sequence alignment/map format and samtools. *Bioinformatics* **25**, 2078–2079 (2009).

[26] Li, H. A statistical framework for SNP calling, mutation discovery, association mapping and population genetical parameter estimation from sequencing data. *Bioinformatics* **27**, 2987–2993 (2011).

[27] El-Kebir, M., Oesper, L., Acheson-Field, H. & Raphael, B. J. Reconstruction of clonal trees and tumor composition from multi-sample sequencing data. *Bioinformatics* **31**, i62–i70 (2015).

[28] El-Kebir, M., Satas, G., Oesper, L. & Raphael, B. J. Inferring the mutational history of a tumor using multi-state perfect phylogeny mixtures. *Cell Systems* **3**, 43–53 (2016).

[29] Staaf, J. *et al.* Segmentation-based detection of allelic imbalance and loss-of-heterozygosity in cancer cells using whole genome snp arrays. *Genome Biology* **9**, R136 (2008).

[30] Cheng, Y. *et al.* Quantification of multiple tumor clones using gene array and sequencing data. *The Annals of Applied Statistics* **11**, 967 (2017).

[31] Wang, L. *et al.* Novel somatic and germline mutations in intracranial germ cell tumours. *Nature* **511**, 241 (2014).

[32] Carter, S., Meyerson, M. & Getz, G. Accurate estimation of homologue-specific DNA concentration-ratios in cancer samples allows long-range haplotyping. Preprint at: `https://doi.org/10.1038/npre.2011.6494.1` (2011).

[33] Hughes, M. C. & Sudderth, E. Memoized online variational inference for dirichlet process mixture models. In *Advances in Neural Information Processing Systems*, 1133–1141 (2013).

[34] Zack, T. I. *et al.* Pan-cancer patterns of somatic copy number alteration. *Nature Genetics* **45**, 1134 (2013).

[35] Jamal-Hanjani, M. *et al.* Tracking the evolution of non–small-cell lung cancer. *New England Journal of Medicine* **376**, 2109–2121 (2017).

[36] Letouzé, E. *et al.* Mutational signatures reveal the dynamic interplay of risk factors and cellular processes during liver tumorigenesis. *Nature Communications* **8**, 1315 (2017).

[37] Lubiw, A. The boolean basis problem and how to cover some polygons by rectangles. *SIAM Journal on Discrete Mathematics* **3**, 98–115 (1990).

[38] Vavasis, S. A. On the complexity of nonnegative matrix factorization. *SIAM Journal on Optimization* **20**, 1364–1377 (2009).

[39] Nijenhuis, A. & Wilf, H. S. *Combinatorial algorithms: for computers and calculators* (Elsevier, 2014).

[40] Sherali, H. D. & Smith, J. C. Improving discrete model representations via symmetry considerations. *Management Science* **47**, 1396–1407 (2001).

[41] Thorndike, R. L. Who belongs in the family? *Psychometrika* **18**, 267–276 (1953).

[42] Salvador, S. & Chan, P. Determining the number of clusters/segments in hierarchical clustering/segmentation algorithms. In *16th IEEE International Conference on Tools with Artificial Intelligence*, 576–584 (IEEE, 2004).

[43] Satopaa, V., Albrecht, J., Irwin, D. & Raghavan, B. Finding a" kneedle" in a haystack: Detecting knee points in system behavior. In *2011 31st international conference on distributed computing systems workshops*, 166–171 (IEEE, 2011).

[44] Milligan, G. W. & Cooper, M. C. An examination of procedures for determining the number of clusters in a data set. *Psychometrika* **50**, 159–179 (1985).

[45] Krzanowski, W. J. & Lai, Y. A criterion for determining the number of groups in a data set using sum-of-squares clustering. *Biometrics* 23–34 (1988).

[46] Ester, M., Kriegel, H.-P., Sander, J., Xu, X. *et al.* A density-based algorithm for discovering clusters in large spatial databases with noise. In *Kdd*, vol. 96 (34), 226–231 (1996).

[47] Tibshirani, R., Walther, G. & Hastie, T. Estimating the number of clusters in a data set via the gap statistic. *Journal of the Royal Statistical Society: Series B (Statistical Methodology)* **63**, 411–423 (2001).

[48] Dimitriadou, E., Dolničar, S. & Weingessel, A. An examination of indexes for determining the number of clusters in binary data sets. *Psychometrika* **67**, 137–159 (2002).

[49] Hothorn, T. & Everitt, B. S. *A handbook of statistical analyses using R* (Chapman and Hall/CRC, 2014).

[50] Zhang, Y., Mańdziuk, J., Quek, C. H. & Goh, B. W. Curvature-based method for determining the number of clusters. *Information Sciences* **415**, 414–428 (2017).

[51] Antunes, M., Gomes, D. & Aguiar, R. L. Knee/elbow estimation based on first derivative threshold. In *2018 IEEE Fourth International Conference on Big Data Computing Service and Applications (BigDataService)*, 237–240 (IEEE, 2018).

[52] Adalsteinsson, V. A. *et al.* Scalable whole-exome sequencing of cell-free DNA reveals high concordance with metastatic tumors. *Nature Communications* **8**, 1324 (2017).

[53] Ivakhno, S. *et al.* tHapMix: simulating tumour samples through haplotype mixtures. *Bioinformatics* **33**, 280–282 (2017).

[54] Ewing, A. D. *et al.* Combining tumor genome simulation with crowdsourcing to benchmark somatic single-nucleotide-variant detection. *Nature Methods* **12**, 623 (2015).

[55] Salcedo, A. *et al.* A community effort to create standards for evaluating tumor subclonal reconstruction. *Nature Biotechnology* **38**, 97–107 (2020).

[56] Gusnanto, A., Wood, H. M., Pawitan, Y., Rabbitts, P. & Berri, S. Correcting for cancer genome size and tumour cell content enables better estimation of copy number alterations from next-generation sequence data. *Bioinformatics* **28**, 40–47 (2011).

[57] Hu, X. *et al.* pIRS: Profile-based illumina pair-end reads simulator. *Bioinformatics* **28**, 1533–1535 (2012).

[58] Escalona, M., Rocha, S. & Posada, D. A comparison of tools for the simulation of genomic next-generation sequencing data. *Nature Reviews Genetics* **17**, 459 (2016).

[59] Huang, W., Li, L., Myers, J. R. & Marth, G. T. ART: a next-generation sequencing read simulator. *Bioinformatics* **28**, 593–594 (2012).

[60] Li, H. & Durbin, R. Fast and accurate short read alignment with Burrows–Wheeler transform. *Bioinformatics* **25**, 1754–1760 (2009).

[61] DePristo, M. A. *et al.* A framework for variation discovery and genotyping using next-generation DNA sequencing data. *Nature Genetics* **43**, 491 (2011).

[62] Howie, B. N., Donnelly, P. & Marchini, J. A flexible and accurate genotype imputation method for the next generation of genome-wide association studies. *PLoS Genetics* **5**, e1000529 (2009).

[63] Ha, G. *et al.* Integrative analysis of genome-wide loss of heterozygosity and monoallelic expression at nucleotide resolution reveals disrupted pathways in triple-negative breast cancer. *Genome Research* (2012).

[64] Dentro, S. C. *et al.* Characterizing genetic intra-tumor heterogeneity across 2,658 human cancer genomes. Preprint at: `https://doi.org/10.1101/312041` (2018).

[65] Urrutia, E., Chen, H., Zhou, Z., Zhang, N. R. & Jiang, Y. Integrative pipeline for profiling DNA copy number and inferring tumor phylogeny. *Bioinformatics* **34**, 2126–2128 (2018).

[66] Gurobi Optimization, L. Gurobi optimizer reference manual (2019). URL `http://www.gurobi.com`.

[67] Nathanson, S. D. Insights into the mechanisms of lymph node metastasis. *Cancer* **98**, 413–423 (2003).

[68] Naxerova, K. *et al.* Origins of lymphatic and distant metastases in human colorectal cancer. *Science* **357**, 55–60 (2017).

[69] Nagtegaal, I. D. & Schmoll, H.-J. Colorectal cancer: What is the role of lymph node metastases in the progression of colorectal cancer? *Nature Reviews Gastroenterology and Hepatology* **14**, 633 (2017).

[70] Gao, R. *et al.* Punctuated copy number evolution and clonal stasis in triple-negative breast cancer. *Nature Genetics* **48**, 1119 (2016).

[71] Raphael, B. J. *et al.* Integrated genomic characterization of pancreatic ductal adenocarcinoma. *Cancer Cell* **32**, 185–203 (2017).

[72] Bielski, C. M. *et al.* Genome doubling shapes the evolution and prognosis of advanced cancers. *Nature Genetics* **50**, 1189 (2018).

[73] Koboldt, D. C. *et al.* VarScan: variant detection in massively parallel sequencing of individual and pooled samples. *Bioinformatics* **25**, 2283–2285 (2009).

[74] Koboldt, D. C. *et al.* VarScan 2: somatic mutation and copy number alteration discovery in cancer by exome sequencing. *Genome Research* **22**, 568–576 (2012).

[75] Deshwar, A. G. *et al.* PhyloWGS: reconstructing subclonal composition and evolution from whole-genome sequencing of tumors. *Genome Biology* **16**, 35 (2015).
