# Supplementary material for: Accurate quantification of copy-number aberrations and whole-genome duplications in multi-sample tumor sequencing data
Source: Nat Commun. 2020 Sep 2;11:4301. doi: 10.1038/s41467-020-17967-y (PMC7468132; doi:10.1038/s41467-020-17967-y)
Supplement: Supplementary file 3 — Reporting Summary [file 41467_2020_17967_MOESM3_ESM.pdf]

NCOMMS-20-03751

Corresponding author(s): Benjamin J. Raphael

Last updated by author(s): Jul 13, 2020

Reporting Summary

Nature Research wishes to improve the reproducibility of the work that we publish. This form provides structure for consistency and transparency in reporting. For further information on Nature Research policies, see Authors & Referees and the Editorial Policy Checklist.

Statistics

For all statistical analyses, confirm that the following items are present in the figure legend, table legend, main text, or Methods section.

n/a	Confirmed	
☐	☒	The exact sample size (*n*) for each experimental group/condition, given as a discrete number and unit of measurement
☐	☒	A statement on whether measurements were taken from distinct samples or whether the same sample was measured repeatedly
☐	☒	The statistical test(s) used AND whether they are one- or two-sided<br>*Only common tests should be described solely by name; describe more complex techniques in the Methods section.*
☒	☐	A description of all covariates tested
☐	☒	A description of any assumptions or corrections, such as tests of normality and adjustment for multiple comparisons
☐	☒	A full description of the statistical parameters including central tendency (e.g. means) or other basic estimates (e.g. regression coefficient) AND variation (e.g. standard deviation) or associated estimates of uncertainty (e.g. confidence intervals)
☐	☒	For null hypothesis testing, the test statistic (e.g. *F*, *t*, *r*) with confidence intervals, effect sizes, degrees of freedom and *P* value noted<br>*Give P values as exact values whenever suitable.*
☒	☐	For Bayesian analysis, information on the choice of priors and Markov chain Monte Carlo settings
☒	☐	For hierarchical and complex designs, identification of the appropriate level for tests and full reporting of outcomes
☒	☐	Estimates of effect sizes (e.g. Cohen's *d*, Pearson's *r*), indicating how they were calculated

Our web collection on statistics for biologists contains articles on many of the points above.

Software and code

Policy information about availability of computer code

Data collection	No software for data collection was used.
Data analysis	We developed HATCHet to infer allele- and clone-specific CNAs and WGDs jointly from the sequencing data of one or more multiple samples from the same patient. HATCHet is publicly available on GitHub at https://github.com/raphael-group/hatchet. We developed MASCoTE to simulate multi-sample DNA sequencing datasets in this study. MASCoTE is publicly available on GitHub at https://github.com/raphael-group/mascote. SAMtools (v1.7) was used to count the number of aligned sequencing reads from BAM files. BCFtools (v1.7) was used to identify germline heterozygous SNPs from matched-normal samples. Varscan 2 (v2.3.9) was used to identify somatic SNVs and small indels.

For manuscripts utilizing custom algorithms or software that are central to the research but not yet described in published literature, software must be made available to editors/reviewers. We strongly encourage code deposition in a community repository (e.g. GitHub). See the Nature Research guidelines for submitting code & software for further information.

Data

Policy information about availability of data

All manuscripts must include a data availability statement. This statement should provide the following information, where applicable:

- Accession codes, unique identifiers, or web links for publicly available datasets
- A list of figures that have associated raw data
- A description of any restrictions on data availability

Whole-genome DNA sequencing data for the prostate and pancreas cancer datasets analyzed in this study are available from the European Genome-phenome Archive (EGA) under accession numbers EGAS00001000262 [https: //ega-archive.org/studies/EGAS00001000262] and EGAS00001002186 [https://ega-archive.org/studies/ EGAS00001002186], respectively. Whole-exome DNA sequencing data for breast cancer patients in Kim et al. [Ref 45] and Casasent et al. [Ref 46] are available from the NCBI Sequence Read Archive (SRA) under accession numbers SRP114962 [https://www.ncbi.nlm.nih.gov/sra/?term=SRP114962] and SRP116771 [https://www.ncbi.nlm. nih.gov/sra/?term=SRP116771]. All the processed simulated data, the results of all methods on simulated data, and the results of HATCHet

on the prostate and pancreas cancer datasets are available on GitHub from https: //github.com/raphael-group/hatchet-paper and on Zenodo from https://doi.org/10.5281/zenodo.3830088.

Field-specific reporting

Please select the one below that is the best fit for your research. If you are not sure, read the appropriate sections before making your selection.

☒ Life sciences ☐ Behavioural & social sciences ☐ Ecological, evolutionary & environmental sciences

For a reference copy of the document with all sections, see nature.com/documents/nr-reporting-summary-flat.pdf

Life sciences study design

All studies must disclose on these points even when the disclosure is negative.

Sample size	No experimental data was generated for this study. Instead, we analyzed previously published sequencing datasets of metastatic prostate cancer [Gundem et al., Nature 2015] and metastatic pancreas cancer [Makohon-Moore et al., Nature genetics, 2017] patients.
Data exclusions	No experimental data was generated for this study. In this study we further excluded one sample (A21-F) from the metastatic prostate cancer dataset [Gundem et al., Nature 2015] because it exhibited outlying values of RDR and BAF.
Replication	No experimental data was generated for this study. For reproducibility, all the processed simulated data, the results of all methods on simulated data, and the results of HATCHet on the prostate and pancreas cancer datasets are available on GitHub at https://github.com/raphael-group/hatchet-paper and on Zenodo at https://doi.org/10.5281/zenodo.3830088.
Randomization	No experimental data was generated for this study.
Blinding	No experimental data was generated for this study.

Reporting for specific materials, systems and methods

We require information from authors about some types of materials, experimental systems and methods used in many studies. Here, indicate whether each material, system or method listed is relevant to your study. If you are not sure if a list item applies to your research, read the appropriate section before selecting a response.

Materials & experimental systems

n/a	Involved in the study	
☒	☐	Antibodies
☒	☐	Eukaryotic cell lines
☒	☐	Palaeontology
☒	☐	Animals and other organisms
☒	☐	Human research participants
☒	☐	Clinical data

Methods

n/a	Involved in the study	
☒	☐	ChIP-seq
☒	☐	Flow cytometry
☒	☐	MRI-based neuroimaging
